# Model-based contextualization of in vitro toxicity data quantitatively predicts in vivo drug response in patients

**DOI:** 10.1007/s00204-016-1723-x

**Published:** 2016-05-09

**Authors:** Christoph Thiel, Henrik Cordes, Isabel Conde, José Vicente Castell, Lars Mathias Blank, Lars Kuepfer

**Affiliations:** 1 iAMB - Institute of Applied Microbiology, ABBt - Aachen Biology and Biotechnology, RWTH Aachen University, Worringerweg 1, 52074 Aachen, Germany; 2 Unidad de Hepatología Experimental y Trasplante Hepático, Instituto de Investigación Sanitaria La Fe, 46026 Valencia, Spain

**Keywords:** Quantitative systems pharmacology, Pharmacokinetic modeling, PBPK, Transcriptomics, Clinical translation, Drug-induced liver injury, Multiscale modeling

## Abstract

Understanding central mechanisms underlying drug-induced toxicity plays a crucial role in drug development and drug safety. However, a translation of cellular in vitro findings to an actual in vivo context remains challenging. Here, physiologically based pharmacokinetic (PBPK) modeling was used for in vivo contextualization of in vitro toxicity data (PICD) to quantitatively predict in vivo drug response over time by integrating multiple levels of biological organization. Explicitly, in vitro toxicity data at the cellular level were integrated into whole-body PBPK models at the organism level by coupling in vitro drug exposure with in vivo drug concentration–time profiles simulated in the extracellular environment within the organ. PICD was exemplarily applied on the hepatotoxicant azathioprine to quantitatively predict in vivo drug response of perturbed biological pathways and cellular processes in rats and humans. The predictive accuracy of PICD was assessed by comparing in vivo drug response predicted for rats with observed in vivo measurements. To demonstrate clinical applicability of PICD, in vivo drug responses of a critical toxicity-related pathway were predicted for eight patients following acute azathioprine overdoses. Moreover, acute liver failure after multiple dosing of azathioprine was investigated in a patient case study by use of own clinical data. Simulated pharmacokinetic profiles were therefore related to in vivo drug response predicted for genes associated with observed clinical symptoms and to clinical biomarkers measured in vivo. PICD provides a generic platform to investigate drug-induced toxicity at a patient level and thus may facilitate individualized risk assessment during drug development.

**Electronic supplementary material:**

The online version of this article (doi:10.1007/s00204-016-1723-x) contains supplementary material, which is available to authorized users.

## Introduction

Drug-induced toxicity is a major clinical problem (Schuster et al. 2005) with cardiotoxicity and hepatotoxicity being the most frequent clinical cases (Von Hoff et al. 1977; Andrade et al. 2005; Takikawa et al. 2009). The predictability of specific toxic events is a major challenge in pharmaceutical development since the underlying origins are almost unforeseeable (Kaplowitz 2004). In drug development, whole-body physiologically based pharmacokinetic (PBPK) models are nowadays routinely used (Jones et al. 2006; Maharaj et al. 2013; Lippert et al. 2013). Whole-body PBPK modeling describes biological processes underlying drug pharmacokinetics at a large scale of physiological detail and may be used among others to simulate interstitial concentration–time profiles in the extracellular environment of various organs (Jones et al. 2009; Kuepfer 2010). PBPK modeling aims for a mechanistic understanding of physiological processes describing drug absorption, distribution, metabolism and elimination (ADME) within the body based on prior physiological and anatomical knowledge. Different organs are explicitly represented in PBPK models and are connected by blood flow (Fig. S1). Since PBPK models describe the physiology of an organism at a high level of detail, they can be used to simulate pharmacokinetic (PK) profiles of specific patient subgroups with individualized physiology (Maharaj et al. 2013; Lippert et al. 2013).

In order to detect drug-induced injury at an early stage, reliable predictions of toxic events as well as representative diagnostic biomarkers are of key relevance for patient safety (Shi et al. 2010). This also requires a mechanistic understanding of the underlying cellular processes (Bissell et al. 2001; Schimmel et al. 2004; Holt and Ju 2006; Russmann et al. 2009). Current advances in systems toxicology provide novel insights into central mechanisms involved in drug-induced toxicity (Waters and Fostel 2004; Heijne et al. 2005; Chen et al. 2012). Changes at different biological levels can nowadays be measured by -omics technologies to describe cellular alterations in response to toxic drug concentrations. Transcriptome profiling was successfully applied before to study adverse effects of toxic agents (Hockley et al. 2006; Brynildsen and Liao 2009; Michaelson et al. 2011; Zhang et al. 2012; Van Delft et al. 2012; Iskar et al. 2013; Doktorova et al. 2013; Zhang et al. 2014; Herpers et al. 2015). Combined application of different profiling techniques allows linking cellular changes at multiple levels of biological organization that finally facilitates the characterization of molecular mechanisms of toxic events (Carreras Puigvert et al. 2013; Wilmes et al. 2013; Pillai et al. 2014). Furthermore, reverse toxicokinetics were used before to identify steady state blood concentrations for correlations of in vivo equivalent doses with in vitro bioactivity data (Dix et al. 2007; Judson et al. 2011; Wetmore et al. 2013; Judson et al. 2014). In another study, physiologically based kinetic models developed for different glycol ethers were used to estimate dose–response curves in rats and humans (Louisse et al. 2010). However, a systematic consideration of in vitro toxicity data into an in vivo context, thereby reflecting temporal cellular changes induced by drugs administered in vivo, remains still challenging.

In this article, PBPK-based in vivo contextualization of in vitro toxicity data (PICD) is presented. PICD integrates in vitro toxicity data into drug-specific whole-body PBPK models to translate drug-induced in vitro findings to an actual in vivo situation, thereby predicting drug-specific response profiles induced by different dose levels administered in vivo. At the cellular level, in vitro toxicity data are coupled with equivalent PBPK-simulated concentration–time profiles at the organism level to allow a quantitative description of time-resolved in vivo drug response of key cellular processes and biological pathways. Applying PICD in clinical research allows the quantitative prediction of patient-specific drug response by specifically incorporating patient physiology in individualized PBPK models. In brief, PICD aims for a translation of preclinical in vitro toxicity data into an in vivo context and hence allows risk assessment for individual patients during drug development.

**Fig. 1:**
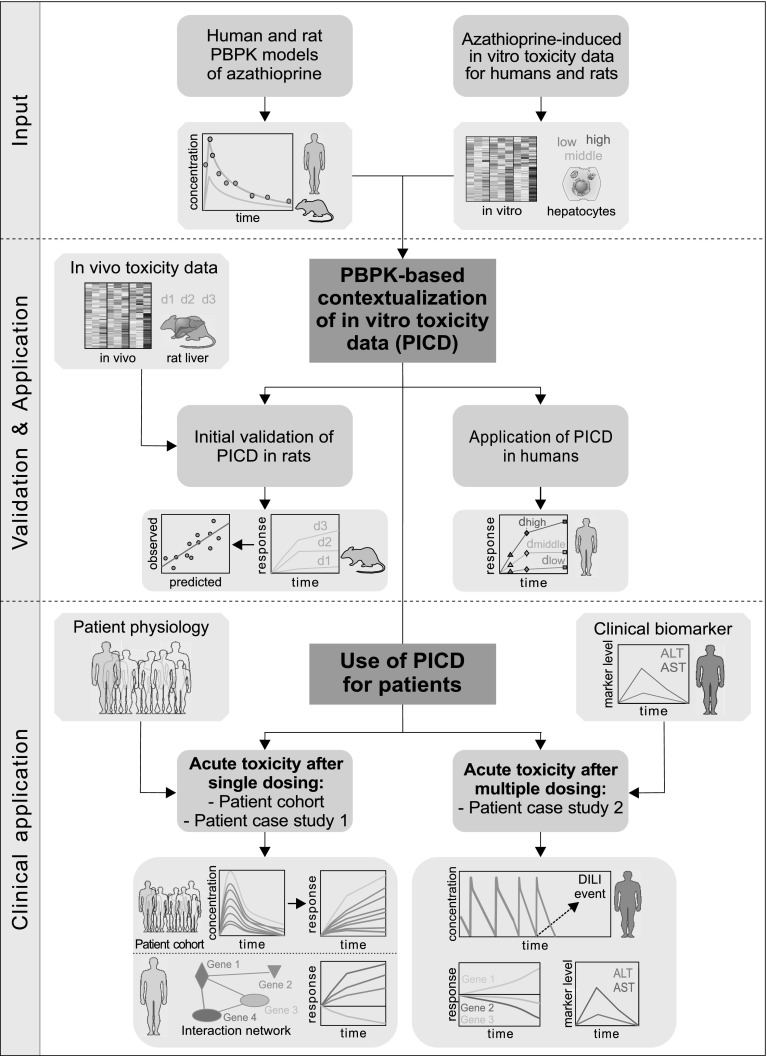
Overview of the use of PICD. *Input* Human and rat PPBK models of azathioprine were developed and in vitro toxicity data of primary human and rat hepatocytes were analyzed (Igarashi et al. 2015). *Validation and Application* To validate PICD, in vivo toxicity data obtained in rat livers were used to compare predicted in vivo drug response with measurements observed in vivo. PICD was then applied in humans, thereby predicting drug response for in vivo doses estimated for concentrations used in vitro. *Clinical application* To demonstrate clinical applicability, PICD was applied on different clinical cases. At first, patient physiology of eight clinical cases was considered in individualized PBPK models to predict in vivo drug response induced by different azathioprine overdoses (Gregoriano et al. 2014). One patient was further regarded in a patient case study using own data, thereby predicting in vivo response of genes involved in critical processes of an interaction network. Moreover, acute toxicity after multiple dosing of azathioprine at therapeutic dose was investigated in a second patient case study. Therefore, drug concentrations simulated for the entire therapy process were related to in vivo response predicted for symptoms-related genes and to clinical biomarkers measured in the specific patient

PICD is exemplarily applied on the hepatotoxicant azathioprine in humans and rats. As input, human and rat PBPK models of azathioprine are developed and in vitro toxicity data are analyzed (Fig. 1). Explicitly, time series gene expression profiles of primary human and rat hepatocytes from Open TG-GATEs (Igarashi et al. 2015), a large-scale toxicogenomics database, represent the in vitro toxicity data. The predictive quality of PICD is assessed by in vivo response data measured in rat livers (Igarashi et al. 2015), thus exploring whether predicted in vivo drug response shows in vivo relevance (Fig. 1). To assess the predictive accuracy of PICD, in vivo data are necessary for validation purposes. Since in vivo response data from liver biopsies were available in rats (Igarashi et al. 2015), PICD was applied on rats to assess whether predicted drug response shows in vivo relevance (Fig. 1). PICD is then applied for humans to predict in vivo drug response over time for doses estimated to be the in vivo equivalents for concentrations exposed in vitro (Fig. 1). Note that the application of PICD in rats and humans is fully independent since apart from the validation step no information from the animal study was further used for the human case. To demonstrate the potential of PICD for clinical applications in humans, acute toxicity is investigated after single and multiple dosing of azathioprine. Patient-specific in vivo drug response over time following documented cases of acute azathioprine overdose is predicted specifically considering patient physiology (Gregoriano et al. 2014; Fig. 1). The patient, who received the highest overdose (Gregoriano et al. 2014), is further considered in a first patient case study (Fig. 1). In a second patient case study, PICD is applied on own clinical data to get insights into acute toxicity after multiple dosing of azathioprine at the therapeutic dose. Simulated drug concentration–time profiles, predicted responses of symptoms-related genes as well as clinical biomarkers measured in vivo are therefore analyzed (Fig. 1).

## Results

### PBPK-based in vivo contextualization of in vitro toxicity data (PICD)

**Fig. 2:**
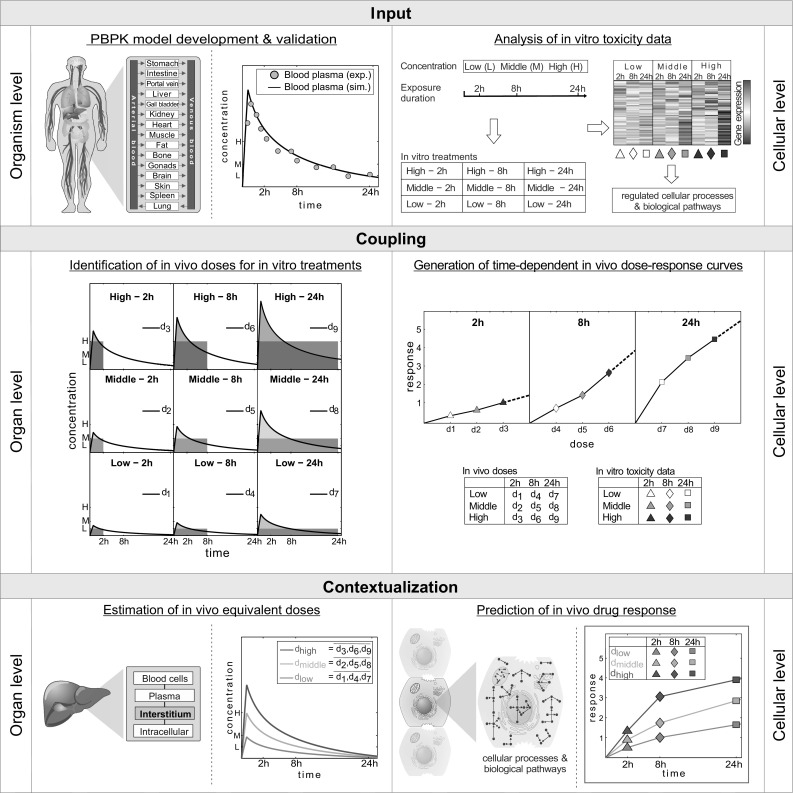
Workflow of PICD. *Input* At the organism level, PBPK models are developed at the organism level whereby simulated (sim.) blood plasma concentrations are validated with experimental (exp.) PK data. At the cellular level, in vitro toxicity data of compound-treated primary hepatocytes are analyzed (Igarashi et al. 2015). The hepatocytes were exposed to three different concentrations (*low*, *middle* and *high*). Drug-treated hepatocytes were compared to their time-matched controls to determine the change in gene expression after 2, 8 and 24 h leading to a total of nine different treatments (*white*–*gray-colored symbols*). Functional enrichment analysis was then applied to find regulated cellular processes and biological pathways. *Coupling* In vivo doses *d*_1_–*d*_9_ are identified for all treatments such that the in vivo exposure simulated in the interstitial space of the liver (*colored area* under the *curve*) matched the in vitro exposure (*gray rectangular area*). Identified in vivo doses *d*_1_–*d*_9_ together with in vitro toxicity data (*white–gray-colored symbols*) are used to generate dose–response curves for all considered time points of the in vitro experiment. *Contextualization* In vivo doses *d*_1_–*d*_9_ are averaged horizontally along the same in vitro concentration leading to three doses *d*_low_, *d*_middle_ and *d*_high_ (*colored lines*) representing the in vivo equivalents to exposed in vitro concentrations (*low*, *middle*, *high*). At the cellular level, in vivo drug response over time reflecting changes in cellular processes and biological pathways are then predicted (*colored symbols*) for the in vivo equivalent doses (*colored lines*) by using time-dependent in vivo dose–response curves (color figure online)

Here, the development of PICD—an integrative multiscale approach—is shown. The application of PICD allows predicting in vivo drug response by integrating multiple levels of biological organization, thereby using whole-body PBPK models at the organism level to couple interstitial PK profiles at the organ level with in vitro toxicity data at the cellular level (Fig. 2). The use of PICD thus allows the prediction of drug response over time in an in vivo context. Gene expression data of primary human and rat hepatocytes treated with specific drugs at different concentration levels over different time ranges from Open TG-GATEs (Igarashi et al. 2015) are used exemplarily as in vitro toxicity data to quantify drug-induced toxicity at the cellular level (Fig. 2). In the in vitro assay of TG-GATEs, the highest concentration was selected such that cell viability was decreased by 10–20 % (Igarashi et al. 2015). PICD is basically applicable on any drug of interest, provided that correspondent in vitro response data for the same compound is available. Note that the application of PICD is here exemplarily shown for the liver since the in vitro toxicity data were obtained in primary hepatocytes. To translate in vitro findings to an in vivo situation, PBPK modeling is used here to contextualize these cellular gene expression data at an organism level.

In an initial step, a drug-specific PBPK model is developed to identify in vivo doses that are directly related to in vitro drug exposure (Fig. 2). The in vitro assay setup (Igarashi et al. 2015) is explicitly represented in the PBPK models by specifically adjusting in vivo drug plasma protein binding in the PBPK model correspondent to the in vitro concentrations. PK profiles simulated in the interstitial space of the liver are then coupled with in vitro toxicity data to predict in vivo drug response at the cellular level following in vivo drug administration at the organism level (Fig. 2).

To couple interstitial concentration–time profiles with in vitro toxicity data, in vivo doses are identified by PBPK simulations for intravenous drug administration such that the in vitro drug exposure in the assay equals the interstitial area under the curve in the liver at each experimental time point (Fig. 2). Note that by using validated PBPK models, potential non-linearities in ADME processes affecting the interstitial drug concentration are implicitly considered such that dose estimations are accurate across different dosage regimens. Dose–response curves are then generated for all time points by mapping in vitro toxicity data to the identified in vivo doses (Fig. 2).

The identified in vivo doses are averaged horizontally to three doses (*d*_low_, *d*_middle_ and *d*_high_), which thus represent the in vivo equivalents to in vitro concentrations (low, middle and high). Drug response values are next calculated and assigned to doses *d*_low_, *d*_middle_ and *d*_high_ by linearly interpolating dose–response curves (Fig. 2) to predict in vivo drug response in relevant gene ontology (GO) (Ashburner et al. 2000) terms, as well as in human pathways from the Kyoto Encyclopedia of Genes and Genomes (KEGG) (Kanehisa and Goto 2000) and in toxicity-related pathways (TOX) (SABiosciences) (Table S1).

The use of PICD enables a time-resolved description of drug-induced in vivo response at the organism level by the integration of several levels of biological organization and hence allows considering various aspects of translational research in drug development.

### Use of PICD for patients

**Fig. 3:**
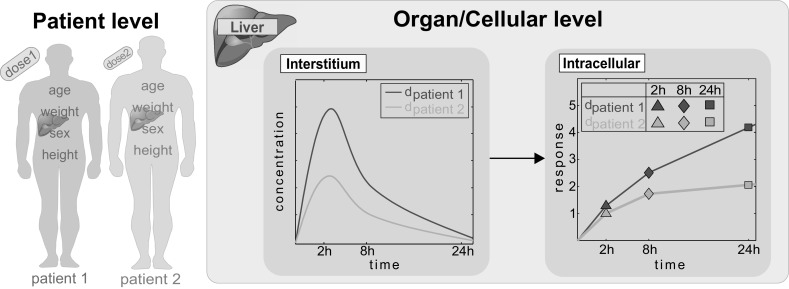
Use of PICD for patients. At the patient level, individualized PBPK models are developed by incorporating anthropometric parameters of patients (e.g., weight). PICD is then individually applied on each patient-specific PBPK model by taking into account the respective dosage regimen (administration route and dose level). Concentration–time profiles are therefore simulated in the interstitial space of the liver and correspondent in vivo drug response profiles are predicted at the cellular level following administration of the specific dose

PBPK modeling allows among others the consideration of patient-specific differences in the anatomy and physiology between various individuals by incorporating the anthropometry of patients (e.g., body weight). Moreover, since validated PBPK models allow extrapolating PK simulations to different dosage regimens, PICD is applicable to predict drug response not only for the in vivo equivalent doses administered intravenously (Fig. 2) but also for other dose levels and administration routes. Thus, PICD can be applied in a patient-specific manner to allow the simulation and interpretation of clinical observations following drug administration over time at the patient level (Fig. 3). Anthropometric parameters of patients (e.g., age or weight) are therefore used to build individualized PBPK models specifically considering patient physiology. Time-dependent dose–response curves (Fig. 2) are generated analogously for each clinical case by simulating PK profiles in the interstitial space of the liver taking into account the specific administration route (Fig. 3). Finally, patient-specific in vivo drug response can be predicted following administration of the respective dosage regimen in each patient (Fig. 3). The application of PICD therefore facilitates the consideration of in vitro toxicity data within the context of human patients described in turn by patient-specific PBPK models (Maharaj et al. 2013; Lippert et al. 2013). PICD thus provides a generic platform for translational research in clinical drug development.

### Organism level: PBPK models of azathioprine for humans and rats

**Fig. 4:**
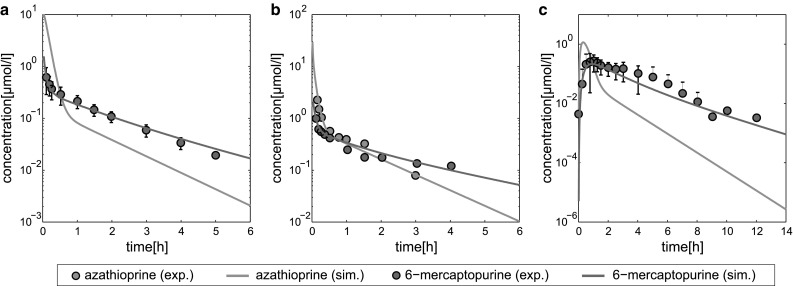
PBPK model development and validation. Simulated concentration–time curves (*lines*) for azathioprine (*blue*) and 6-mercaptopurine (*red*) were assessed with experimental PK profiles (*circles*) (Van Os et al. 1996). The reference PBPK model was then validated by evaluating simulated PK profiles with experimental PK data from different clinical studies (Odlind et al. 1986; Zins et al. 1997) (Table S2) not used to establish the reference model. Azathioprine was either administered intravenously (IV) or orally (PO). **a** Reference, 50 mg IV. **b** Validation, 100 mg IV. **c** Validation, 100 mg PO (color figure online)

At the organism level, PICD initially requires the establishment of validated PBPK models (Fig. 2). The immunosuppressant azathioprine (Elion 1993) was chosen here as an exemplary use case for a hepatotoxic compound since the drug label gives a severity level of 3 for drug-induced liver injury (DILI) (Chen et al. 2011; Björnsson 2015). Compound-specific physicochemical properties and the unbound fraction of the drug in plasma (*F*_U_) were used to parametrize the initial reference PBPK model for humans (Fig. 4; Table 1). Furthermore, patient physiology was considered in the human PBPK model to characterize the specific patient physiology (Table 2; Odlind et al. 1986; Van Os et al. 1996; Zins et al. 1997). The compound-specific parameters (Table 1) together with the specific information about the clinical studies (Table 2; Odlind et al. 1986; Van Os et al. 1996; Zins et al. 1997) are sufficient to reproduce the PBPK models of azathioprine since all anatomical and physiological parameters for both rats and humans are already provided in the modeling software. Likewise compound-specific parameters such as membrane permeabilities or partition coefficients are directly calculated by the formulas underlying the chosen distribution model.

**Table 1:** PBPK model parameters

Drug	MW (g/mol)	pKa	log*P*	*F*_U_	Metabolic enzyme	*K*_M_ (µmol/l)	*v*_max__(_µmol/l/min)
Azathioprine	277.26 (Wishart et al. 2006)	7.87 (Wishart et al. 2006)	0.10 (Wishart et al. 2006)	0.70 (Wishart et al. 2006)	Glutathione-s-transferase A1 (Eklund et al. 2006)	7.0^a^	60.0^a^
6-Mercaptopurine	152.18 (Wishart et al. 2006)	9.50 (Strongest acidic) 2.99 (Strongest basic) (Wishart et al. 2006)	1.85 (Czyrski and Kupczyk 2013)	0.81 (Wishart et al. 2006)	Xanthine oxidase (Aberra and Lichtenstein 2005)	41.5^a^	410.0^a^

Molecular weight (MW), acid dissociation constant (pKa), octanol/water partition coefficient (log*P*), fraction unbound (*F*_U_) and integrated metabolic process consisting of metabolic enzyme and corresponding kinetic parameters (*v*_max_, *K*_M_) used for the developed PBPK model. The experimental log*P* value for 6-mercaptopurine was slightly adjusted, while the experimentally measured lipophilicity for azathioprine was used unchanged

^a^Estimated

**Table 2:** Experimental conditions

Administration route	Dose (mg)	Subjects	Usage	References
Intravenous bolus	50	Healthy (*n* = 24)	Reference	(Van Os et al. 1996)
Intravenous bolus	100	Uremic patient (*n* = 1)	Validation	(Odlind et al. 1986)
Oral	100	Healthy (*n* = 10)	Validation	(Zins et al. 1997)

Administration route, respective doses, health state and number of subjects. The experimental PK data were either used for establishment of the reference PBPK model (references) or for model validation (validation)

First, plasma concentration data were used for initial model establishment (Van Os et al. 1996). The PBPK model considered both the prodrug azathioprine that is quickly converted in the liver by glutathione-s-transferase (Kaplowitz and Kuhlenkamp 1978; Watanabe et al. 1978; Eklund et al. 2006) and its metabolite 6-mercaptopurine, which is in turn mostly metabolized by xanthine oxidase to 6-thiouric acid (Aberra and Lichtenstein 2005). Since a negligible amount of both drugs, azathioprine and 6-mercaptopurine, was excreted unchanged in urine (Elion 1972; Bergan et al. 1994), renal elimination was not considered in the underlying PBPK model. To appropriately validate simulated concentration–time profiles of different compounds, experimental PK data are necessary. The metabolite 6-thiouric acid was not included in the PBPK model because no experimental measurements were performed in the used clinical studies.

After model establishment, the simulated plasma concentrations showed an excellent agreement with clinical PK data used for the initial model identification (Fig. S2; Table 2). For model validation, additional experimental PK data were next used, which were accurately described without further model modifications (Table 2; Fig. S2), thereby ensuring an adequate quality of the PBPK model for further predictions.

The validated human PBPK model was next used to develop a PBPK model for rats that is needed for the initial validation of PICD. Recently, it was shown that species-specific physiology has the highest influence on the predictive quality of PBPK-based cross-species extrapolation (Thiel et al. 2015). The rat PBPK model of azathioprine (Fig. S3) was hence developed by considering species-specific differences in the physiology and anatomy in the human PBPK model of azathioprine (Fig. 4; Table 1).

### Cellular level: azathioprine-induced in vitro toxicity data for humans and rats

At the cellular level, in vitro toxicity data is required for PICD to predict in vivo drug response over time. Human and rat gene expression and enrichment analysis was performed in the same way. Time course gene expression profiles of primary human and rat hepatocytes from Open TG-GATEs were analyzed to obtain quantitative toxicity data of azathioprine (Igarashi et al. 2015). Notably, toxicity data generated by other profiling techniques (Waters and Fostel 2004; Heijne et al. 2005) can analogously be used to predict drug-specific response profiles. For each treatment, subsets of differentially expressed genes were identified (absolute fold change >1.5, Benjamini–Hochberg corrected *p* < 0.01; Fig. S4A, Fig. S4B). Functional enrichment analysis was then applied to find significantly overrepresented terms (GO) and pathways (KEGG, TOX) (Benjamini–Hochberg corrected *p* < 0.01; Data S1). Gene response values defined as absolute log2 fold change were calculated to quantify changes in significantly affected terms and pathways. Since the drug response values reflect the extent of activation or inhibition of functionally related genes in an in vivo situation, they were used to predict drug-induced cellular changes over time in both rats and humans.

### Initial validation of PICD in rats

To assess the predictive accuracy of PICD, in vivo toxicity data measured in rat livers (Fig. S4C) were used. The developed rat PBPK model (Fig. S3) together with the in vitro toxicity data obtained in rat hepatocytes (Fig. S4B) served as input for the application of PICD to predict in vivo drug response in rats. When applying PICD on rats, a corresponding in vivo dose was determined for each of the nine in vitro treatments (e.g., high—8 h; Table S2). In the in vivo rat study, the minimum toxic dose identified in a 4-week toxicity study was used as highest dose while the low and middle dose were selected by diluting the high dose with a factor of three and ten, respectively (Igarashi et al. 2015). Consequently, PICD was applied to predict drug responses induced by the three doses used in the in vivo rat study.

**Fig. 5:**
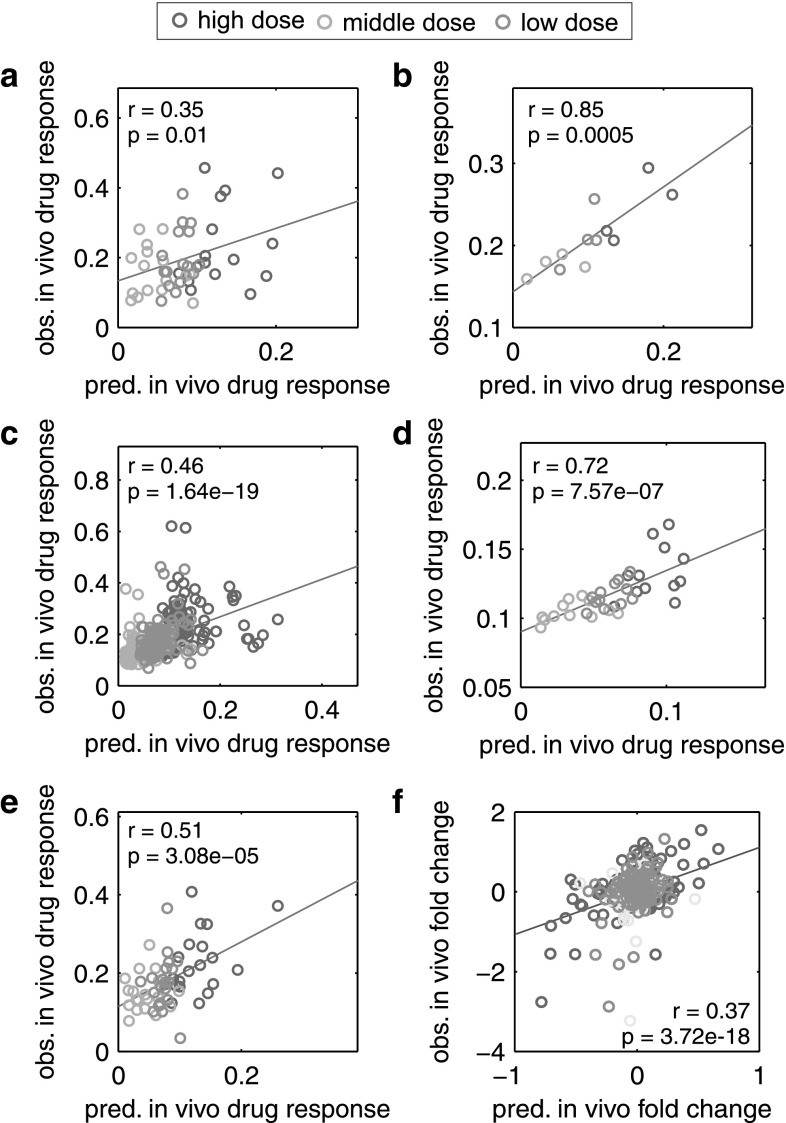
Correlation of predicted drug response profiles with in vivo measurements in rats. Correlation between predicted (pred.) in vivo profiles of drug response and gene expression with observed (obs.) profiles measured in vivo following oral administration of the three doses used in the rat study (low dose = *yellow*, middle dose = *blue*, high dose = *red*) (Igarashi et al. 2015). All cellular processes or biological pathways that were significantly regulated in at least one treatment (Data S1) and all genes analyzed in the case studies (Table S4, Table S5) were considered for the correlation of drug response and gene expression, respectively. Correlation analyses were performed by calculating Pearson’s correlation coefficient *r* and the corresponding *p* value *p*. **a** Correlation of affected KEGG pathways. **b** Correlation of affected toxicity-related pathways. **c** Correlation of affected biological processes. **d** Correlation of affected cellular components. **e** Correlation of affected molecular functions. **f** Correlation of genes considered in both case studies (color figure online)

In vivo drug response of cellular processes and biological pathways significantly regulated in rats (Data S1) were then predicted for all three doses orally administered in the in vivo rat study and were subsequently correlated with corresponding in vivo observations (Pearson’s *r* = 0.35–0.85, *p* ≤ 0.01; Fig. 5a–e; Igarashi et al. 2015). To check whether the application of PICD actually improved predictions in vivo compared to the in vitro situation, temporal in vitro patterns and predicted in vivo drug responses were both correlated with respective in vivo observations. In vitro drug response profiles of perturbed biological pathways (KEGG, TOX) and biological processes showed almost no relevance for the in vivo situation (Pearson’s *r* = −0.2 to 0.36, *p* > 0.05). In contrast, applying PICD obviously increased the concordance with in vivo measurements for all biological pathways and cellular processes (*r* = 0.2–0.77, *p* = 0.02–0.34; Fig. S5A–D). The correlation results for the individual pathways and cellular processes can be found in Data S2.

In both patient case studies, expression profiles of considered genes were predicted for clinically relevant doses to investigate acute liver toxicity after single and multiple dosing of azathioprine. To test whether predictions have in vivo relevance in rats, predicted gene expression profiles were correlated with respective profiles observed in vivo (Pearson’s *r* = 0.37, *p* = 3.7e – 18; Fig. 5f). In vitro–in vivo extrapolation of gene expression profiles was also improved by using PICD (Fig. S5F). The in vivo relevance of predictions in rats thus verified the application of PICD in humans. Independent of the use of PICD in rats, reliable in vivo profiles of drug response and gene expression were predicted following administration of azathioprine in humans.

### Application of PICD in humans

In order to now predict in vivo drug response in humans, in vitro toxicity data from azathioprine-treated hepatocytes (Fig. S4A) were coupled with interstitial PK profiles simulated in the liver for the same drug (Fig. 2). To this end, three estimates of in vivo doses were averaged horizontally to obtain doses representing the in vivo equivalents (*d*_low_ = 20.7 mg/kg, *d*_middle_ = 53.3 mg/kg and *d*_high_ = 142.8 mg/kg) to concentrations exposed in vitro (Table S2). Since the highest in vitro concentration was defined at the onset of toxicity (Igarashi et al. 2015), administration of the identified high dose (*d*_high_) was expected to cause the experimentally observed toxic effects at the cellular level. The remaining two concentrations were further selected by diluting the toxic concentration by a factor of five and twenty-five, respectively (Igarashi et al. 2015). Interestingly, the identified low dose was seven times higher compared to the therapeutic dose (3 mg/kg) used in clinical trials (Shapiro et al. 1993). Still, various toxic effects induced by dose levels in the range of the identified doses were reported in clinical studies (Table S3; Gregoriano et al. 2014).

**Fig. 6:**
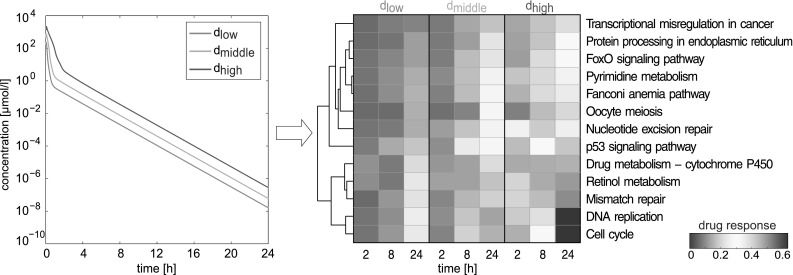
Application of PICD on the hepatotoxicant azathioprine in humans. At the organ level, liver interstitial PK profiles were simulated for doses *d*_low_, *d*_middle_ and *d*_high_ (*colored lines*). At the cellular level, correspondent drug response profiles were predicted for significant affected human pathways from KEGG following in vivo drug administration of azathioprine. The *color scale* depicts predicted in vivo drug response (color figure online)

Drug response values after 2, 8 and 24 h were next calculated to quantify the in vivo response in enriched GO terms and biological pathways (KEGG, TOX) following administration of the in vivo equivalent doses (Fig. 6, Fig. S6). By considering a dense number of hypothetical intermediate doses, correspondent in vivo drug response can be further extended to calculate drug response maps as such reflecting cellular changes over time for multiple dose levels applied in vivo (Fig. S7). In general, in vivo drug response values show low response after 2 and 8 h as opposed to larger changes after 24 h indicating a delayed regulatory response at the cellular level (Fig. 6, Fig. S6). Nonetheless, the initial increase in drug response was significant at the early time point in any regulated KEGG pathway and in 85 % of any perturbed toxicity-related pathway and GO term (*p* < 0.05, one-sample *t* test). High responsive pathways induced by all equivalent in vivo doses after 1 day showed significant increases in drug response between 8 and 24 h (DNA replication, cell cycle, mismatch repair, drug metabolism—cytochrome P450, nucleotide excision repair and retinol metabolism, *p* < 0.05, one-way ANOVA with post hoc Tukey–Kramer; Fig. 6). Furthermore, high cellular activity was identified in biological processes regulating cell replication (Fig. S6A), as well as in processes involved in chromosome condensation (Fig. S6B). Analyzing enriched toxicity-related pathways revealed high response in mechanisms related to DNA damage and repair (Fig. S6D) as suggested by another study (Karran 2006).

Here, PICD was applied to predict in vivo drug response in humans induced by in vivo doses derived from in vitro concentrations (Igarashi et al. 2015). In a next step, PICD was used for different patients by specifically considering individual physiology and various dosage regimens.

### Acute toxicity after single dosing of azathioprine: patient cohort study

**Fig. 7:**
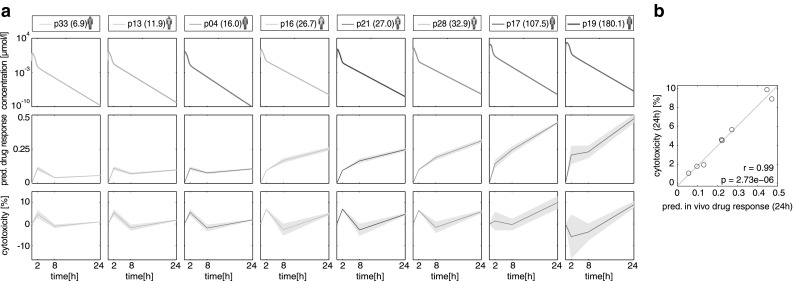
PICD applied on eight clinical cases of acute azathioprine overdose. **a** Simulated drug concentration–time profiles, corresponding predicted in vivo drug response of a critical toxicity-related pathway (DNA damage and repair), as well as predicted cytotoxicity for eight clinical cases following oral administration of different azathioprine overdoses (Table S3). In vivo drug responses and cytotoxicity were predicted for both replicates to represent the variability (*gray area*) (Igarashi et al. 2015). The mean drug responses are shown as *solid lines*. *Colors* of patients indicate the highest Poisoning Severity Score (PSS) (Persson et al. 1998) of the occurred symptoms [none (*green*) = 0, minor (*yellow*) = 1, moderate (*red*) = 2]. The overdoses (mg/kg) are shown in *brackets*. **b** Correlation results of predicted in vivo drug response of DNA damage and repair at 24 h with predicted cytotoxicity values. Correlation analysis was performed by calculating Pearson’s correlation coefficient *r* and the corresponding *p* value *p* (color figure online)

An overview of previous cases of acute azathioprine overdoses has recently been reported (Gregoriano et al. 2014). Since all cases are clinically documented, PICD could be applied to study azathioprine-induced toxicity in a patient-specific manner. In particular, individualized azathioprine PBPK models were developed by explicitly considering patient physiology (Fig. 7a; Table S3). PICD was then applied on each clinical case (Fig. 3), thereby calculating in vivo drug response of processes involved in DNA damage and repair (Fig. 7a) following oral administration of the respective overdose. In addition, correspondent cytotoxicity values describing cell viability over time were predicted for all clinical cases (Fig. 7a) to allow a correlation with patient-specific drug responses.

Analyzing patient-specific drug response profiles indicated an early increase in gene response for every patient (*p* < 0.001, one-sample *t* test; Fig. 7a). Further analysis revealed a significant change between 8 and 24 h for patient 16, 21, 28, 17 and 19 (*p* < 0.05, one-way ANOVA with post hoc Tukey–Kramer). To assess the increase in toxicity, drug response values calculated at different time points were correlated with global cytotoxicity values (Fig. 7b). Excellent correlation results were found at 24 h (*r* = 0.99, *p* = 2.73e – 6; Fig. 7b). Comparing clinically applied Poisoning Severity Score (PSS) (Persson et al. 1998) with individual drug response after 24 h confirms this observation (Spearman’s *ρ* = 0.78, *p* = 0.057). For this correlation analysis, patient 17, who remained asymptomatic after receiving a heavy overdose, was not considered (Table S3).

In the following, patient 19, who was exposed to the highest overdose (180.1 mg/kg), was regarded in a patient case study to investigate acute toxicity after single dosing of azathioprine.

### Acute toxicity after single dosing of azathioprine: patient case study 1

**Fig. 8:**
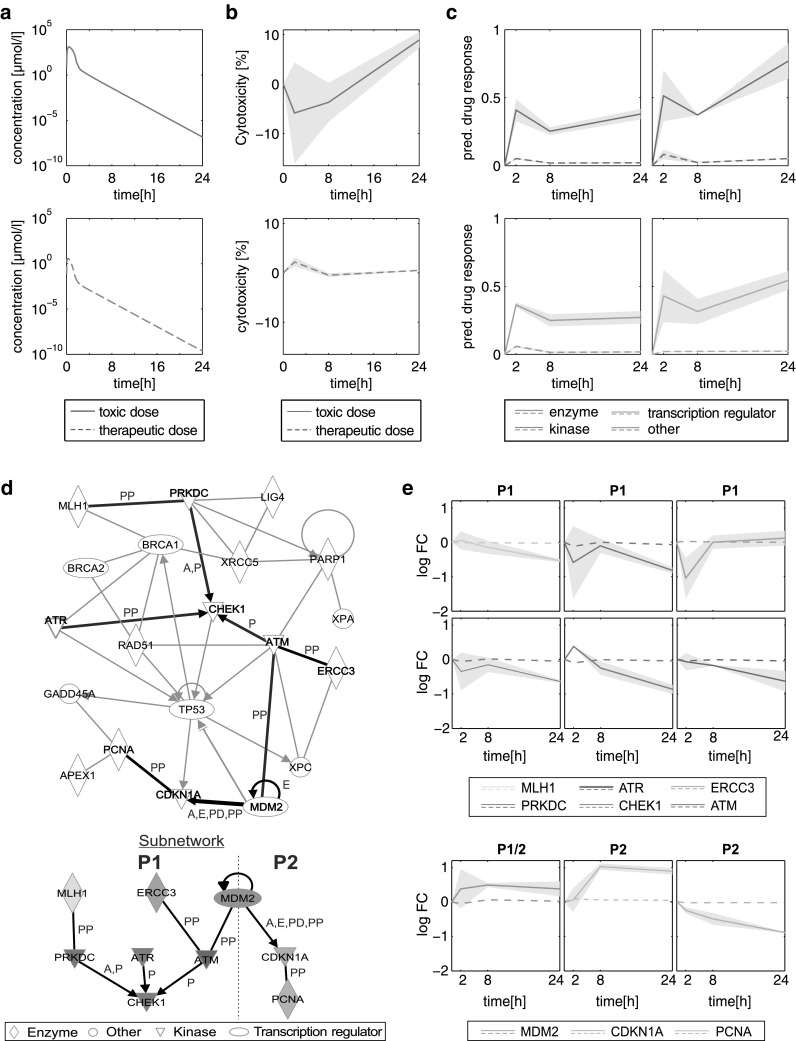
Acute liver toxicity after single dosing of azathioprine. **a** Concentration–time profiles simulated for patient 19 (Table S3) following oral administration of the toxic dose (*solid red line*) and the therapeutic dose (*dashed blue line*). **b** Cytotoxicity values over time predicted for the toxic dose (*solid red line*) and the therapeutic dose (*dashed blue line*). The Predictions were made for both replicates to represent the variability (*gray area*). The mean cytotoxicity is shown as *solid line*. **c** Predicted in vivo drug response induced by oral administration of the therapeutic dose (*dashed colored lines*) and the toxic dose (*solid colored lines*). In vivo drug responses were separated into different functional categories (enzyme, other, kinase and transcription regulator) (Table S4). The Predictions were made for both replicates to represent the variability (*gray area*). The mean drug responses are shown as *solid lines*. **d** Interaction network and processed subnetwork of genes involved in DNA damage and repair processes (Table S5). Since no expression data were available for CHEK2 and ERCC5, interactions between these genes and other were excluded. The subnetwork (*thick black lines*) was identified by considering only interactions between genes that were strongly regulated (absolute log2 fold change >0.5) in at least one time point. The interaction types (*A* activation, *E* expression, *P* phosphorylation, *PD* protein–DNA interaction, *PP* protein–protein interaction) were *highlighted* next to the specific edges. The interaction network was generated through the use of QIAGEN’s Ingenuity Pathway Analysis (IPA^®^, QIAGEN Redwood City, www.qiagen.com/ingenuity). **e** Predicted temporal expression patterns induced by the therapeutic and toxic dose were simulated for patient 19. Two critical processes (P1, P2) extracted from the subnetwork were considered separately (*dashed line* indicates separation). The first process (involved genes: MLH1, ERCC5, MDM2, PRKDC, ATR, ATM, CHEK1) resulted in the inhibition of CHEK1 that is required to initiate cell cycle arrest in response to DNA damage. The second process (involved genes: MDM2, CDKN1A, PCNA) induced the inhibition of PCNA leading to an impairment of DNA repair processes. The predictions were made for both replicates to represent the variability (*gray area*). The mean gene expressions are shown as *solid lines* (color figure online)

When regarding the various cases of acute azathioprine overdoses (Gregoriano et al. 2014), the highest overdose was observed for patient 19 (Table S3), considered in the first patient case study. Drug response in the most responsive toxicity-related pathway (DNA damage and repair; Fig. S5D) was therefore analyzed following oral administration of the specific overdose in this patient (180.1 mg/kg). Additionally, drug response was considered for the therapeutic dose (3 mg/kg) (Shapiro et al. 1993) to study changes between the toxic case and the therapeutic situation. Clinical symptoms with minor (e.g., headache) and moderate severity (e.g., dyspnoea) were observed for patient 19 (Table S3). PK profiles (Fig. 8a) as well as predicted cytotoxic response patterns (Fig. 8b) were calculated for both dose levels. In vivo drug responses for DNA damage and repair processes were separated into responses of different functional groups (Fig. 8c; Table S4). A slight increase in drug response was identified after 2 h followed by stable drug response to 24 h for nearly any functional category (enzyme, other, transcription regulator) except for kinases that were strongly affected by azathioprine overdose between 8 and 24 h (Fig. 8c). In contrast, only a slight response in all functional categories was identified when azathioprine was administered at the therapeutic dose (Fig. 8c).

Furthermore, an interaction network was generated and a subnetwork was extracted by considering all interactions between genes that were substantially perturbed (absolute log2 fold change >0.5) by azathioprine in at least one time point (Fig. 8d; Table S5). Temporal expression patterns of genes involved in two critical processes inducing DNA repair were then analyzed and compared for both dose levels (Fig. 8e). In both processes, very low changes in gene expression were identified when azathioprine was administered at the therapeutic dose contrarily to substantial changes induced by the toxic dose (Fig. 8e).

Considering the first process following acute azathioprine overdose, CHEK1 responsible for cell cycle arrest and repairing damaged DNA (Goto et al. 2012; McNeely et al. 2014; Kim et al. 2015) was activated after 2 h (Fig. 8e). Then, CHEK1 was continuously inhibited as consequence of the inhibition of kinases (ATM, ATR, PRKDC) activating CHEK1 and enzymes (MLH1, ERCC3) interacting with ATM and ATR (Fig. 8e). The impairment of adequate DNA repair after 24 h was reflected by an increased cell death measured in vitro (Fig. 8b; Igarashi et al. 2015).

In the second process (Fig. 8e), MDM2, a transcription regulator for the kinase inhibitor CDKN1A (Sánchez-Aguilera et al. 2006), was constantly activated leading to an increased activation of CDKN1A, whereas the proliferating cell nuclear antigen (PCNA) was strongly inhibited over 24 h (Fig. 8e). Since PCNA is required for DNA excision repair (Shivji et al. 1992; Essers et al. 2005), cell viability was detrimentally affected (Fig. 8b).

Analyzing both processes induced by the therapeutic dose showed very low response, which reveals no deficiency in DNA repair or cell cycle arrest (Fig. 8e). This observation was confirmed by the cell viability profile predicted for the therapeutic dose only showing slight variations compared to the control (Fig. 8b).

For this patient case study, PICD provided important insights into changes in gene expression for acute toxicity after acute azathioprine overdose at the patient level.

### Acute liver failure after multiple dosing of azathioprine: patient case study 2

**Fig. 9:**
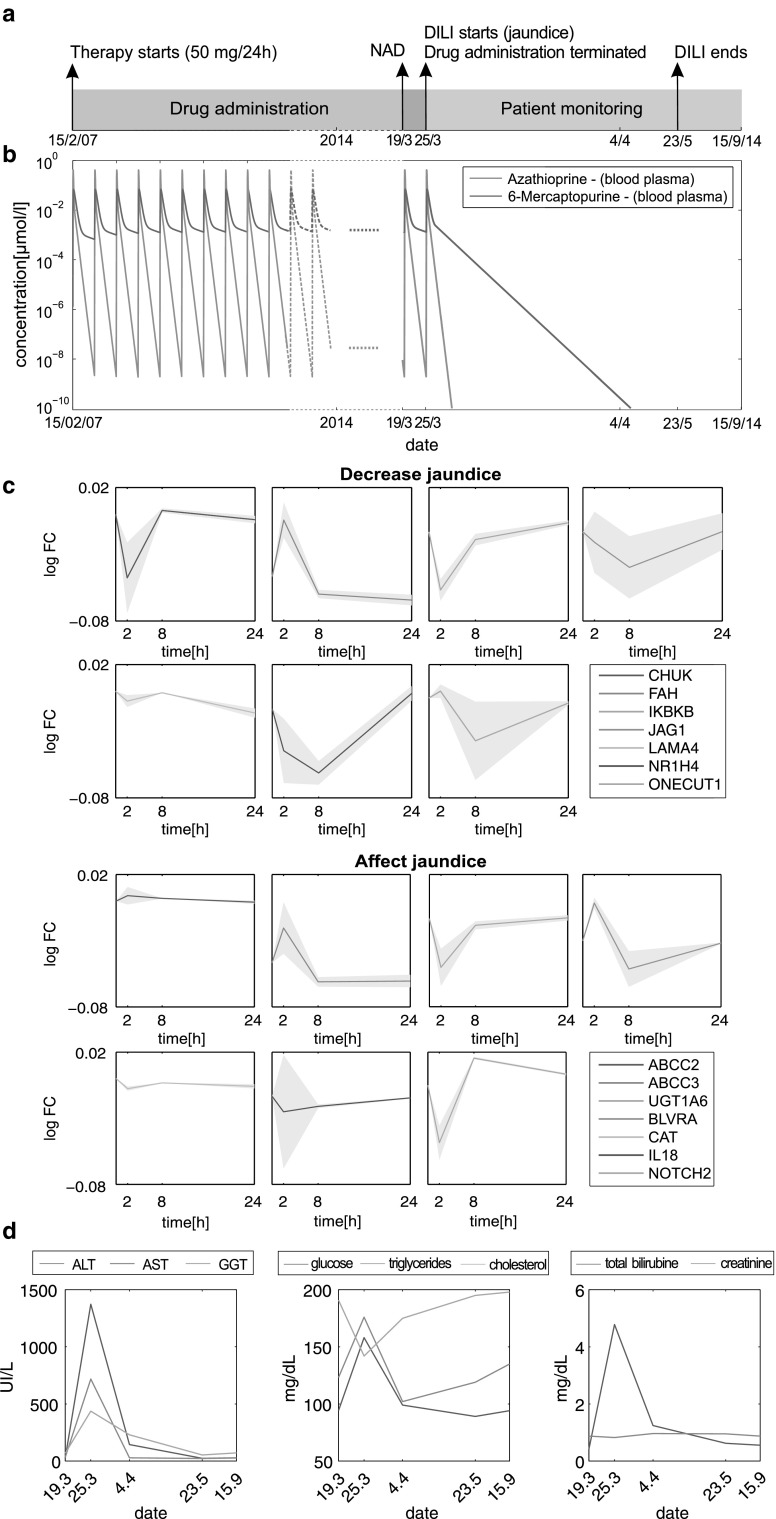
Acute liver failure after multiple dosing of azathioprine. **a** Therapy process. The 37-year-old male patient received 50 mg of azathioprine orally every day over a period of 7 years. Measurements of clinical biomarkers (e.g., ALT level) were started 1 week before DILI symptoms (jaundice) occurred. At that time, no abnormality was detected (NAD). Azathioprine treatment was terminated at the onset of liver toxicity. About 9 weeks later, jaundice disappeared. **b** Blood plasma concentrations of azathioprine (*blue line*) and 6-mercaptopurine (*red line*) were simulated for the whole therapy process following oral administration of 50 mg every 24 h. When DILI occurs, azathioprine treatment was terminated leading to a rapid clearance of both compounds within the body. **c** Expression levels of fifteen genes related to jaundice (Table S6) were exemplarily simulated over 1 day following single dosing of 50 mg of azathioprine to reflect the cellular effects at the transcriptional level induced by the permanent drug treatment (Table S6). The predictions were made for both replicates to represent the variability (*gray area*). **d** Eight different clinical biomarkers (total bilirubin, creatinine, glucose, cholesterol, triglycerides, ALT, AST and GGT) were measured at five different dates over a period of about 6 months. The first measurement was started about 1 week before DILI was observed in the specific patient (color figure online)

In contrast to acute toxicity after acute overdosing of azathioprine, in this second case study, acute liver injury was observed in the context of a chronic treatment with azathioprine at therapeutic dose by using own clinical data. A 37-year-old man with a history of thrombocytopenic purpura (TTP) was treated orally with 50 mg of azathioprine once daily over a period of 7 years (Fig. 9a). During this period, liver parameters were always within normal range. Blood plasma concentrations of azathioprine and 6-mercaptopurine were simulated for the entire evolution of the patient (Fig. 9b). The patient was seen for urgent consultation in the outpatient hepatology clinic for evaluation of new onset of jaundice and elevated liver enzymes, associated with general malaise, weakness and nausea within 5 days evolution. When jaundice has started, azathioprine treatment was terminated resulting in an instantaneous washout of the drug from the body within a few days (Fig. 9b). The diagnosis of DILI was carried out based on a scale specifically designed for DILI causality assessment, the Rousell Uctaf Causality Assessment Model (RUCAM) (Danan and Benichou 1993), with a score of 7 (probable), by ruling out other possible etiologies (viral hepatitis, excessive alcohol use, metabolic diseases, autoimmune disorders and biliary diseases).

To compare changes at the cellular level with observed clinical symptoms, temporal expression patterns following oral administration of azathioprine were predicted for fifteen genes that are associated with jaundice (Fig. 9c; Table S6). Notably, no drug accumulation occurred during multiple dosing for both azathioprine and 6-mercaptopurine since both compounds were extensively metabolized and almost completely cleared from the body within 24 h (Fig. 9b). This was also observed for the simulated drug concentrations in the intracellular space of the liver. Since additionally no in vitro response data were available for repeated dosing, drug-induced adaption due to multiple dosing was hence assumed to be negligible and the predicted gene expression profiles (Fig. 9c) were thus to be assumed to reflect the drug response at the cellular level. In addition, cell viability values predicted for the therapeutic dose disclosed no relevant elevations (Fig. S8). Investigating the response of genes affecting jaundice (Table S6) revealed no remarkable changes (Fig. 9c).

Biochemical markers measured shortly before, during and after the occurrence of jaundice indicated significant elevations (Fig. 9d). Levels of alanine transaminase (ALT) (1373 U/L), aspartate transaminase (AST) (718 U/L) and gamma-glutamyl transferase (GGT) (437 U/L) clearly exceeded clinically relevant reference levels (Ceriotti et al. 2010; Fig. 9d). Moreover, laboratory studies yielded a total bilirubin of 4.78 mg/dL reflecting a substantial increase compared to measurements before and after jaundice occurred. While concentrations of glucose and triglycerides were increased, total cholesterol (142 mg/dL) was notably diminished (Fig. 9d). The patient reported substantial improvement in his health status and liver biochemical tests a few days after the discontinuation of azathioprine, and follow-up visits after two months revealed subsequent normal laboratory tests and lack of symptoms (Fig. 9d).

In this second patient case study, PICD provided the contextualization of simulated pharmacokinetics, predicted gene expression changes induced by the therapeutic dose and in vivo measurements of biochemical markers.

## Discussion

In this study, the integrative multiscale approach PICD is presented, which allows a time-resolved description of drug-specific response profiles at the cellular level induced by in vivo drug administration at the organism level. Conceptually, PBPK models validated with blood plasma concentration–time data were used to simulate unbound drug concentrations in the interstitial space of the liver that in turn corresponds directly to the extracellular medium of in vitro experiments. Applied consistently, the systematic approach of using PBPK modeling for contextualization of in vitro toxicity data, which was exemplarily applied here for azathioprine, thus enables a generic workflow for the analysis of toxic effects of arbitrary drugs at patient level.

Predicted in vivo drug response induced by the identified doses (Fig. 6, Fig. S6) reflects the in vivo results of temporal cellular alterations observed for drug concentrations administered in vitro (Table S2). Considering oral administration (Zins et al. 1997), identified doses are in the range of toxic dose levels reported in clinical studies (Gregoriano et al. 2014) as such highlighting clinical relevance of the presented approach. Similar findings were observed when comparing the high dose (61.5 mg/kg) estimated for the rat with the minimum toxic dose determined in the in vivo study (Igarashi et al. 2015). The presented concept of coupling in vitro toxicity data with simulated interstitial concentration–time curves is based on the identification of in vivo doses that best represents the in vitro drug exposure. For this identification process, various pharmacokinetic parameters such as the maximal observed concentration (Cmax) could alternatively be considered. Here, the area under curve was selected since it represents a quantitative measure for drug exposure (Igarashi et al. 2015).

To initially validate PICD, a rat PBPK model was built (Fig. S3) by performing a cross-species extrapolation from humans to rats using the validated human PBPK model (Fig. 4). This mechanistic translation was based on recent findings (Thiel et al. 2015) and helped to compensate the unavailability of adequate PK data for the rat in the literature. Gene expression data of azathioprine-treated rats (Fig. S4C) and rat hepatocytes (Fig. S4B) together with the developed rat PBPK model were then used to assess the predictive quality of PICD by correlating predicted in vivo drug response of regulated cellular processes and biological pathways (Data S1) with findings observed in vivo (Igarashi et al. 2015). The correlation results showed high in vivo relevance of predicted in vivo drug responses in rats (Fig. 5) considering that in vitro–in vivo extrapolation is still a challenging issue (Boess et al. 2003; Heise et al. 2012; Stegeman et al. 2012; Cebola et al. 2015). Overlooking potential inter-species differences, this validation was indispensable to verify the reliability of predicted in vivo drug response for human patients since no in vivo toxicity data were available for humans. The comparison of both in vitro patterns and predicted response profiles with in vivo observations was evaluated. Correlation results obviously revealed that the extrapolation of in vitro toxicity data into an in vivo context was clearly improved by use of PICD (Fig. S5). PICD can generally be applied on any laboratory animal (e.g., rat, dog or monkey) used in the preclinical phase during drug development, since PBPK modeling allows the simulation of concentration–time profiles for several species by incorporating prior knowledge about their specific anatomy and physiology. Notably, the application of PICD on any species occurs independently, meaning that no species-specific findings were extrapolated from one species to another species.

Prediction of drug-induced cellular changes in response to interstitial PK profiles is not limited to hepatotoxicants. The compartmentalization of PBPK models enables the prediction of interstitial drug concentrations in multiple tissues or organs, for example, the heart. In this case, cellular changes obtained from compound-treated cardiomyocytes could likewise be used to get insights into adverse effects of cardiotoxic compounds in an in vivo context. Time series gene expression profiles from a toxicogenomics database (Igarashi et al. 2015) were considered here to quantify drug response over time at the cellular level. Transcriptome analysis is a powerful technique to determine changes in gene expression by measuring mRNA abundances in order to predict protein levels and activity. However, correlations between the transcriptome and proteome can be low and gene expression analysis may have limitations in elucidating stress response (Feder and Walser 2005; Haider and Pal 2013). Since in vitro data obtained by other functional -omics techniques such as proteomics or metabolomics can be analogously incorporated in the presented approach, this integrative analysis would provide a more comprehensive description of complex biological processes induced by drug administration in vivo. Likewise, in vitro toxicity data from different high-throughput technologies (Dix et al. 2007) could also be taken into account.

To demonstrate future potential of PICD in clinical application, individualized PBPK models considering specific patient physiology (Gregoriano et al. 2014) were developed to predict in vivo drug response for clinical cases of acute azathioprine overdose (Table S3). Notably, drug response of processes involved in DNA damage and repair after 1 day was highly correlated with measured cytotoxicity (Fig. 7b) indicating that changes at the transcriptional level might be directly related to cytotoxic measurements observed in vitro. High correlation determined between PSS values and corresponding drug response pointed out the relation between the drug-induced response in a critical toxicity pathway and the severity of observed clinical symptoms. Availability of additional individualized information such as patients’ genotype (Lippert et al. 2013) might be useful to further specify the translation for potential clinical applications and analysis of idiosyncratic hepatotoxicity. Genetic heterogeneity, like variants in cytochrome P450 enzymes (Dandara et al. 2011), may alter the catalytic activity of drug-related enzymes, which in turn affect drug distribution and elimination processes. For instance, genetic polymorphisms in crucial metabolic enzymes involved in the metabolism of isoniazid substantially influenced relevant pharmacokinetic processes, which may change drug efficacy at the target site or may increase the risk of toxicity (Kinzig-Schippers et al. 2005; Vuilleumier et al. 2006; Perwitasari et al. 2015). Coupling individualized PBPK models developed for different genotypes with in vitro toxicity data obtained by -omics technologies that may consider genetic diversity could therefore have a beneficial effect for individually tailored drug therapy and patient safety.

Two patient case studies have been performed to demonstrate the application of PICD on clinical cases of acute toxicity induced by different dosage regimens (Figs. 8, 9). In vivo relevance of all genes considered in both case studies was verified by assessing predicted gene expression profiles in rats. In the first patient case study, in vitro toxicity data could be directly used to simulate drug response of DNA damage and repair processes following acute azathioprine overdose (Fig. 8; Gregoriano et al. 2014). Analyzing the drug response for different functional categories identified kinases as high responsive when azathioprine was administered at the toxic dose (Fig. 8c). Further analysis of two critical processes allowed comparing drug response between toxic and therapeutic dose levels (Fig. 8d).

In the second patient case study, own data were used to study drug-induced liver failure elicited by multiple dosing of azathioprine at therapeutic dose over more than 7 years (Fig. 9a). Here, genes affecting the development of jaundice (Table S6) were specifically considered and could thus be correlated with observed clinical symptoms (Fig. 9c, d). Since PK analysis showed no drug accumulation in the therapy process, the predicted response profiles (Fig. 9c) were assumed to reflect the drug activity at the cellular level for each day. Over 24 h only low transcriptional changes induced by the therapeutic dose were predicted for jaundice-related genes. This clearly indicates that more data are needed to actually predict the sudden emergence of jaundice following long term azathioprine administration. Such data could be for instance, additional patient information or response data obtained by other functional -omics techniques such as proteomics or metabolomics. Moreover, further analyses are necessary to elucidate the molecular mechanism of the adverse reaction leading to jaundice, in particular when the toxicity was induced by chronic drug administration over a long period of time. For a mechanistic analysis, gene expression data from liver biopsies after repeated dosing would be required here to adequately investigate such toxic events. Further patient data involving among others medical history or patient lifestyle would also be necessary. Still, the application of PICD here allowed a description how cellular drug response profiles are induced by a clinically relevant dose. Thus, this patient case study provided an integrated analysis of patient-specific pharmacokinetics, drug response following oral administration of the therapeutic dose as well as the relation to several clinical biomarkers measured before, during and after the occurrence of jaundice. Finding crucial changes between predicted gene expression profiles for therapeutic and toxic dose levels could thus enhance the identification of useful biomarkers in patients and subsequently lead to an early detection of potential toxicity.

Clearly, the in vivo predictions in the rat are not fully accurate and the application of PICD inhibits some inherent level of uncertainty. However, it should be noted that the approach presented provides a generic workflow for quantitative analyses of in vitro measurements within an in vivo context. The PBPK models at the organism level were carefully qualified by validating the model with clinical data for different doses and different administration routes. Furthermore, the expression data at the cellular scale were taken from TG-GATEs (Igarashi et al. 2015), which is one of the most systematic and best curated toxicological databases in the world. Hence, despite some inherent yet inevitable uncertainty in the input, the predictions made by PICD represent nevertheless a sound extrapolation of in vitro data to an in vivo environment. Please note also that PICD allows an animal-free assessment of drug-induced toxicity which is fully in line with 3*R* principles. Assuming that appropriate in vitro toxicity tests were concluded, using PICD for laboratory animals may improve the predictability of toxic events in an in vivo context and may facilitate the identification of a safe dose. The demand for animal kill is therefore reduced since PICD is an in silico based approach.

PICD allows describing temporal changes at the cellular level induced by drug administration in vivo and hence provides a generic platform to contextualize in vitro measurements of different -omics studies at the organism level. Therefore, changes in cellular events induced by clinically relevant or toxic dose levels can be predicted for humans and thus might facilitate the investigation of in vitro findings within a patient context for clinical applications in the future.

## Materials and methods

### Prediction of in vivo response in humans and rats

PICD was applied on rats and humans to quantify in vivo responses for different time points and dose levels. Gene expression values (log2 fold change) and cell viability values both measured in vitro (Igarashi et al. 2015) as well as gene response values, defined as absolute log2 fold change, were mapped to the nine identified in vivo doses (Table S2) and were linearly interpolated to determine respective dose–response profiles for the different time points (2, 8 and 24 h) (Igarashi et al. 2015). Note that the identification of the in vivo doses is dependent on the underlying PBPK model and the specific dosage regimen. Time-resolved in vivo response profiles were then predicted for arbitrary doses by assigning gene expression, cytotoxicity or gene response values after 2, 8 and 24 h. In vivo drug responses of all terms (GO) and pathways (KEGG, TOX) (Table S1) that were significantly overrepresented in at least one treatment (e.g., middle—2 h; Data S1) were predicted by computing the mean gene response level of all genes assigned to a specific term or pathway. Significant increase in drug response values after the early time point was evaluated by one-sample *t* test, while changes between individual time points were assessed by using one-way ANOVA followed by Tukey–Kramer multiple comparison test.

### Validation of predicted in vivo profiles in rats

To validate PICD, predicted in vivo drug responses were linearly interpolated to perform a correlation with time-matched drug response observed in the in vivo study (Igarashi et al. 2015). All cellular processes or biological pathways that were significantly affected in rats for at least one treatment (Data S1) were considered for this correlation. Predicted expression profiles for all genes considered in the two case studies were analogously validated with in vivo gene expression profiles observed in rats (Igarashi et al. 2015). All correlation analyses were performed by calculating Pearson’s correlation coefficient r and the corresponding p-value p.

### PBPK model development

In the PBPK model structure, compound-specific properties and physiological parameters of the organism such as organ volumes can be considered independently (Fig. S9). The latter parameters describing the physiology and anatomy of the organism are provided by the PBPK modeling software (Willmann et al. 2003) (Supplementary Materials). Besides physiochemical properties such as the lipophilicity or plasma protein binding values influencing in particular drug disposition in absorption and distribution processes, active drug transport or metabolizing reactions were integrated to describe the drug clearance in the body. The Michaelis–Menten constant (*K*_M_) and the maximum velocity (*v*_max_) were used to characterize the kinetic behavior of such active processes. Abundances of relevant enzymes and transporters in multiple compartments were quantified by using tissue-specific gene expression data (Meyer et al. 2012).

The first step in PBPK model development is model identification and parameter optimization by comparing simulated concentration–time profiles with measured PK data (Fig. S9). Once sufficient model accuracy is reached, quantified in general by visual inspection, model validity can be confirmed by extrapolating the initial reference PBPK model to different dosage regimens or different patient populations. Note that all model parameters of the reference PBPK model were left unchanged for the validation step, except anthropometric parameters characterizing the specific patient subgroup. In this study, the quality of the developed PBPK model of azathioprine was assessed by comparing simulated PK data with different experimental concentration–time profiles from the literature (Odlind et al. 1986; Van Os et al. 1996; Zins et al. 1997). To indicate the model quality, PBPK models were evaluated by calculating a root-mean-square deviation (RMSD) whereby the differences of measured and simulated concentrations were normalized by respective experimental values (Thiel et al. 2015). Moreover, a linear regression was performed for simulated and observed concentrations. Coefficient of determination *R* ^2^ as well as the slope a and the intercept b of the linear equation was then additionally used to evaluate the ‘goodness of fit.’

### Analysis of in vitro toxicity data

Raw data were downloaded from TG-GATEs (Igarashi et al. 2015) (Supplementary Materials). Gene expression profiles measured with Affymetrix Human Genome U133 Plus 2.0 and Affymetrix Rat Genome 230 2.0 GeneChip arrays were normalized by applying the GC-RMA method (Wu et al. 2004). Probe sets on the chip were mapped to Entrez Gene IDs using BrainArray custom CDF files (version 19.0.0, ENTREZG) (Dai et al. 2005). For each treatment, differential gene expression analysis was performed by linear models using limma (Smyth 2004). Compound-treated hepatocytes exposed to different concentrations were therefore compared to their respective time-matched controls. *p* values were adjusted by Benjamini–Hochberg correction for multiple testing (Benjamini and Hochberg 1995). Fold change values were calculated to indicate gene expression changes compared to the time-matched controls. Gene expression profiles of primary human and rat hepatocytes were further analyzed by applying hypergeometric testing (Falcon and Gentleman 2007) on each subset of differentially expressed genes identified for each treatment to determine significantly overrepresented terms (GO) and pathways (KEGG, TOX) (Data S1). *p* values were adjusted by Benjamini–Hochberg correction. Terms and pathways with a size of assigned genes lower than five were filtered out. To investigate only GO terms with a high level of specialization, an additional filtering step was performed on significant results (Supplementary Materials, Data S3).

### Clinical cases of acute toxicity after single dosing of azathioprine

PICD was used for different clinical cases of acute azathioprine overdose reported between 1995 and 2013 (Gregoriano et al. 2014). Patients showing symptoms most likely caused by other drugs than azathioprine (Gregoriano et al. 2014) were not taken into account. Moreover, only patients for whom no decontamination measures were undertaken after exposure to a single oral overdose were considered leading to a total of eight clinical cases (Table S3). Consequently, eight PBPK models were developed incorporating individual anthropometric parameters (age, sex and weight). PICD was then applied on each patient, thereby simulating drug concentration–time profiles in the interstitial space of the liver following oral administration of the specific overdose. In a next step, drug response in the most responsive toxicity-related pathway (DNA damage and repair) (Fig. S5D) and cytotoxicity values were predicted at every time point. Finally, in vivo drug responses were correlated with global cytotoxic observations by calculating Pearson’s correlation coefficient *r*, while PSS values were correlated with drug response values after 1 day by calculating Spearman’s rank correlation coefficient rho. In the latter correlation analysis, patient 17 was not considered, since she remained asymptomatic after a heavy overdose of azathioprine (Table S3). Furthermore, patient 19 was considered in a patient case study, thereby investigating acute toxicity after single dosing of azathioprine.

## Electronic supplementary material

Below is the link to the electronic supplementary material.

Data S1 **Enriched terms and pathways**. Significantly overrepresented terms (GO) and pathways (KEGG, TOX) identified for human and rat hepatocytes as well as correspondent p-values. The list of GO terms presents all terms except those removed by the filtering method (PDF 2465 kb)

Data S2 **Correlation results for significantly regulated pathways and cellular processes in rats**. Correlation analysis was applied between predicted drug response profiles and in vivo measurements in rats for significantly regulated pathways and cellular processes. Correlation analyses were performed by calculating Pearson’s correlation coefficient r and the corresponding p-value p (PDF 283 kb)

Data S3 **Deleted GO terms**. Go terms for the three sub-ontologies (BP, CC, and MF) that have been deleted after applying the presented filtering method of GO terms (Supplementary Materials) (PDF 2417 kb)

Supplementary Materials (DOCX 37 kb)

Figure S1 **Schematic representation of a multiscale whole-body PBPK model**. Schematic representation of a multiscale whole-body PBPK model including 15 different tissues and organs that are connected by blood flow. Sub-compartmentalization is exemplarily presented for a default compartment (PNG 1415 kb)

Figure S2 **PBPK model assessment**. Simulated concentration-time profiles were compared to experimental data. Observed vs. predicted plots including RMSD value, coefficient of determination (R^2^), and the equation of the linear regression were generated for the reference and validated PBPK model. Simulated concentration-time profiles were compared to experimental data. Observed vs. predicted plots including RMSD value, coefficient of determination (R^2^), and the equation of the linear regression were generated for the reference and validated PBPK model (PNG 1248 kb)

Figure S3 **Rat PBPK model**. Blood plasma concentration-time profiles of azathioprine and 6-mercaptopurine were simulated for rats (dashed blue line, dashed green line) and for humans (solid blue line, solid green line) after oral administration of 100 mg of azathioprine. The rat PBPK model of azathioprine was developed by considering rat-specific anatomy and physiology in the human PBPK model according to (Thiel et al. 2015) (PNG 212 kb)

Figure S4 **Azathioprine-induced in vitro and in vivo gene expression data**. Heatmaps of in vitro and in vivo expression data of genes that were differentially expressed in at least one treatment of the specific experiment (Igarashi et al. 2015). Three different exposure levels of azathioprine were administered (low (green), middle (orange), high (red)) and gene expression was measured after three and four different exposure durations in the in vitro and in vivo case, respectively. The number below each column indicates the number of differentially expressed genes identified in the specific treatment. Gene expression values in each row were z-score normalized. (**A**) In vitro gene expression data obtained in primary human hepatocytes. (**B**) In vitro gene expression data obtained in primary rat hepatocytes. (**C**) In vivo gene expression data obtained in rats (PNG 446 kb)

Figure S5 **Correlation between observed in vivo drug response and both predicted in vivo drug response and observed in vitro drug response**. Predicted in vivo drug response (blue) induced by the identified toxic dose (Igarashi et al. 2015) as well as corresponding in vitro profiles (red) induced by the toxic concentration (Igarashi et al. 2015) were correlated with measurements observed in vivo (Igarashi et al. 2015). All cellular processes or biological pathways that were significantly regulated in at least one treatment (Data S1) and all genes analyzed in both case studies (Table S4, Table S6) were considered for the correlation of drug response and gene expression, respectively. Correlation analyses were performed by calculating Pearson’s correlation coefficient r and the corresponding p-value p. (**A**) Correlation of significantly affected KEGG pathways. (**B**) Correlation of significantly affected toxicity-related pathways. (**C**) Correlation of significantly affected biological processes. (**D**) Correlation of significantly affected cellular components. (**E**) Correlation of significantly affected molecular functions. (**F**) Correlation of genes considered in both case studies (PNG 1750 kb)

Figure S6 **Predicted in vivo drug response**. In vivo drug responses of significantly affected GO terms and toxicity-related pathways (Benjamini-Hochberg corrected p < 0.01) following in vivo drug administration of d_low_, d_middle_, and d_high_. The color scale depicts predicted in vivo drug responses. (**A**) Go terms of biological processes. (**B**) Go terms of cellular components. (**C**) Go terms of molecular functions. (**D**) Toxicity-related pathways (PNG 725 kb)

Figure S7 **Predicted in vivo drug response of DNA replication**. (**A**) Drug response map exemplarily shown for DNA replication reflecting time- and dose-dependent effects following administration of azathioprine at dose levels d_low_, d_middle_ and d_high_ (black lines). The color scale depicts predicted in vivo drug responses. (**B**) Predicted in vivo drug response over time induced by doses d_low_, d_middle_ and d_high_ (PNG 147 kb)

Figure S8 **Predicted in vivo cytotoxicity over time**. In vivo cytotoxicity values over time induced by the therapeutic dose were predicted for both replicates (gray area). The mean cytotoxicity values are shown as solid line (PNG 18 kb)

Figure S9 **Workflow for PBPK model development and validation**. After parametrizing compound-specific properties and organism-specific parameters in the reference PBPK model, the model quality is evaluated by comparing simulated drug concentrations with experimental data from literature. If a sufficient model accuracy has been reached, a subsequent validation step enables reliable model extrapolations. Amongst others, this validation step ensures accurate predictions of concentration-time profiles in various compartments. Otherwise, the PBPK model is revised in a refinement step thereby adjusting key model parameters or adding more active transport processes or metabolizing reactions to improve the description of physiological processes governing the fate of the considered compound within the body (PNG 184 kb)

Figure S10 **Filtering gene ontology terms**. The presented graph illustrates an exemplary subgraph of the complete GO graph. In total, four biological processes were identified as significantly enriched (blue and green nodes). After applying the filtering procedure, three terms were filtered out (blue nodes) while one remained for further analysis (green). Note that the green node represents the highest specialization (PNG 754 kb)

Table S1 **Toxicity-related biological pathways**. Symbols as well as human and rat Entrez IDs for 370 genes showing high response to toxic compounds were grouped in thirteen different biological pathways. The genes and the functional gene grouping terms were taken from the Human Molecular Toxicology PathwayFinder RT^2^ Profiler^TM^ PCR Array (SABiosciences, http://www.sabiosciences.com). Rat Entrez IDs were identified through the use of QIAGEN’s Ingenuity Pathway Analysis (IPA^®^, QIAGEN Redwood City, www.qiagen.com/ingenuity) (DOCX 41 kb)

Table S2 **Dose identification**. In vitro concentration, exposure duration, resulting in vitro exposure, and identified in vivo dose for the specific treatments for rats and humans (DOCX 26 kb)

Table S3 **Clinical cases of acute azathioprine overdose**. Anthropometric parameters (age, weight, and sex), administered dose, and observed symptoms including assigned Poisoning Severity Scores (PSS) (Persson et al. 1998). The clinical data were taken from (Gregoriano et al. 2014) (DOCX 28 kb)

Table S4 **Genes involved in the DNA damage & repair pathway**. Symbols, Entrez gene name, type, as well as human and rat Entrez identifier for all genes involved in the DNA damage & repair pathway. Functional classifications were taken from QIAGEN’s Ingenuity Pathway Analysis (IPA^®^, QIAGEN Redwood City, www.qiagen.com/ingenuity) (DOCX 27 kb)

Table S5 **Interaction network**. Interactions between genes involved in DNA damage and repair processes. The interactions were identified through the use of QIAGEN’s Ingenuity Pathway Analysis (IPA^®^, QIAGEN Redwood City, www.qiagen.com/ingenuity) (DOCX 26 kb)

Table S6 **Genes related to jaundice**. Symbol, Entrez gene name, type, human and rat Entrez identifier and assigned relation for all genes associated with jaundice. Functional classifications and assigned relations on jaundice were taken from QIAGEN’s Ingenuity Pathway Analysis (IPA^®^, QIAGEN Redwood City, www.qiagen.com/ingenuity) (DOCX 26 kb)
