# Supplementary material for: Model-based contextualization of in vitro toxicity data quantitatively predicts in vivo drug response in patients
Source: Arch Toxicol. 2016 May 9;91(2):865–83. doi: 10.1007/s00204-016-1723-x (PMC5306109; doi:10.1007/s00204-016-1723-x)
Supplement: Supplementary file 1 — Data S1 Enriched terms and pathways. Significantly overrepresented terms (GO) and pathways (KEGG, TOX) identified for human and rat hepatocytes as well as correspondent p-values. The list of GO terms presents all terms except those removed by the filtering method (PDF 2465 kb) [file 204_2016_1723_MOESM1_ESM.pdf]

| Species | Category | Term ID | Term name | Treatment | P-value |
|---|---|---|---|---|---|
| Human | KEGG | hsa00240 | Pyrimidine metabolism | Low - 24 h | 0.0001855 |
| Human | KEGG | hsa00982 | Drug metabolism - cytochrome P450 | Low - 24 h | 0.00018996 |
| Human | KEGG | hsa03030 | DNA replication | Low - 24 h | 1.10E-07 |
| Human | KEGG | hsa04110 | Cell cycle | Low - 24 h | 1.25E-16 |
| Human | KEGG | hsa04114 | Oocyte meiosis | Low - 24 h | 1.44E-05 |
| Human | KEGG | hsa04115 | p53 signaling pathway | Low - 24 h | 4.98E-07 |
| Human | KEGG | hsa00830 | Retinol metabolism | Middle - 24 h | 3.84E-05 |
| Human | KEGG | hsa03030 | DNA replication | Middle - 24 h | 1.59E-12 |
| Human | KEGG | hsa03420 | Nucleotide excision repair | Middle - 24 h | 5.47E-05 |
| Human | KEGG | hsa03430 | Mismatch repair | Middle - 24 h | 4.99E-07 |
| Human | KEGG | hsa03460 | Fanconi anemia pathway | Middle - 24 h | 2.99E-05 |
| Human | KEGG | hsa04110 | Cell cycle | Middle - 24 h | 2.62E-17 |
| Human | KEGG | hsa04115 | p53 signaling pathway | Middle - 24 h | 4.83E-07 |
| Human | KEGG | hsa04141 | Protein processing in endoplasmic reticulum | Middle - 24 h | 1.41E-05 |
| Human | KEGG | hsa05202 | Transcriptional misregulation in cancer | Middle - 24 h | 0.00011211 |
| Human | KEGG | hsa04068 | FoxO signaling pathway | High - 8 h | 8.96E-07 |
| Human | KEGG | hsa04110 | Cell cycle | High - 8 h | 1.87E-06 |
| Human | KEGG | hsa04115 | p53 signaling pathway | High - 8 h | 3.66E-05 |
| Human | KEGG | hsa00240 | Pyrimidine metabolism | High - 24 h | 0.00012092 |
| Human | KEGG | hsa03030 | DNA replication | High - 24 h | 2.92E-12 |
| Human | KEGG | hsa03430 | Mismatch repair | High - 24 h | 2.98E-05 |
| Human | KEGG | hsa03460 | Fanconi anemia pathway | High - 24 h | 4.33E-05 |
| Human | KEGG | hsa04110 | Cell cycle | High - 24 h | 2.43E-15 |
| Human | KEGG | hsa04115 | p53 signaling pathway | High - 24 h | 3.20E-05 |
| Human | KEGG | hsa04141 | Protein processing in endoplasmic reticulum | High - 24 h | 1.06E-08 |
| Human | TOX | TOX:04 | DNA Damage & Repair | Low - 24 h | 4.86E-06 |
| Human | TOX | TOX:08 | Immunotoxicity | Low - 24 h | 0.00026011 |
| Human | TOX | TOX:02 | Cholestasis | Middle - 8 h | 0.0032688 |
| Human | TOX | TOX:04 | DNA Damage & Repair | Middle - 24 h | 4.45E-09 |
| Human | TOX | TOX:08 | Immunotoxicity | Middle - 24 h | 0.00087295 |
| Human | TOX | TOX:11 | Oxidative Stress & Antioxidant Response | Middle - 24 h | 0.0016711 |
| Human | TOX | TOX:07 | Heat Shock Response | High - 8 h | 8.55E-05 |
| Human | TOX | TOX:04 | DNA Damage & Repair | High - 24 h | 5.10E-07 |
| Human | TOX | TOX:05 | ER Stress & Unfolded Protein Response | High - 24 h | 0.00062041 |
| Human | TOX | TOX:07 | Heat Shock Response | High - 24 h | 0.00031518 |
| Human | TOX | TOX:08 | Immunotoxicity | High - 24 h | 0.0038211 |
| Human | TOX | TOX:11 | Oxidative Stress & Antioxidant Response | High - 24 h | 0.0049039 |
| Human | TOX | TOX:12 | Phospholipidosis | High - 24 h | 0.0014279 |
| Human | GO (BP) | GO:0007091 | metaphase/anaphase transition of mitotic cell cycle | Low - 24 h | 1.16E-10 |

| Species | Category | Term ID | Term name | Treatment | P-value |
|---|---|---|---|---|---|
| Human | GO (BP) | GO:0006271 | DNA strand elongation involved in DNA replication | Low - 24 h | 1.42E-09 |
| Human | GO (BP) | GO:0034080 | CENP-A containing nucleosome assembly | Low - 24 h | 6.04E-08 |
| Human | GO (BP) | GO:0051988 | regulation of attachment of spindle microtubules to kinetochore | Low - 24 h | 9.93E-08 |
| Human | GO (BP) | GO:0006206 | pyrimidine nucleobase metabolic process | Low - 24 h | 1.80E-07 |
| Human | GO (BP) | GO:0007076 | mitotic chromosome condensation | Low - 24 h | 7.54E-07 |
| Human | GO (BP) | GO:0007094 | mitotic spindle assembly checkpoint | Low - 24 h | 8.99E-07 |
| Human | GO (BP) | GO:0008608 | attachment of spindle microtubules to kinetochore | Low - 24 h | 1.20E-06 |
| Human | GO (BP) | GO:0000086 | G2/M transition of mitotic cell cycle | Low - 24 h | 1.48E-06 |
| Human | GO (BP) | GO:0042493 | response to drug | Low - 24 h | 2.09E-06 |
| Human | GO (BP) | GO:0045132 | meiotic chromosome segregation | Low - 24 h | 2.42E-06 |
| Human | GO (BP) | GO:0006084 | acetyl-CoA metabolic process | Low - 24 h | 1.33E-05 |
| Human | GO (BP) | GO:0007100 | mitotic centrosome separation | Low - 24 h | 2.14E-05 |
| Human | GO (BP) | GO:0008283 | cell proliferation | Low - 24 h | 3.28E-05 |
| Human | GO (BP) | GO:0055114 | oxidation-reduction process | Low - 24 h | 3.46E-05 |
| Human | GO (BP) | GO:0006200 | ATP catabolic process | Low - 24 h | 3.64E-05 |
| Human | GO (BP) | GO:1901606 | alpha-amino acid catabolic process | Low - 24 h | 4.91E-05 |
| Human | GO (BP) | GO:0007131 | reciprocal meiotic recombination | Low - 24 h | 5.97E-05 |
| Human | GO (BP) | GO:0009411 | response to UV | Low - 24 h | 7.00E-05 |
| Human | GO (BP) | GO:0007018 | microtubule-based movement | Low - 24 h | 7.17E-05 |
| Human | GO (BP) | GO:0015949 | nucleobase-containing small molecule interconversion | Low - 24 h | 8.60E-05 |
| Human | GO (BP) | GO:0009636 | response to toxic substance | Low - 24 h | 9.46E-05 |
| Human | GO (BP) | GO:0031570 | DNA integrity checkpoint | Low - 24 h | 0.00011555 |
| Human | GO (BP) | GO:0090399 | replicative senescence | Low - 24 h | 0.00011972 |
| Human | GO (BP) | GO:0000910 | cytokinesis | Low - 24 h | 0.00016247 |
| Human | GO (BP) | GO:0006270 | DNA replication initiation | Low - 24 h | 0.00017279 |
| Human | GO (BP) | GO:0009437 | carnitine metabolic process | Low - 24 h | 0.0001839 |
| Human | GO (BP) | GO:0051382 | kinetochore assembly | Low - 24 h | 0.00026965 |
| Human | GO (BP) | GO:0090068 | positive regulation of cell cycle process | Low - 24 h | 0.00031613 |
| Human | GO (BP) | GO:0006366 | transcription from RNA polymerase II promoter | Middle - 8 h | 7.37E-07 |
| Human | GO (BP) | GO:0031016 | pancreas development | Middle - 8 h | 4.88E-06 |
| Human | GO (BP) | GO:0060412 | ventricular septum morphogenesis | Middle - 8 h | 1.59E-05 |
| Human | GO (BP) | GO:0006271 | DNA strand elongation involved in DNA replication | Middle - 24 h | 4.43E-13 |
| Human | GO (BP) | GO:0034080 | CENP-A containing nucleosome assembly | Middle - 24 h | 6.75E-12 |
| Human | GO (BP) | GO:0007091 | metaphase/anaphase transition of mitotic cell cycle | Middle - 24 h | 5.31E-09 |
| Human | GO (BP) | GO:0008283 | cell proliferation | Middle - 24 h | 2.30E-08 |
| Human | GO (BP) | GO:0007076 | mitotic chromosome condensation | Middle - 24 h | 9.58E-08 |
| Human | GO (BP) | GO:0006987 | activation of signaling protein activity involved in unfolded protein response | Middle - 24 h | 2.03E-07 |
| Human | GO (BP) | GO:0000083 | regulation of transcription involved in G1/S transition of mitotic cell cycle | Middle - 24 h | 2.82E-07 |
| Human | GO (BP) | GO:0006270 | DNA replication initiation | Middle - 24 h | 3.35E-07 |

| Species | Category | Term ID | Term name | Treatment | P-value |
|---|---|---|---|---|---|
| Human | GO (BP) | GO:0008608 | attachment of spindle microtubules to kinetochore | Middle - 24 h | 8.49E-07 |
| Human | GO (BP) | GO:0031100 | organ regeneration | Middle - 24 h | 9.09E-07 |
| Human | GO (BP) | GO:0032201 | telomere maintenance via semi-conservative replication | Middle - 24 h | 3.24E-06 |
| Human | GO (BP) | GO:0007094 | mitotic spindle assembly checkpoint | Middle - 24 h | 4.88E-06 |
| Human | GO (BP) | GO:0051988 | regulation of attachment of spindle microtubules to kinetochore | Middle - 24 h | 5.33E-06 |
| Human | GO (BP) | GO:0000722 | telomere maintenance via recombination | Middle - 24 h | 7.00E-06 |
| Human | GO (BP) | GO:0007080 | mitotic metaphase plate congression | Middle - 24 h | 7.44E-06 |
| Human | GO (BP) | GO:0000910 | cytokinesis | Middle - 24 h | 1.10E-05 |
| Human | GO (BP) | GO:0070365 | hepatocyte differentiation | Middle - 24 h | 1.12E-05 |
| Human | GO (BP) | GO:0051382 | kinetochore assembly | Middle - 24 h | 2.13E-05 |
| Human | GO (BP) | GO:0045132 | meiotic chromosome segregation | Middle - 24 h | 2.20E-05 |
| Human | GO (BP) | GO:0007019 | microtubule depolymerization | Middle - 24 h | 2.61E-05 |
| Human | GO (BP) | GO:0014070 | response to organic cyclic compound | Middle - 24 h | 2.63E-05 |
| Human | GO (BP) | GO:0006302 | double-strand break repair | Middle - 24 h | 4.12E-05 |
| Human | GO (BP) | GO:0006206 | pyrimidine nucleobase metabolic process | Middle - 24 h | 4.63E-05 |
| Human | GO (BP) | GO:0006297 | nucleotide-excision repair, DNA gap filling | Middle - 24 h | 4.77E-05 |
| Human | GO (BP) | GO:0046394 | carboxylic acid biosynthetic process | Middle - 24 h | 4.80E-05 |
| Human | GO (BP) | GO:0007584 | response to nutrient | Middle - 24 h | 6.01E-05 |
| Human | GO (BP) | GO:0000086 | G2/M transition of mitotic cell cycle | Middle - 24 h | 6.59E-05 |
| Human | GO (BP) | GO:0090307 | spindle assembly involved in mitosis | Middle - 24 h | 9.96E-05 |
| Human | GO (BP) | GO:0006283 | transcription-coupled nucleotide-excision repair | Middle - 24 h | 0.0001042 |
| Human | GO (BP) | GO:0016572 | histone phosphorylation | Middle - 24 h | 0.00010875 |
| Human | GO (BP) | GO:0006268 | DNA unwinding involved in DNA replication | Middle - 24 h | 0.00010983 |
| Human | GO (BP) | GO:0009411 | response to UV | Middle - 24 h | 0.00013072 |
| Human | GO (BP) | GO:0071281 | cellular response to iron ion | Middle - 24 h | 0.00013533 |
| Human | GO (BP) | GO:1901606 | alpha-amino acid catabolic process | Middle - 24 h | 0.00013547 |
| Human | GO (BP) | GO:0006084 | acetyl-CoA metabolic process | Middle - 24 h | 0.00014285 |
| Human | GO (BP) | GO:0006200 | ATP catabolic process | Middle - 24 h | 0.00014961 |
| Human | GO (BP) | GO:0071479 | cellular response to ionizing radiation | Middle - 24 h | 0.00015656 |
| Human | GO (BP) | GO:0009200 | deoxyribonucleoside triphosphate metabolic process | Middle - 24 h | 0.00022348 |
| Human | GO (BP) | GO:0015949 | nucleobase-containing small molecule interconversion | Middle - 24 h | 0.00022348 |
| Human | GO (BP) | GO:0008630 | intrinsic apoptotic signaling pathway in response to DNA damage | Middle - 24 h | 0.00024137 |
| Human | GO (BP) | GO:0043065 | positive regulation of apoptotic process | Middle - 24 h | 0.00029346 |
| Human | GO (BP) | GO:0010332 | response to gamma radiation | Middle - 24 h | 0.00030114 |
| Human | GO (BP) | GO:0007100 | mitotic centrosome separation | Middle - 24 h | 0.00030171 |
| Human | GO (BP) | GO:0051255 | spindle midzone assembly | Middle - 24 h | 0.00030171 |
| Human | GO (BP) | GO:0060236 | regulation of mitotic spindle organization | Middle - 24 h | 0.00030171 |
| Human | GO (BP) | GO:0045765 | regulation of angiogenesis | Middle - 24 h | 0.00030212 |
| Human | GO (BP) | GO:0040001 | establishment of mitotic spindle localization | Middle - 24 h | 0.00031938 |

| Species | Category | Term ID | Term name | Treatment | P-value |
|---|---|---|---|---|---|
| Human | GO (BP) | GO:0070301 | cellular response to hydrogen peroxide | Middle - 24 h | 0.00036094 |
| Human | GO (BP) | GO:0000725 | recombinational repair | Middle - 24 h | 0.000415 |
| Human | GO (BP) | GO:0008283 | cell proliferation | High - 8 h | 1.78E-06 |
| Human | GO (BP) | GO:0006366 | transcription from RNA polymerase II promoter | High - 8 h | 3.23E-06 |
| Human | GO (BP) | GO:0033993 | response to lipid | High - 8 h | 7.54E-06 |
| Human | GO (BP) | GO:0045736 | negative regulation of cyclin-dependent protein serine/threonine kinase activity | High - 8 h | 7.98E-06 |
| Human | GO (BP) | GO:0014070 | response to organic cyclic compound | High - 8 h | 1.96E-05 |
| Human | GO (BP) | GO:0009725 | response to hormone | High - 8 h | 7.34E-05 |
| Human | GO (BP) | GO:0031016 | pancreas development | High - 8 h | 7.59E-05 |
| Human | GO (BP) | GO:0045944 | positive regulation of transcription from RNA polymerase II promoter | High - 8 h | 7.98E-05 |
| Human | GO (BP) | GO:0060412 | ventricular septum morphogenesis | High - 8 h | 9.00E-05 |
| Human | GO (BP) | GO:0090009 | primitive streak formation | High - 8 h | 0.00010067 |
| Human | GO (BP) | GO:0008285 | negative regulation of cell proliferation | High - 8 h | 0.00010913 |
| Human | GO (BP) | GO:0048872 | homeostasis of number of cells | High - 8 h | 0.00012165 |
| Human | GO (BP) | GO:0060537 | muscle tissue development | High - 8 h | 0.00019426 |
| Human | GO (BP) | GO:0048661 | positive regulation of smooth muscle cell proliferation | High - 8 h | 0.00019805 |
| Human | GO (BP) | GO:0070647 | protein modification by small protein conjugation or removal | High - 8 h | 0.0002139 |
| Human | GO (BP) | GO:0055088 | lipid homeostasis | High - 8 h | 0.0002197 |
| Human | GO (BP) | GO:0030518 | intracellular steroid hormone receptor signaling pathway | High - 8 h | 0.00026118 |
| Human | GO (BP) | GO:0070848 | response to growth factor | High - 8 h | 0.00026479 |
| Human | GO (BP) | GO:0006271 | DNA strand elongation involved in DNA replication | High - 24 h | 1.12E-12 |
| Human | GO (BP) | GO:0034080 | CENP-A containing nucleosome assembly | High - 24 h | 1.12E-10 |
| Human | GO (BP) | GO:0006987 | activation of signaling protein activity involved in unfolded protein response | High - 24 h | 2.42E-08 |
| Human | GO (BP) | GO:0006270 | DNA replication initiation | High - 24 h | 3.57E-07 |
| Human | GO (BP) | GO:0007076 | mitotic chromosome condensation | High - 24 h | 5.39E-07 |
| Human | GO (BP) | GO:0007091 | metaphase/anaphase transition of mitotic cell cycle | High - 24 h | 7.41E-07 |
| Human | GO (BP) | GO:0000086 | G2/M transition of mitotic cell cycle | High - 24 h | 1.86E-06 |
| Human | GO (BP) | GO:0000083 | regulation of transcription involved in G1/S transition of mitotic cell cycle | High - 24 h | 2.21E-06 |
| Human | GO (BP) | GO:0032201 | telomere maintenance via semi-conservative replication | High - 24 h | 2.21E-06 |
| Human | GO (BP) | GO:1901606 | alpha-amino acid catabolic process | High - 24 h | 3.21E-06 |
| Human | GO (BP) | GO:0000722 | telomere maintenance via recombination | High - 24 h | 5.25E-06 |
| Human | GO (BP) | GO:0008608 | attachment of spindle microtubules to kinetochore | High - 24 h | 5.45E-06 |
| Human | GO (BP) | GO:0015949 | nucleobase-containing small molecule interconversion | High - 24 h | 7.90E-06 |
| Human | GO (BP) | GO:0007584 | response to nutrient | High - 24 h | 1.09E-05 |
| Human | GO (BP) | GO:0040001 | establishment of mitotic spindle localization | High - 24 h | 1.33E-05 |
| Human | GO (BP) | GO:0042493 | response to drug | High - 24 h | 1.84E-05 |
| Human | GO (BP) | GO:0051988 | regulation of attachment of spindle microtubules to kinetochore | High - 24 h | 1.94E-05 |
| Human | GO (BP) | GO:0060236 | regulation of mitotic spindle organization | High - 24 h | 3.15E-05 |
| Human | GO (BP) | GO:0007080 | mitotic metaphase plate congression | High - 24 h | 3.22E-05 |

| Species | Category | Term ID | Term name | Treatment | P-value |
|---|---|---|---|---|---|
| Human | GO (BP) | GO:0007094 | mitotic spindle assembly checkpoint | High - 24 h | 4.01E-05 |
| Human | GO (BP) | GO:0006206 | pyrimidine nucleobase metabolic process | High - 24 h | 4.33E-05 |
| Human | GO (BP) | GO:0006200 | ATP catabolic process | High - 24 h | 4.50E-05 |
| Human | GO (BP) | GO:0031100 | organ regeneration | High - 24 h | 5.62E-05 |
| Human | GO (BP) | GO:0051382 | kinetochore assembly | High - 24 h | 7.56E-05 |
| Human | GO (BP) | GO:0055114 | oxidation-reduction process | High - 24 h | 8.31E-05 |
| Human | GO (BP) | GO:0000910 | cytokinesis | High - 24 h | 8.57E-05 |
| Human | GO (BP) | GO:0008283 | cell proliferation | High - 24 h | 9.44E-05 |
| Human | GO (BP) | GO:0045132 | meiotic chromosome segregation | High - 24 h | 0.00010895 |
| Human | GO (BP) | GO:0007019 | microtubule depolymerization | High - 24 h | 0.00014913 |
| Human | GO (BP) | GO:0046394 | carboxylic acid biosynthetic process | High - 24 h | 0.00015846 |
| Human | GO (BP) | GO:0006297 | nucleotide-excision repair, DNA gap filling | High - 24 h | 0.00019643 |
| Human | GO (BP) | GO:0006081 | cellular aldehyde metabolic process | High - 24 h | 0.00021462 |
| Human | GO (BP) | GO:0071479 | cellular response to ionizing radiation | High - 24 h | 0.00021462 |
| Human | GO (BP) | GO:0030433 | ER-associated ubiquitin-dependent protein catabolic process | High - 24 h | 0.00021546 |
| Human | GO (BP) | GO:0090068 | positive regulation of cell cycle process | High - 24 h | 0.00030154 |
| Human | GO (BP) | GO:0006268 | DNA unwinding involved in DNA replication | High - 24 h | 0.00031637 |
| Human | GO (BP) | GO:0009157 | deoxyribonucleoside monophosphate biosynthetic process | High - 24 h | 0.00032348 |
| Human | GO (BP) | GO:0009221 | pyrimidine deoxyribonucleotide biosynthetic process | High - 24 h | 0.00032348 |
| Human | GO (BP) | GO:0071281 | cellular response to iron ion | High - 24 h | 0.00032348 |
| Human | GO (BP) | GO:0090307 | spindle assembly involved in mitosis | High - 24 h | 0.00034088 |
| Human | GO (BP) | GO:0042060 | wound healing | High - 24 h | 0.00034532 |
| Human | GO (CC) | GO:0032993 | protein-DNA complex | Low - 24 h | 6.19E-17 |
| Human | GO (CC) | GO:0000922 | spindle pole | Low - 24 h | 1.28E-12 |
| Human | GO (CC) | GO:0005829 | cytosol | Low - 24 h | 7.53E-11 |
| Human | GO (CC) | GO:0030496 | midbody | Low - 24 h | 1.31E-08 |
| Human | GO (CC) | GO:0005654 | nucleoplasm | Low - 24 h | 8.27E-08 |
| Human | GO (CC) | GO:0005876 | spindle microtubule | Low - 24 h | 2.18E-07 |
| Human | GO (CC) | GO:0072686 | mitotic spindle | Low - 24 h | 2.68E-07 |
| Human | GO (CC) | GO:0000778 | condensed nuclear chromosome kinetochore | Low - 24 h | 3.33E-07 |
| Human | GO (CC) | GO:0005871 | kinesin complex | Low - 24 h | 6.06E-06 |
| Human | GO (CC) | GO:0000796 | condensin complex | Low - 24 h | 8.61E-06 |
| Human | GO (CC) | GO:0000940 | condensed chromosome outer kinetochore | Low - 24 h | 1.12E-05 |
| Human | GO (CC) | GO:0051233 | spindle midzone | Low - 24 h | 1.74E-05 |
| Human | GO (CC) | GO:0005813 | centrosome | Low - 24 h | 4.51E-05 |
| Human | GO (CC) | GO:0042555 | MCM complex | Low - 24 h | 0.00011022 |
| Human | GO (CC) | GO:0008278 | cohesin complex | Low - 24 h | 0.00016939 |
| Human | GO (CC) | GO:0005730 | nucleolus | Low - 24 h | 0.00020934 |
| Human | GO (CC) | GO:0035371 | microtubule plus-end | Low - 24 h | 0.00064107 |

| Species | Category | Term ID | Term name | Treatment | P-value |
|---|---|---|---|---|---|
| Human | GO (CC) | GO:0045120 | pronucleus | Low - 24 h | 0.00083603 |
| Human | GO (CC) | GO:0032993 | protein-DNA complex | Middle - 24 h | 1.76E-21 |
| Human | GO (CC) | GO:0005654 | nucleoplasm | Middle - 24 h | 2.22E-12 |
| Human | GO (CC) | GO:0000922 | spindle pole | Middle - 24 h | 1.17E-11 |
| Human | GO (CC) | GO:0005829 | cytosol | Middle - 24 h | 5.35E-10 |
| Human | GO (CC) | GO:0000796 | condensin complex | Middle - 24 h | 2.70E-08 |
| Human | GO (CC) | GO:0030496 | midbody | Middle - 24 h | 1.53E-07 |
| Human | GO (CC) | GO:0000778 | condensed nuclear chromosome kinetochore | Middle - 24 h | 1.80E-07 |
| Human | GO (CC) | GO:0005876 | spindle microtubule | Middle - 24 h | 2.98E-07 |
| Human | GO (CC) | GO:0000940 | condensed chromosome outer kinetochore | Middle - 24 h | 9.16E-07 |
| Human | GO (CC) | GO:0005663 | DNA replication factor C complex | Middle - 24 h | 2.84E-06 |
| Human | GO (CC) | GO:0042555 | MCM complex | Middle - 24 h | 4.69E-06 |
| Human | GO (CC) | GO:0072686 | mitotic spindle | Middle - 24 h | 9.32E-06 |
| Human | GO (CC) | GO:0005871 | kinesin complex | Middle - 24 h | 4.48E-05 |
| Human | GO (CC) | GO:0000785 | chromatin | Middle - 24 h | 0.00021058 |
| Human | GO (CC) | GO:0005788 | endoplasmic reticulum lumen | Middle - 24 h | 0.0002234 |
| Human | GO (CC) | GO:0005813 | centrosome | Middle - 24 h | 0.00051081 |
| Human | GO (CC) | GO:0032300 | mismatch repair complex | Middle - 24 h | 0.00053004 |
| Human | GO (CC) | GO:0000407 | pre-autophagosomal structure | Middle - 24 h | 0.00053852 |
| Human | GO (CC) | GO:0051233 | spindle midzone | Middle - 24 h | 0.00071916 |
| Human | GO (CC) | GO:0035371 | microtubule plus-end | Middle - 24 h | 0.00093564 |
| Human | GO (CC) | GO:0005654 | nucleoplasm | High - 8 h | 1.40E-06 |
| Human | GO (CC) | GO:0032993 | protein-DNA complex | High - 24 h | 5.80E-21 |
| Human | GO (CC) | GO:0000922 | spindle pole | High - 24 h | 6.20E-11 |
| Human | GO (CC) | GO:0005829 | cytosol | High - 24 h | 8.18E-10 |
| Human | GO (CC) | GO:0000940 | condensed chromosome outer kinetochore | High - 24 h | 5.82E-09 |
| Human | GO (CC) | GO:0005654 | nucleoplasm | High - 24 h | 2.02E-08 |
| Human | GO (CC) | GO:0005876 | spindle microtubule | High - 24 h | 7.54E-08 |
| Human | GO (CC) | GO:0000796 | condensin complex | High - 24 h | 1.02E-07 |
| Human | GO (CC) | GO:0030496 | midbody | High - 24 h | 1.10E-07 |
| Human | GO (CC) | GO:0000778 | condensed nuclear chromosome kinetochore | High - 24 h | 6.69E-07 |
| Human | GO (CC) | GO:0070062 | extracellular vesicular exosome | High - 24 h | 6.20E-06 |
| Human | GO (CC) | GO:0005813 | centrosome | High - 24 h | 7.40E-06 |
| Human | GO (CC) | GO:0005663 | DNA replication factor C complex | High - 24 h | 8.43E-06 |
| Human | GO (CC) | GO:0042555 | MCM complex | High - 24 h | 1.68E-05 |
| Human | GO (CC) | GO:0005788 | endoplasmic reticulum lumen | High - 24 h | 0.00013482 |
| Human | GO (CC) | GO:0005871 | kinesin complex | High - 24 h | 0.0003161 |
| Human | GO (CC) | GO:0072686 | mitotic spindle | High - 24 h | 0.00035081 |
| Human | GO (CC) | GO:0032155 | cell division site part | High - 24 h | 0.00051871 |

| Species | Category | Term ID | Term name | Treatment | P-value |
|---|---|---|---|---|---|
| Human | GO (MF) | GO:0005524 | ATP binding | Low - 24 h | 2.76E-09 |
| Human | GO (MF) | GO:0008017 | microtubule binding | Low - 24 h | 1.50E-06 |
| Human | GO (MF) | GO:0003777 | microtubule motor activity | Low - 24 h | 8.25E-06 |
| Human | GO (MF) | GO:0048037 | cofactor binding | Low - 24 h | 4.04E-05 |
| Human | GO (MF) | GO:0016725 | oxidoreductase activity, acting on CH or CH2 groups | Low - 24 h | 0.00013223 |
| Human | GO (MF) | GO:0008094 | DNA-dependent ATPase activity | Low - 24 h | 0.00015474 |
| Human | GO (MF) | GO:0032405 | MutLalpha complex binding | Low - 24 h | 0.00023851 |
| Human | GO (MF) | GO:0004861 | cyclin-dependent protein serine/threonine kinase inhibitor activity | Middle - 8 h | 6.21E-06 |
| Human | GO (MF) | GO:0008017 | microtubule binding | Middle - 24 h | 4.36E-06 |
| Human | GO (MF) | GO:0005524 | ATP binding | Middle - 24 h | 4.87E-06 |
| Human | GO (MF) | GO:0008094 | DNA-dependent ATPase activity | Middle - 24 h | 1.88E-05 |
| Human | GO (MF) | GO:0032405 | MutLalpha complex binding | Middle - 24 h | 4.62E-05 |
| Human | GO (MF) | GO:0030983 | mismatched DNA binding | Middle - 24 h | 5.60E-05 |
| Human | GO (MF) | GO:0016725 | oxidoreductase activity, acting on CH or CH2 groups | Middle - 24 h | 0.00010692 |
| Human | GO (MF) | GO:0035174 | histone serine kinase activity | Middle - 24 h | 0.00013239 |
| Human | GO (MF) | GO:0004861 | cyclin-dependent protein serine/threonine kinase inhibitor activity | High - 8 h | 6.18E-06 |
| Human | GO (MF) | GO:0035257 | nuclear hormone receptor binding | High - 8 h | 8.27E-05 |
| Human | GO (MF) | GO:0008017 | microtubule binding | High - 24 h | 8.41E-06 |
| Human | GO (MF) | GO:0005524 | ATP binding | High - 24 h | 1.71E-05 |

| Species | Category | Term ID | Term name | Treatment | P-value |
|---|---|---|---|---|---|
| Rat | KEGG | rno00051 | Fructose and mannose metabolism - Rattus norvegicus (rat) | Middle - 24 h | 7.38E-05 |
| Rat | KEGG | rno00830 | Retinol metabolism - Rattus norvegicus (rat) | Middle - 24 h | 5.98E-05 |
| Rat | KEGG | rno04610 | Complement and coagulation cascades - Rattus norvegicus (rat) | Middle - 24 h | 2.46E-06 |
| Rat | KEGG | rno00260 | Glycine, serine and threonine metabolism - Rattus norvegicus (rat) | High - 24 h | 1.34E-08 |
| Rat | KEGG | rno04610 | Complement and coagulation cascades - Rattus norvegicus (rat) | High - 24 h | 2.30E-12 |
| Rat | TOX | TOX:04 | DNA Damage & Repair | High - 8 h | 0.00083009 |
| Rat | GO (BP) | GO:0055114 | oxidation-reduction process | Middle - 24 h | 5.40E-08 |
| Rat | GO (BP) | GO:0006805 | xenobiotic metabolic process | Middle - 24 h | 6.05E-06 |
| Rat | GO (BP) | GO:0017144 | drug metabolic process | Middle - 24 h | 1.01E-05 |
| Rat | GO (BP) | GO:0010043 | response to zinc ion | Middle - 24 h | 1.39E-05 |
| Rat | GO (BP) | GO:0006790 | sulfur compound metabolic process | Middle - 24 h | 7.12E-05 |
| Rat | GO (BP) | GO:0001889 | liver development | Middle - 24 h | 8.44E-05 |
| Rat | GO (BP) | GO:0010468 | regulation of gene expression | High - 8 h | 8.90E-06 |
| Rat | GO (BP) | GO:0072395 | signal transduction involved in cell cycle checkpoint | High - 8 h | 2.23E-05 |
| Rat | GO (BP) | GO:0030330 | DNA damage response, signal transduction by p53 class mediator | High - 8 h | 2.67E-05 |
| Rat | GO (BP) | GO:2000112 | regulation of cellular macromolecule biosynthetic process | High - 8 h | 2.82E-05 |
| Rat | GO (BP) | GO:0019219 | regulation of nucleobase-containing compound metabolic process | High - 8 h | 3.00E-05 |
| Rat | GO (BP) | GO:0016070 | RNA metabolic process | High - 8 h | 4.43E-05 |
| Rat | GO (BP) | GO:0010467 | gene expression | High - 8 h | 5.87E-05 |
| Rat | GO (BP) | GO:0055114 | oxidation-reduction process | High - 24 h | 5.09E-13 |
| Rat | GO (BP) | GO:0009071 | serine family amino acid catabolic process | High - 24 h | 5.31E-07 |
| Rat | GO (BP) | GO:0032787 | monocarboxylic acid metabolic process | High - 24 h | 1.82E-06 |
| Rat | GO (BP) | GO:0006958 | complement activation, classical pathway | High - 24 h | 3.06E-06 |
| Rat | GO (BP) | GO:0006544 | glycine metabolic process | High - 24 h | 3.35E-06 |
| Rat | GO (BP) | GO:1901653 | cellular response to peptide | High - 24 h | 1.43E-05 |
| Rat | GO (BP) | GO:0001889 | liver development | High - 24 h | 2.10E-05 |
| Rat | GO (BP) | GO:0006805 | xenobiotic metabolic process | High - 24 h | 2.43E-05 |
| Rat | GO (BP) | GO:0006006 | glucose metabolic process | High - 24 h | 2.62E-05 |
| Rat | GO (BP) | GO:0006695 | cholesterol biosynthetic process | High - 24 h | 3.76E-05 |
| Rat | GO (BP) | GO:0006733 | oxidoreduction coenzyme metabolic process | High - 24 h | 4.55E-05 |
| Rat | GO (BP) | GO:0042632 | cholesterol homeostasis | High - 24 h | 4.74E-05 |
| Rat | GO (BP) | GO:0044724 | single-organism carbohydrate catabolic process | High - 24 h | 5.51E-05 |
| Rat | GO (BP) | GO:0051289 | protein homotetramerization | High - 24 h | 6.92E-05 |
| Rat | GO (BP) | GO:0006790 | sulfur compound metabolic process | High - 24 h | 8.35E-05 |
| Rat | GO (BP) | GO:0009070 | serine family amino acid biosynthetic process | High - 24 h | 8.37E-05 |
| Rat | GO (BP) | GO:0009074 | aromatic amino acid family catabolic process | High - 24 h | 8.37E-05 |
| Rat | GO (BP) | GO:0072524 | pyridine-containing compound metabolic process | High - 24 h | 9.02E-05 |
| Rat | GO (BP) | GO:0043434 | response to peptide hormone | High - 24 h | 9.23E-05 |
| Rat | GO (BP) | GO:0010878 | cholesterol storage | High - 24 h | 9.44E-05 |

| Species | Category | Term ID | Term name | Treatment | P-value |
|---|---|---|---|---|---|
| Rat | GO (CC) | GO:0005615 | extracellular space | Middle - 24 h | 8.86E-05 |
| Rat | GO (CC) | GO:0005634 | nucleus | High - 8 h | 3.42E-06 |
| Rat | GO (CC) | GO:0070062 | extracellular vesicular exosome | High - 24 h | 7.36E-07 |
| Rat | GO (CC) | GO:0005615 | extracellular space | High - 24 h | 0.00010594 |
| Rat | GO (MF) | GO:0005506 | iron ion binding | Middle - 24 h | 4.35E-07 |
| Rat | GO (MF) | GO:0005543 | phospholipid binding | Middle - 24 h | 5.16E-05 |
| Rat | GO (MF) | GO:0016712 | oxidoreductase activity, acting on paired donors, with incorporation or reduction of m | Middle - 24 h | 5.45E-05 |
| Rat | GO (MF) | GO:0030170 | pyridoxal phosphate binding | High - 24 h | 2.92E-05 |
| Rat | GO (MF) | GO:0043546 | molybdopterin cofactor binding | High - 24 h | 2.95E-05 |
