# Supplementary material for: Model-based contextualization of in vitro toxicity data quantitatively predicts in vivo drug response in patients
Source: Arch Toxicol. 2016 May 9;91(2):865–83. doi: 10.1007/s00204-016-1723-x (PMC5306109; doi:10.1007/s00204-016-1723-x)
Supplement: Supplementary file 2 — Data S2 Correlation results for significantly regulated pathways and cellular processes in rats. Correlation analysis was applied between predicted drug response profiles and in vivo measurements in rats for significantly regulated pathways and cellular processes. Correlation analyses were performed by calculating Pearson’s correlation coefficient r and the corresponding p-value p (PDF 283 kb) [file 204_2016_1723_MOESM2_ESM.pdf]

Term id	Term name	r	p
rno00830	Retinol metabolism - Rattus norvegicus (rat)	0.80944	0.0014231
GO:0005506	iron ion binding	0.76305	0.0038891
GO:0006805	xenobiotic metabolic process	0.75578	0.004464
GO:0030170	pyridoxal phosphate binding	0.70727	0.010094
GO:0070062	extracellular vesicular exosome	0.68179	0.014604
GO:0006790	sulfur compound metabolic process	0.6661	0.018033
GO:0043546	molybdopterin cofactor binding	0.64673	0.023039
GO:0001889	liver development	0.63887	0.025333
GO:0005634	nucleus	0.62866	0.028551
GO:0010467	gene expression	0.62233	0.030693
GO:0006695	cholesterol biosynthetic process	0.62115	0.031104
GO:0032787	monocarboxylic acid metabolic process	0.62	0.031508
TOX:04	DNA Damage & Repair	0.61959	0.031652
GO:0055114	oxidation-reduction process	0.61273	0.034153
GO:0006006	glucose metabolic process	0.60572	0.036853
GO:0044724	single-organism carbohydrate catabolic process	0.59561	0.041004
GO:0016070	RNA metabolic process	0.59471	0.04139
GO:0051289	protein homotetramerization	0.57735	0.049333
rno04610	Complement and coagulation cascades - Rattus norvegicus (rat)	0.56126	0.057597
GO:0043434	response to peptide hormone	0.55167	0.062958
GO:2000112	regulation of cellular macromolecule biosynthetic process	0.53904	0.070532
GO:0019219	regulation of nucleobase-containing compound metabolic process	0.53508	0.073032
GO:1901653	cellular response to peptide	0.52989	0.076395
GO:0010468	regulation of gene expression	0.52147	0.082079
GO:0016712	oxidoreductase activity, acting on paired donors, with incorporation or reduction of molecular oxygen, reduced flavin or flavoprotein as one donor, and incorporation of one atom of oxygen	0.51349	0.087723
GO:0042632	cholesterol homeostasis	0.42903	0.16401
rno00260	Glycine, serine and threonine metabolism - Rattus norvegicus (rat)	0.42228	0.17148
GO:0005543	phospholipid binding	0.42213	0.17164
GO:0017144	drug metabolic process	0.3894	0.21086
GO:0009074	aromatic amino acid family catabolic process	0.38713	0.21377
GO:0010878	cholesterol storage	0.37101	0.23511

Term id	Term name	r	p
GO:0006733	oxidoreduction coenzyme metabolic process	0.35745	0.25399
GO:0072524	pyridine-containing compound metabolic process	0.33543	0.28649
GO:0006958	complement activation, classical pathway	0.32982	0.29511
GO:0030330	DNA damage response, signal transduction by p53 class mediator	0.32934	0.29586
GO:0005615	extracellular space	0.32401	0.30422
GO:0009070	serine family amino acid biosynthetic process	0.32016	0.31032
GO:0010043	response to zinc ion	0.29252	0.35619
GO:0072395	signal transduction involved in cell cycle checkpoint	-0.2679	0.39987
GO:0009071	serine family amino acid catabolic process	0.16792	0.6019
rno00051	Fructose and mannose metabolism - Rattus norvegicus (rat)	-0.10685	0.741
GO:0006544	glycine metabolic process	0.098647	0.76036
