# Supplementary material for: Model-based contextualization of in vitro toxicity data quantitatively predicts in vivo drug response in patients
Source: Arch Toxicol. 2016 May 9;91(2):865–83. doi: 10.1007/s00204-016-1723-x (PMC5306109; doi:10.1007/s00204-016-1723-x)
Supplement: Supplementary file 3 — Data S3 Deleted GO terms. Go terms for the three sub-ontologies (BP, CC, and MF) that have been deleted after applying the presented filtering method of GO terms (Supplementary Materials) (PDF 2417 kb) [file 204_2016_1723_MOESM3_ESM.pdf]

**Species**	**Sub ontology**	**Term ID**	**Term name**
Human	BP	GO:0000070	mitotic sister chromatid segregation
Human	BP	GO:0000075	cell cycle checkpoint
Human	BP	GO:0000079	regulation of cyclin-dependent protein serine/threonine kinase activity
Human	BP	GO:0000082	G1/S transition of mitotic cell cycle
Human	BP	GO:0000226	microtubule cytoskeleton organization
Human	BP	GO:0000278	mitotic cell cycle
Human	BP	GO:0000280	nuclear division
Human	BP	GO:0000723	telomere maintenance
Human	BP	GO:0000819	sister chromatid segregation
Human	BP	GO:0001702	gastrulation with mouth forming second
Human	BP	GO:0001889	liver development
Human	BP	GO:0001932	regulation of protein phosphorylation
Human	BP	GO:0001933	negative regulation of protein phosphorylation
Human	BP	GO:0003281	ventricular septum development
Human	BP	GO:0006082	organic acid metabolic process
Human	BP	GO:0006259	DNA metabolic process
Human	BP	GO:0006260	DNA replication
Human	BP	GO:0006261	DNA-dependent DNA replication
Human	BP	GO:0006281	DNA repair
Human	BP	GO:0006310	DNA recombination
Human	BP	GO:0006312	mitotic recombination
Human	BP	GO:0006323	DNA packaging
Human	BP	GO:0006333	chromatin assembly or disassembly
Human	BP	GO:0006334	nucleosome assembly
Human	BP	GO:0006336	DNA replication-independent nucleosome assembly
Human	BP	GO:0006338	chromatin remodeling
Human	BP	GO:0006351	transcription, DNA-templated
Human	BP	GO:0006355	regulation of transcription, DNA-templated
Human	BP	GO:0006357	regulation of transcription from RNA polymerase II promoter
Human	BP	GO:0006461	protein complex assembly
Human	BP	GO:0006464	cellular protein modification process
Human	BP	GO:0006468	protein phosphorylation
Human	BP	GO:0006577	amino-acid betaine metabolic process
Human	BP	GO:0006793	phosphorus metabolic process
Human	BP	GO:0006796	phosphate-containing compound metabolic process
Human	BP	GO:0006807	nitrogen compound metabolic process
Human	BP	GO:0006915	apoptotic process
Human	BP	GO:0006950	response to stress
Human	BP	GO:0006974	cellular response to DNA damage stimulus

Species	Sub ontology	Term ID	Term name
Human	BP	GO:0006984	ER-nucleus signaling pathway
Human	BP	GO:0006986	response to unfolded protein
Human	BP	GO:0006996	organelle organization
Human	BP	GO:0007010	cytoskeleton organization
Human	BP	GO:0007017	microtubule-based process
Human	BP	GO:0007049	cell cycle
Human	BP	GO:0007051	spindle organization
Human	BP	GO:0007052	mitotic spindle organization
Human	BP	GO:0007059	chromosome segregation
Human	BP	GO:0007067	mitotic nuclear division
Human	BP	GO:0007088	regulation of mitosis
Human	BP	GO:0007093	mitotic cell cycle checkpoint
Human	BP	GO:0007126	meiotic nuclear division
Human	BP	GO:0007127	meiosis I
Human	BP	GO:0007346	regulation of mitotic cell cycle
Human	BP	GO:0008152	metabolic process
Human	BP	GO:0008219	cell death
Human	BP	GO:0009056	catabolic process
Human	BP	GO:0009058	biosynthetic process
Human	BP	GO:0009059	macromolecule biosynthetic process
Human	BP	GO:0009063	cellular amino acid catabolic process
Human	BP	GO:0009112	nucleobase metabolic process
Human	BP	GO:0009123	nucleoside monophosphate metabolic process
Human	BP	GO:0009125	nucleoside monophosphate catabolic process
Human	BP	GO:0009128	purine nucleoside monophosphate catabolic process
Human	BP	GO:0009158	ribonucleoside monophosphate catabolic process
Human	BP	GO:0009169	purine ribonucleoside monophosphate catabolic process
Human	BP	GO:0009262	deoxyribonucleotide metabolic process
Human	BP	GO:0009263	deoxyribonucleotide biosynthetic process
Human	BP	GO:0009605	response to external stimulus
Human	BP	GO:0009719	response to endogenous stimulus
Human	BP	GO:0009889	regulation of biosynthetic process
Human	BP	GO:0009891	positive regulation of biosynthetic process
Human	BP	GO:0009892	negative regulation of metabolic process
Human	BP	GO:0009893	positive regulation of metabolic process
Human	BP	GO:0009987	cellular process
Human	BP	GO:0009991	response to extracellular stimulus
Human	BP	GO:0010033	response to organic substance
Human	BP	GO:0010035	response to inorganic substance

Species	Sub ontology	Term ID	Term name
Human	BP	GO:0010212	response to ionizing radiation
Human	BP	GO:0010468	regulation of gene expression
Human	BP	GO:0010556	regulation of macromolecule biosynthetic process
Human	BP	GO:0010557	positive regulation of macromolecule biosynthetic process
Human	BP	GO:0010564	regulation of cell cycle process
Human	BP	GO:0010604	positive regulation of macromolecule metabolic process
Human	BP	GO:0010605	negative regulation of macromolecule metabolic process
Human	BP	GO:0010628	positive regulation of gene expression
Human	BP	GO:0010639	negative regulation of organelle organization
Human	BP	GO:0010833	telomere maintenance via telomere lengthening
Human	BP	GO:0010941	regulation of cell death
Human	BP	GO:0010942	positive regulation of cell death
Human	BP	GO:0010948	negative regulation of cell cycle process
Human	BP	GO:0012501	programmed cell death
Human	BP	GO:0016043	cellular component organization
Human	BP	GO:0016053	organic acid biosynthetic process
Human	BP	GO:0016054	organic acid catabolic process
Human	BP	GO:0016265	death
Human	BP	GO:0016310	phosphorylation
Human	BP	GO:0018130	heterocycle biosynthetic process
Human	BP	GO:0019222	regulation of metabolic process
Human	BP	GO:0019438	aromatic compound biosynthetic process
Human	BP	GO:0019538	protein metabolic process
Human	BP	GO:0019752	carboxylic acid metabolic process
Human	BP	GO:0022402	cell cycle process
Human	BP	GO:0022616	DNA strand elongation
Human	BP	GO:0030071	regulation of mitotic metaphase/anaphase transition
Human	BP	GO:0030261	chromosome condensation
Human	BP	GO:0030968	endoplasmic reticulum unfolded protein response
Human	BP	GO:0031055	chromatin remodeling at centromere
Human	BP	GO:0031109	microtubule polymerization or depolymerization
Human	BP	GO:0031145	anaphase-promoting complex-dependent proteasomal ubiquitin-dependent protein catabolic process
Human	BP	GO:0031323	regulation of cellular metabolic process
Human	BP	GO:0031324	negative regulation of cellular metabolic process
Human	BP	GO:0031325	positive regulation of cellular metabolic process
Human	BP	GO:0031326	regulation of cellular biosynthetic process
Human	BP	GO:0031328	positive regulation of cellular biosynthetic process
Human	BP	GO:0031399	regulation of protein modification process
Human	BP	GO:0031497	chromatin assembly

Species	Sub ontology	Term ID	Term name
Human	BP	GO:0031577	spindle checkpoint
Human	BP	GO:0031667	response to nutrient levels
Human	BP	GO:0032069	regulation of nuclease activity
Human	BP	GO:0032075	positive regulation of nuclease activity
Human	BP	GO:0032200	telomere organization
Human	BP	GO:0032268	regulation of cellular protein metabolic process
Human	BP	GO:0032269	negative regulation of cellular protein metabolic process
Human	BP	GO:0032774	RNA biosynthetic process
Human	BP	GO:0032787	monocarboxylic acid metabolic process
Human	BP	GO:0032886	regulation of microtubule-based process
Human	BP	GO:0033043	regulation of organelle organization
Human	BP	GO:0033260	nuclear cell cycle DNA replication
Human	BP	GO:0033554	cellular response to stress
Human	BP	GO:0034453	microtubule anchoring
Human	BP	GO:0034508	centromere complex assembly
Human	BP	GO:0034620	cellular response to unfolded protein
Human	BP	GO:0034641	cellular nitrogen compound metabolic process
Human	BP	GO:0034645	cellular macromolecule biosynthetic process
Human	BP	GO:0034654	nucleobase-containing compound biosynthetic process
Human	BP	GO:0034724	DNA replication-independent nucleosome organization
Human	BP	GO:0034728	nucleosome organization
Human	BP	GO:0034976	response to endoplasmic reticulum stress
Human	BP	GO:0035556	intracellular signal transduction
Human	BP	GO:0035825	reciprocal DNA recombination
Human	BP	GO:0035966	response to topologically incorrect protein
Human	BP	GO:0035967	cellular response to topologically incorrect protein
Human	BP	GO:0036211	protein modification process
Human	BP	GO:0042127	regulation of cell proliferation
Human	BP	GO:0042221	response to chemical
Human	BP	GO:0042325	regulation of phosphorylation
Human	BP	GO:0042326	negative regulation of phosphorylation
Human	BP	GO:0042542	response to hydrogen peroxide
Human	BP	GO:0042592	homeostatic process
Human	BP	GO:0042981	regulation of apoptotic process
Human	BP	GO:0043044	ATP-dependent chromatin remodeling
Human	BP	GO:0043067	regulation of programmed cell death
Human	BP	GO:0043068	positive regulation of programmed cell death
Human	BP	GO:0043086	negative regulation of catalytic activity
Human	BP	GO:0043170	macromolecule metabolic process

Species	Sub ontology	Term ID	Term name
Human	BP	GO:0043412	macromolecule modification
Human	BP	GO:0043436	oxoacid metabolic process
Human	BP	GO:0043486	histone exchange
Human	BP	GO:0043549	regulation of kinase activity
Human	BP	GO:0043933	macromolecular complex subunit organization
Human	BP	GO:0044237	cellular metabolic process
Human	BP	GO:0044238	primary metabolic process
Human	BP	GO:0044248	cellular catabolic process
Human	BP	GO:0044249	cellular biosynthetic process
Human	BP	GO:0044260	cellular macromolecule metabolic process
Human	BP	GO:0044267	cellular protein metabolic process
Human	BP	GO:0044271	cellular nitrogen compound biosynthetic process
Human	BP	GO:0044281	small molecule metabolic process
Human	BP	GO:0044282	small molecule catabolic process
Human	BP	GO:0044283	small molecule biosynthetic process
Human	BP	GO:0044699	single-organism process
Human	BP	GO:0044710	single-organism metabolic process
Human	BP	GO:0044711	single-organism biosynthetic process
Human	BP	GO:0044712	single-organism catabolic process
Human	BP	GO:0044763	single-organism cellular process
Human	BP	GO:0044770	cell cycle phase transition
Human	BP	GO:0044772	mitotic cell cycle phase transition
Human	BP	GO:0044784	metaphase/anaphase transition of cell cycle
Human	BP	GO:0044786	cell cycle DNA replication
Human	BP	GO:0044839	cell cycle G2/M phase transition
Human	BP	GO:0044843	cell cycle G1/S phase transition
Human	BP	GO:0045185	maintenance of protein location
Human	BP	GO:0045786	negative regulation of cell cycle
Human	BP	GO:0045787	positive regulation of cell cycle
Human	BP	GO:0045839	negative regulation of mitosis
Human	BP	GO:0045841	negative regulation of mitotic metaphase/anaphase transition
Human	BP	GO:0045859	regulation of protein kinase activity
Human	BP	GO:0045893	positive regulation of transcription, DNA-templated
Human	BP	GO:0045935	positive regulation of nucleobase-containing compound metabolic process
Human	BP	GO:0046395	carboxylic acid catabolic process
Human	BP	GO:0046483	heterocycle metabolic process
Human	BP	GO:0048285	organelle fission
Human	BP	GO:0048518	positive regulation of biological process
Human	BP	GO:0048519	negative regulation of biological process

Species	Sub ontology	Term ID	Term name
Human	BP	GO:0048522	positive regulation of cellular process
Human	BP	GO:0048523	negative regulation of cellular process
Human	BP	GO:0048660	regulation of smooth muscle cell proliferation
Human	BP	GO:0050000	chromosome localization
Human	BP	GO:0050789	regulation of biological process
Human	BP	GO:0050790	regulation of catalytic activity
Human	BP	GO:0050794	regulation of cellular process
Human	BP	GO:0050896	response to stimulus
Human	BP	GO:0051129	negative regulation of cellular component organization
Human	BP	GO:0051171	regulation of nitrogen compound metabolic process
Human	BP	GO:0051173	positive regulation of nitrogen compound metabolic process
Human	BP	GO:0051225	spindle assembly
Human	BP	GO:0051235	maintenance of location
Human	BP	GO:0051246	regulation of protein metabolic process
Human	BP	GO:0051248	negative regulation of protein metabolic process
Human	BP	GO:0051252	regulation of RNA metabolic process
Human	BP	GO:0051254	positive regulation of RNA metabolic process
Human	BP	GO:0051276	chromosome organization
Human	BP	GO:0051293	establishment of spindle localization
Human	BP	GO:0051299	centrosome separation
Human	BP	GO:0051301	cell division
Human	BP	GO:0051302	regulation of cell division
Human	BP	GO:0051303	establishment of chromosome localization
Human	BP	GO:0051310	metaphase plate congression
Human	BP	GO:0051321	meiotic cell cycle
Human	BP	GO:0051338	regulation of transferase activity
Human	BP	GO:0051383	kinetochore organization
Human	BP	GO:0051493	regulation of cytoskeleton organization
Human	BP	GO:0051640	organelle localization
Human	BP	GO:0051716	cellular response to stimulus
Human	BP	GO:0051726	regulation of cell cycle
Human	BP	GO:0051783	regulation of nuclear division
Human	BP	GO:0051784	negative regulation of nuclear division
Human	BP	GO:0051983	regulation of chromosome segregation
Human	BP	GO:0060255	regulation of macromolecule metabolic process
Human	BP	GO:0061008	hepaticobiliary system development
Human	BP	GO:0061641	CENP-A containing chromatin organization
Human	BP	GO:0065003	macromolecular complex assembly
Human	BP	GO:0065004	protein-DNA complex assembly

Species	Sub ontology	Term ID	Term name
Human	BP	GO:0065007	biological regulation
Human	BP	GO:0065008	regulation of biological quality
Human	BP	GO:0065009	regulation of molecular function
Human	BP	GO:0070271	protein complex biogenesis
Human	BP	GO:0070507	regulation of microtubule cytoskeleton organization
Human	BP	GO:0070887	cellular response to chemical stimulus
Human	BP	GO:0071103	DNA conformation change
Human	BP	GO:0071173	spindle assembly checkpoint
Human	BP	GO:0071174	mitotic spindle checkpoint
Human	BP	GO:0071310	cellular response to organic substance
Human	BP	GO:0071704	organic substance metabolic process
Human	BP	GO:0071822	protein complex subunit organization
Human	BP	GO:0071824	protein-DNA complex subunit organization
Human	BP	GO:0071840	cellular component organization or biogenesis
Human	BP	GO:0071900	regulation of protein serine/threonine kinase activity
Human	BP	GO:0071901	negative regulation of protein serine/threonine kinase activity
Human	BP	GO:0072527	pyrimidine-containing compound metabolic process
Human	BP	GO:0080090	regulation of primary metabolic process
Human	BP	GO:0090224	regulation of spindle organization
Human	BP	GO:1901342	regulation of vasculature development
Human	BP	GO:1901360	organic cyclic compound metabolic process
Human	BP	GO:1901362	organic cyclic compound biosynthetic process
Human	BP	GO:1901564	organonitrogen compound metabolic process
Human	BP	GO:1901565	organonitrogen compound catabolic process
Human	BP	GO:1901575	organic substance catabolic process
Human	BP	GO:1901576	organic substance biosynthetic process
Human	BP	GO:1901605	alpha-amino acid metabolic process
Human	BP	GO:1901987	regulation of cell cycle phase transition
Human	BP	GO:1901988	negative regulation of cell cycle phase transition
Human	BP	GO:1901990	regulation of mitotic cell cycle phase transition
Human	BP	GO:1901991	negative regulation of mitotic cell cycle phase transition
Human	BP	GO:1902099	regulation of metaphase/anaphase transition of cell cycle
Human	BP	GO:1902100	negative regulation of metaphase/anaphase transition of cell cycle
Human	BP	GO:1902589	single-organism organelle organization
Human	BP	GO:1902680	positive regulation of RNA biosynthetic process
Human	BP	GO:1902850	microtubule cytoskeleton organization involved in mitosis
Human	BP	GO:1903046	meiotic cell cycle process
Human	BP	GO:1903047	mitotic cell cycle process
Human	BP	GO:2000112	regulation of cellular macromolecule biosynthetic process

Species	Sub ontology	Term ID	Term name
Human	BP	GO:2001141	regulation of RNA biosynthetic process
Human	MF	GO:0000166	nucleotide binding
Human	MF	GO:0001882	nucleoside binding
Human	MF	GO:0001883	purine nucleoside binding
Human	MF	GO:0003690	double-stranded DNA binding
Human	MF	GO:0003824	catalytic activity
Human	MF	GO:0004860	protein kinase inhibitor activity
Human	MF	GO:0005515	protein binding
Human	MF	GO:0015631	tubulin binding
Human	MF	GO:0016491	oxidoreductase activity
Human	MF	GO:0016538	cyclin-dependent protein serine/threonine kinase regulator activity
Human	MF	GO:0016887	ATPase activity
Human	MF	GO:0017076	purine nucleotide binding
Human	MF	GO:0019207	kinase regulator activity
Human	MF	GO:0019210	kinase inhibitor activity
Human	MF	GO:0019887	protein kinase regulator activity
Human	MF	GO:0030291	protein serine/threonine kinase inhibitor activity
Human	MF	GO:0030554	adenyl nucleotide binding
Human	MF	GO:0032404	mismatch repair complex binding
Human	MF	GO:0032549	ribonucleoside binding
Human	MF	GO:0032550	purine ribonucleoside binding
Human	MF	GO:0032553	ribonucleotide binding
Human	MF	GO:0032555	purine ribonucleotide binding
Human	MF	GO:0032559	adenyl ribonucleotide binding
Human	MF	GO:0035173	histone kinase activity
Human	MF	GO:0035639	purine ribonucleoside triphosphate binding
Human	MF	GO:0036094	small molecule binding
Human	MF	GO:0043168	anion binding
Human	MF	GO:0043566	structure-specific DNA binding
Human	MF	GO:0097367	carbohydrate derivative binding
Human	MF	GO:1901265	nucleoside phosphate binding
Human	CC	GO:0000228	nuclear chromosome
Human	CC	GO:0000775	chromosome, centromeric region
Human	CC	GO:0000776	kinetochore
Human	CC	GO:0000777	condensed chromosome kinetochore
Human	CC	GO:0000779	condensed chromosome, centromeric region
Human	CC	GO:0000780	condensed nuclear chromosome, centromeric region
Human	CC	GO:0000793	condensed chromosome
Human	CC	GO:0000794	condensed nuclear chromosome

Species	Sub ontology	Term ID	Term name
Human	CC	GO:0005622	intracellular
Human	CC	GO:0005623	cell
Human	CC	GO:0005634	nucleus
Human	CC	GO:0005657	replication fork
Human	CC	GO:0005694	chromosome
Human	CC	GO:0005737	cytoplasm
Human	CC	GO:0005783	endoplasmic reticulum
Human	CC	GO:0005815	microtubule organizing center
Human	CC	GO:0005819	spindle
Human	CC	GO:0005856	cytoskeleton
Human	CC	GO:0005874	microtubule
Human	CC	GO:0005875	microtubule associated complex
Human	CC	GO:0015630	microtubule cytoskeleton
Human	CC	GO:0031974	membrane-enclosed lumen
Human	CC	GO:0031981	nuclear lumen
Human	CC	GO:0031988	membrane-bounded vesicle
Human	CC	GO:0032153	cell division site
Human	CC	GO:0043226	organelle
Human	CC	GO:0043227	membrane-bounded organelle
Human	CC	GO:0043228	non-membrane-bounded organelle
Human	CC	GO:0043229	intracellular organelle
Human	CC	GO:0043230	extracellular organelle
Human	CC	GO:0043231	intracellular membrane-bounded organelle
Human	CC	GO:0043232	intracellular non-membrane-bounded organelle
Human	CC	GO:0043233	organelle lumen
Human	CC	GO:0044421	extracellular region part
Human	CC	GO:0044422	organelle part
Human	CC	GO:0044424	intracellular part
Human	CC	GO:0044427	chromosomal part
Human	CC	GO:0044428	nuclear part
Human	CC	GO:0044430	cytoskeletal part
Human	CC	GO:0044432	endoplasmic reticulum part
Human	CC	GO:0044444	cytoplasmic part
Human	CC	GO:0044446	intracellular organelle part
Human	CC	GO:0044454	nuclear chromosome part
Human	CC	GO:0044464	cell part
Human	CC	GO:0044815	DNA packaging complex
Human	CC	GO:0065010	extracellular membrane-bounded organelle
Human	CC	GO:0070013	intracellular organelle lumen

**Species**	**Sub ontology**	**Term ID**	**Term name**
Human	CC	GO:1990391	DNA repair complex

Species	Sub ontology	Term ID	Term name
Rat	BP	GO:0005996	monosaccharide metabolic process
Rat	BP	GO:0006066	alcohol metabolic process
Rat	BP	GO:0006082	organic acid metabolic process
Rat	BP	GO:0006520	cellular amino acid metabolic process
Rat	BP	GO:0006629	lipid metabolic process
Rat	BP	GO:0006694	steroid biosynthetic process
Rat	BP	GO:0006725	cellular aromatic compound metabolic process
Rat	BP	GO:0006732	coenzyme metabolic process
Rat	BP	GO:0006950	response to stress
Rat	BP	GO:0006956	complement activation
Rat	BP	GO:0008152	metabolic process
Rat	BP	GO:0008202	steroid metabolic process
Rat	BP	GO:0008203	cholesterol metabolic process
Rat	BP	GO:0008652	cellular amino acid biosynthetic process
Rat	BP	GO:0009056	catabolic process
Rat	BP	GO:0009063	cellular amino acid catabolic process
Rat	BP	GO:0009069	serine family amino acid metabolic process
Rat	BP	GO:0009410	response to xenobiotic stimulus
Rat	BP	GO:0009725	response to hormone
Rat	BP	GO:0009889	regulation of biosynthetic process
Rat	BP	GO:0010556	regulation of macromolecule biosynthetic process
Rat	BP	GO:0016052	carbohydrate catabolic process
Rat	BP	GO:0016053	organic acid biosynthetic process
Rat	BP	GO:0016054	organic acid catabolic process
Rat	BP	GO:0016125	sterol metabolic process
Rat	BP	GO:0016126	sterol biosynthetic process
Rat	BP	GO:0019222	regulation of metabolic process
Rat	BP	GO:0019318	hexose metabolic process
Rat	BP	GO:0019752	carboxylic acid metabolic process
Rat	BP	GO:0031323	regulation of cellular metabolic process
Rat	BP	GO:0033554	cellular response to stress
Rat	BP	GO:0043436	oxoacid metabolic process
Rat	BP	GO:0044237	cellular metabolic process
Rat	BP	GO:0044238	primary metabolic process
Rat	BP	GO:0044248	cellular catabolic process
Rat	BP	GO:0044281	small molecule metabolic process
Rat	BP	GO:0044282	small molecule catabolic process
Rat	BP	GO:0044283	small molecule biosynthetic process
Rat	BP	GO:0044710	single-organism metabolic process

Species	Sub ontology	Term ID	Term name
Rat	BP	GO:0044711	single-organism biosynthetic process
Rat	BP	GO:0044712	single-organism catabolic process
Rat	BP	GO:0046394	carboxylic acid biosynthetic process
Rat	BP	GO:0046395	carboxylic acid catabolic process
Rat	BP	GO:0046483	heterocycle metabolic process
Rat	BP	GO:0050789	regulation of biological process
Rat	BP	GO:0050794	regulation of cellular process
Rat	BP	GO:0051171	regulation of nitrogen compound metabolic process
Rat	BP	GO:0051186	cofactor metabolic process
Rat	BP	GO:0055088	lipid homeostasis
Rat	BP	GO:0055092	sterol homeostasis
Rat	BP	GO:0061008	hepaticobiliary system development
Rat	BP	GO:0071466	cellular response to xenobiotic stimulus
Rat	BP	GO:0071704	organic substance metabolic process
Rat	BP	GO:0072331	signal transduction by p53 class mediator
Rat	BP	GO:0072376	protein activation cascade
Rat	BP	GO:0080090	regulation of primary metabolic process
Rat	BP	GO:1901360	organic cyclic compound metabolic process
Rat	BP	GO:1901564	organonitrogen compound metabolic process
Rat	BP	GO:1901575	organic substance catabolic process
Rat	BP	GO:1901605	alpha-amino acid metabolic process
Rat	BP	GO:1901606	alpha-amino acid catabolic process
Rat	BP	GO:1901615	organic hydroxy compound metabolic process
Rat	BP	GO:1901652	response to peptide
Rat	BP	GO:1901700	response to oxygen-containing compound
Rat	CC	GO:0005576	extracellular region
Rat	CC	GO:0005622	intracellular
Rat	CC	GO:0031982	vesicle
Rat	CC	GO:0031988	membrane-bounded vesicle
Rat	CC	GO:0043226	organelle
Rat	CC	GO:0043227	membrane-bounded organelle
Rat	CC	GO:0043230	extracellular organelle
Rat	CC	GO:0044421	extracellular region part
Rat	CC	GO:0044424	intracellular part
Rat	CC	GO:0065010	extracellular membrane-bounded organelle
Rat	MF	GO:0003824	catalytic activity
Rat	MF	GO:0004497	monooxygenase activity
Rat	MF	GO:0016491	oxidoreductase activity
Rat	MF	GO:0016705	oxidoreductase activity, acting on paired donors, with incorporation or reduction of molecular oxygen

Species	Sub ontology	Term ID	Term name
Rat	MF	GO:0043168	anion binding
Rat	MF	GO:0048037	cofactor binding
Rat	MF	GO:0050662	coenzyme binding
