# Supplementary material for: Model-based contextualization of in vitro toxicity data quantitatively predicts in vivo drug response in patients
Source: Arch Toxicol. 2016 May 9;91(2):865–83. doi: 10.1007/s00204-016-1723-x (PMC5306109; doi:10.1007/s00204-016-1723-x)
Supplement: Supplementary file 4 — Supplementary Materials (DOCX 37 kb) [file 204_2016_1723_MOESM4_ESM.docx]

# Supplementary Materials

## Toxicogenomics database

Time-series gene expression profiles from Open TG-Gates (Igarashi et al. 2015) (ArrayExpress accession numbers: E-MTAB-797, E-MTAB-798, E-MTAB-799), a large-scale toxicogenomics database, were used to obtain quantitative drug response data measured in human and rat hepatocytes as well as in rat livers. Human and rat hepatocytes were exposed to three different concentrations (low, middle, and high). In the original in vitro assay, the highest concentration was selected such that cell viability was decreased by 10-20 % (Igarashi et al. 2015). The low and middle concentrations were then determined by diluting the highest concentration by five and twenty-five, respectively (Igarashi et al. 2015). For the in vivo study, a minimum toxic dose identified in a 4-week toxicity study was set as highest dose, while the low and middle dose were one third and one tenth of the high dose, respectively (Igarashi et al. 2015). Gene expression levels were measured after three exposure durations (2 h, 8 h, and 24 h) in the in vitro study and after four exposure durations (3 h, 6 h, 9 h, 24 h) in the in vivo study leading to nine and twelve different treatments, respectively. In addition, time-dependent gene expression data of control samples were collected. Fold change values were calculated to indicate gene expression changes compared to the time-matched controls. Cell viability of human and rat hepatocytes was assessed by measuring the total DNA content. Cytotoxicity in each treatment was calculated by the difference of total DNA content between treated hepatocytes and their particular time-matched controls.

## Filtering of gene ontology terms

In gene ontology, genes and gene products are annotated with biological terms from the three different sub-ontologies biological process (BP), cellular component (CC), and molecular function (MF) (Ashburner et al. 2000). These GO terms and their relations are represented as a directed acyclic graph (DAG). In this graph, lower levels characterizing higher specialization. To analyze only significantly affected GO terms with a high degree of specialization, all enriched GO terms having a significant enriched descendant in the DAG were filtered out. An example of this filtering procedure is illustrated in Fig. S10.

## Software

All PBPK models were built by using the software PK-Sim® (Eissing et al. 2011; Willmann et al. 2003; Willmann et al. 2005; Willmann et al. 2004) (version 5.4, Bayer Technology Services, GmbH, Leverkusen, Germany) and MoBi® (version 3.4, Bayer Technology Services), which are freely available for academic use. Transcriptome analysis was performed in the statistical language R (version 3.1.0, 2014, R Core Team, <http://www.R-project.org>). PICD was implemented in MATLAB (version 8.3.0; The MathWorks, Inc., Natick, MA) by use of the MoBi® Toolbox for MATLAB (version 2.3; Bayer Technology Services GmbH). The interaction network of genes that are involved in processes of DNA damage and repair was built by the use of QIAGEN’s Ingenuity Pathway Analysis (IPA®, QIAGEN Redwood City, [www.qiagen.com/ingenuity](http://www.qiagen.com/ingenuity)).

# REFERENCES

Ashburner, M. et al., 2000. Gene ontology: tool for the unification of biology. The Gene Ontology Consortium. *Nature genetics*, 25(1), pp.25–9.

Eissing, T. et al., 2011. A computational systems biology software platform for multiscale modeling and simulation: Integrating whole-body physiology, disease biology, and molecular reaction networks. *Frontiers in Physiology*, 2(4).

Gregoriano, C. et al., 2014. Acute thiopurine overdose: Analysis of reports to a national poison centre 1995-2013. *PLoS ONE*, 9(1), p.e86390.

Igarashi, Y. et al., 2015. Open TG-GATEs: a large-scale toxicogenomics database. *Nucleic Acids Res*, 43, pp.D921–7.

Persson, H.E. et al., 1998. Poisoning severity score. Grading of acute poisoning. *Journal of toxicology. Clinical toxicology*, 36(3), pp.205–213.

Thiel, C. et al., 2015. A systematic evaluation of the use of physiologically based pharmacokinetic modeling for cross-species extrapolation. *Journal of pharmaceutical sciences*, 104(1), pp.191–206.

Willmann, S. et al., 2004. A pharmacodynamic extension for the physiology-based pharmacokinetic whole-body model PK-Sim((R)). *European Journal of Pharmaceutical Sciences*, 23, pp.S75–S75.

Willmann, S. et al., 2003. PK-Sim®: a physiologically based pharmacokinetic “whole-body” model. *BIOSILICO*, 1(4), pp.121–124.

Willmann, S., Lippert, J. & Schmitt, W., 2005. From physicochemistry to absorption and distribution: predictive mechanistic modelling and computational tools. *Expert opinion on drug metabolism & toxicology*, 1(1), pp.159–68.
