# Supplementary figures and images for: Model-based contextualization of in vitro toxicity data quantitatively predicts in vivo drug response in patients
Source: Arch Toxicol. 2016 May 9;91(2):865–83. doi: 10.1007/s00204-016-1723-x (PMC5306109; doi:10.1007/s00204-016-1723-x)

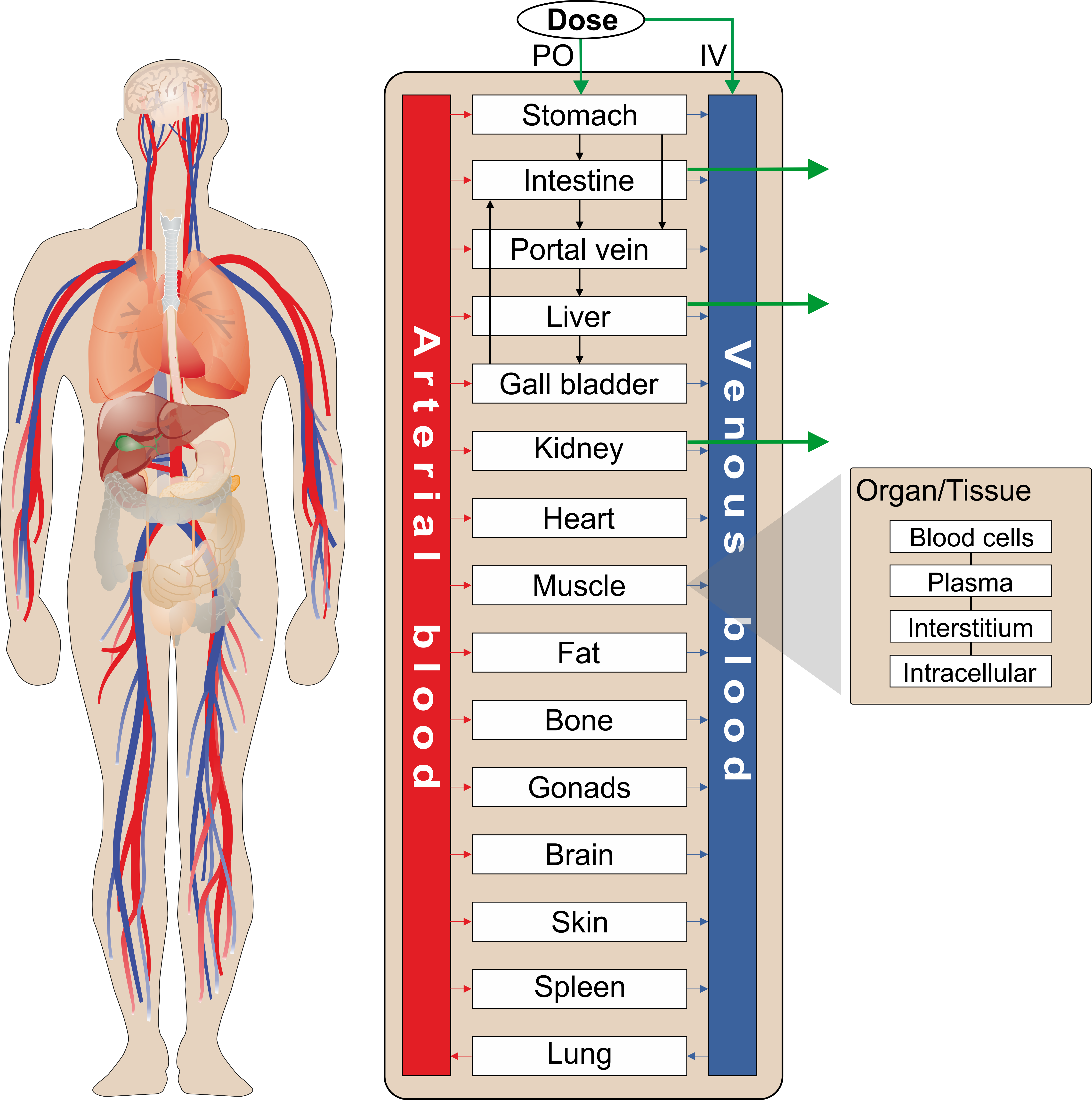

Supplement: Supplementary file 5 — Figure S1 Schematic representation of a multiscale whole-body PBPK model. Schematic representation of a multiscale whole-body PBPK model including 15 different tissues and organs that are connected by blood flow. Sub-compartmentalization is exemplarily presented for a default compartment (PNG 1415 kb) [file 204_2016_1723_MOESM5_ESM.png]

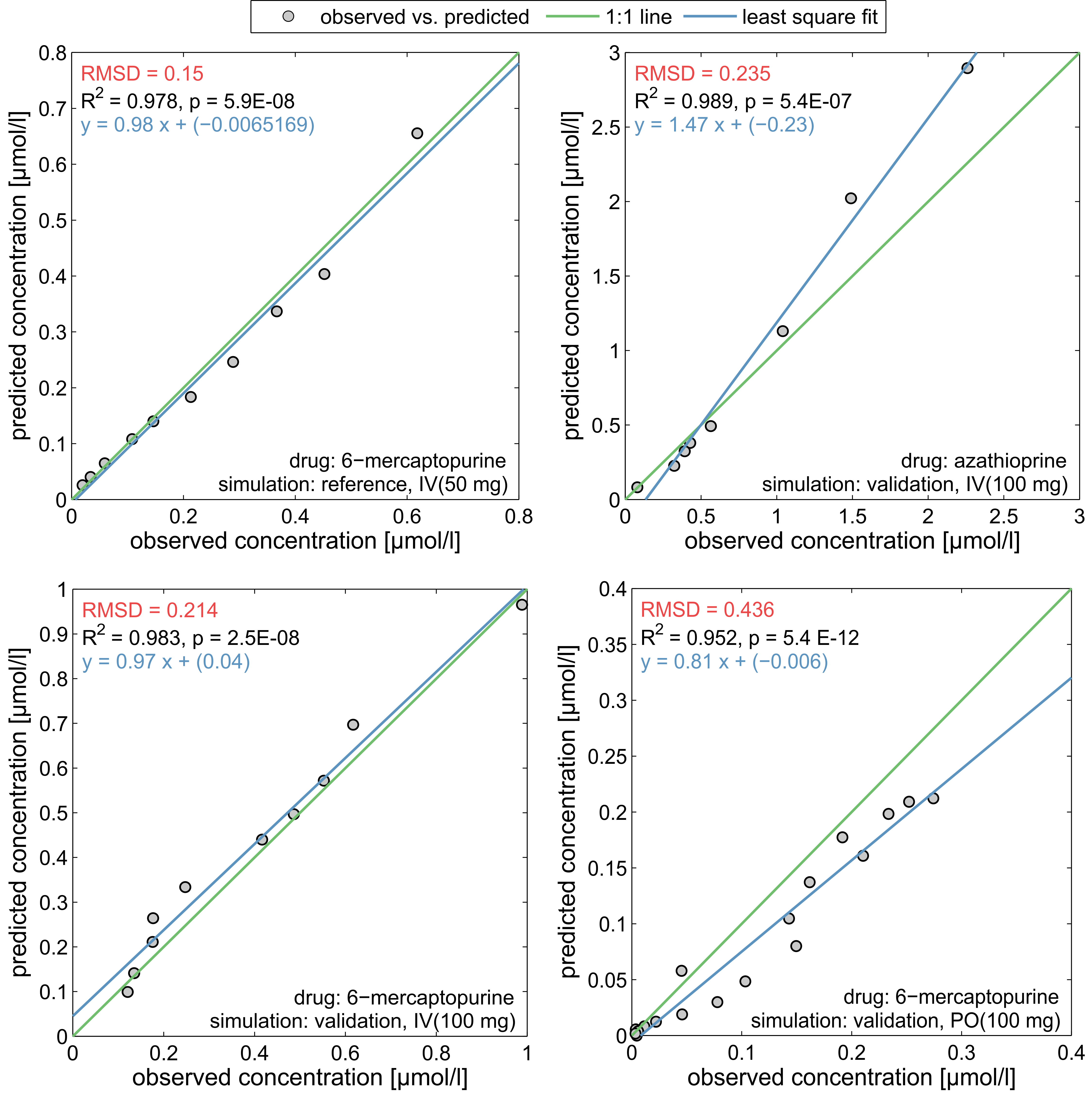

Supplement: Supplementary file 6 — Figure S2 PBPK model assessment. Simulated concentration-time profiles were compared to experimental data. Observed vs. predicted plots including RMSD value, coefficient of determination (R2), and the equation of the linear regression were generated for the reference and validated PBPK model. Simulated concentration-time profiles were compared to experimental data. Observed vs. predicted plots including RMSD value, coefficient of determination (R2), and the equation of the linear regression were generated for the reference and validated PBPK model (PNG 1248 kb) [file 204_2016_1723_MOESM6_ESM.png]

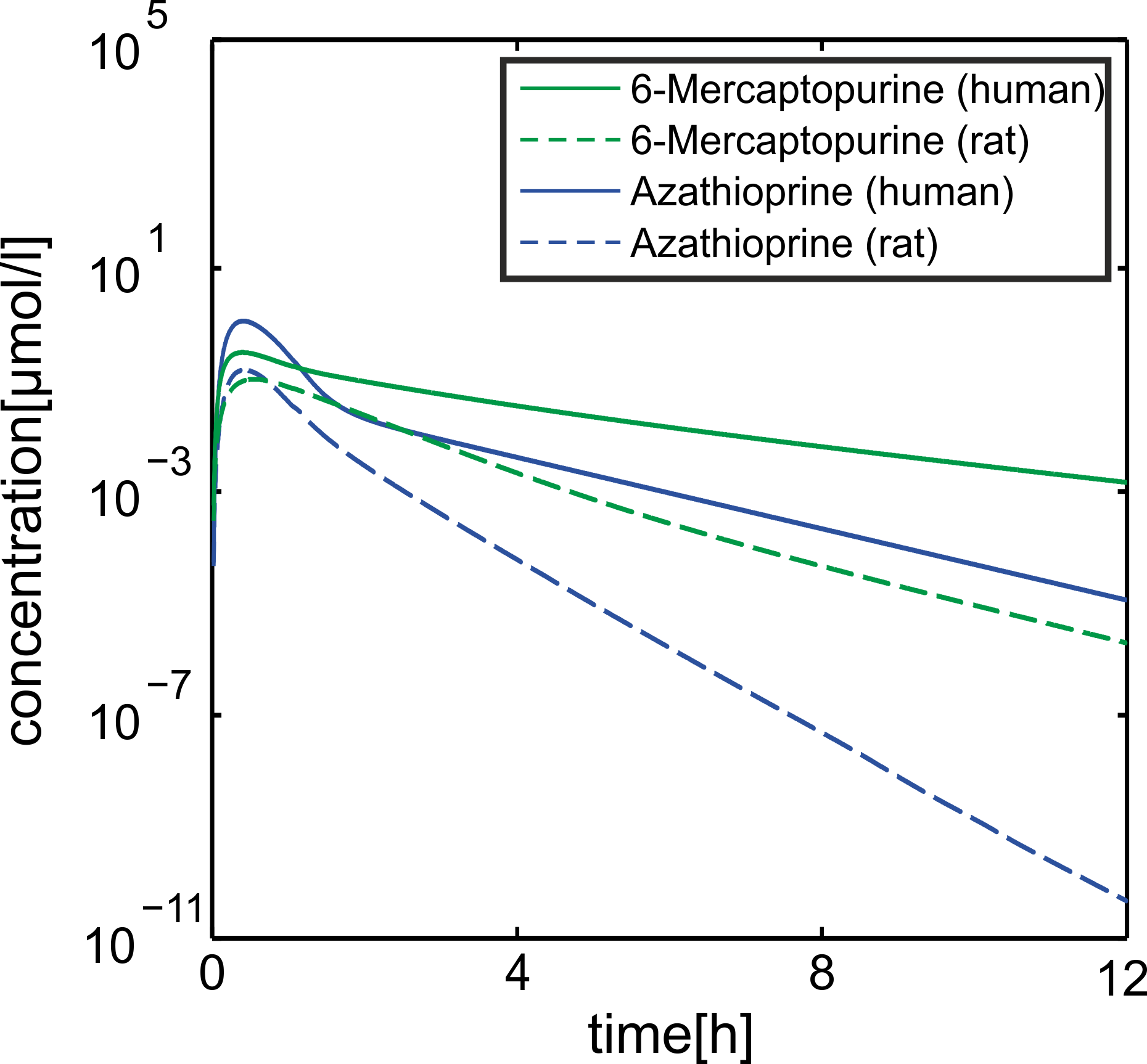

Supplement: Supplementary file 7 — Figure S3 Rat PBPK model. Blood plasma concentration-time profiles of azathioprine and 6-mercaptopurine were simulated for rats (dashed blue line, dashed green line) and for humans (solid blue line, solid green line) after oral administration of 100 mg of azathioprine. The rat PBPK model of azathioprine was developed by considering rat-specific anatomy and physiology in the human PBPK model according to (Thiel et al. 2015) (PNG 212 kb) [file 204_2016_1723_MOESM7_ESM.png]

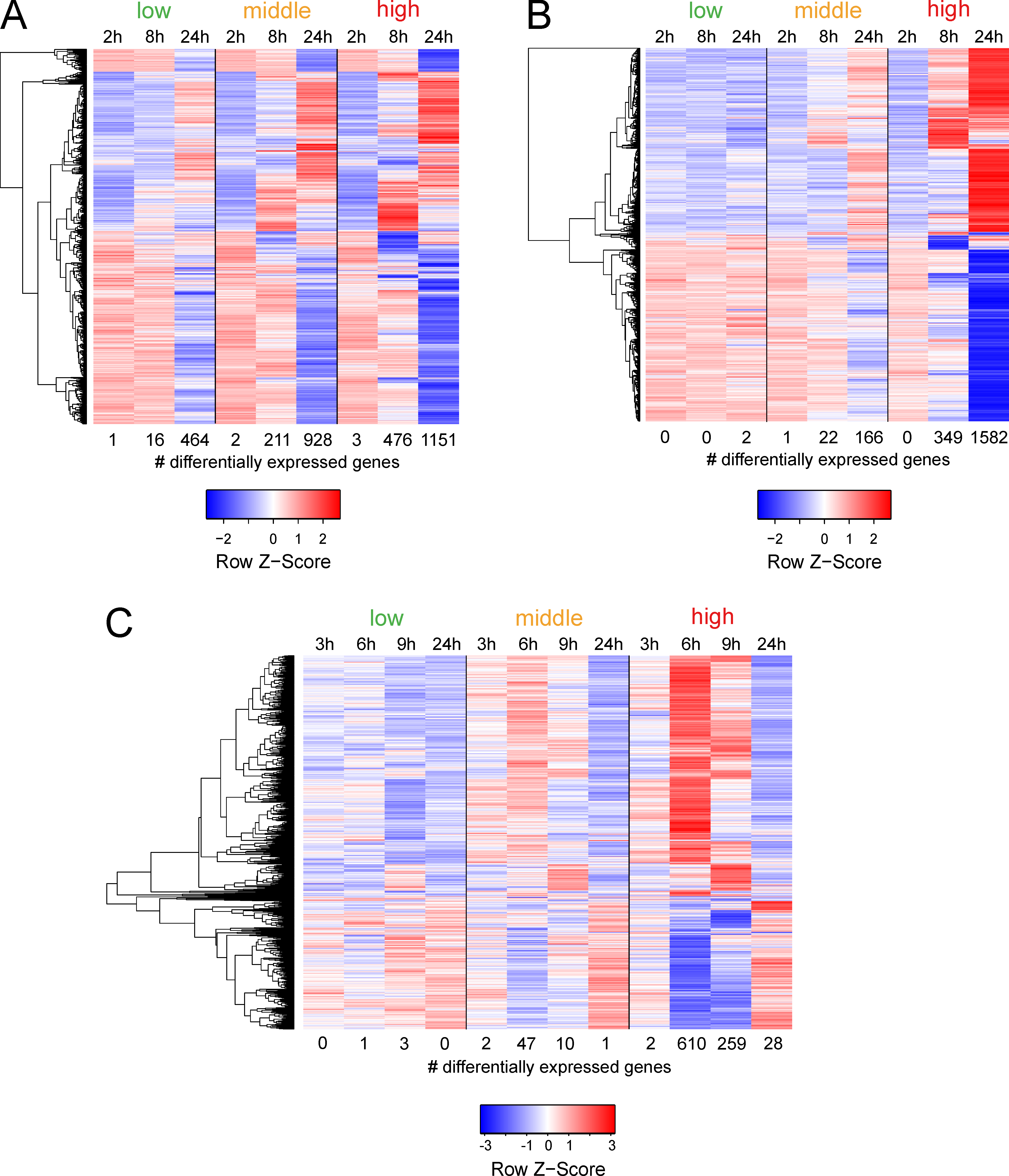

Supplement: Supplementary file 8 — Figure S4 Azathioprine-induced in vitro and in vivo gene expression data. Heatmaps of in vitro and in vivo expression data of genes that were differentially expressed in at least one treatment of the specific experiment (Igarashi et al. 2015). Three different exposure levels of azathioprine were administered (low (green), middle (orange), high (red)) and gene expression was measured after three and four different exposure durations in the in vitro and in vivo case, respectively. The number below each column indicates the number of differentially expressed genes identified in the specific treatment. Gene expression values in each row were z-score normalized. (A) In vitro gene expression data obtained in primary human hepatocytes. (B) In vitro gene expression data obtained in primary rat hepatocytes. (C) In vivo gene expression data obtained in rats (PNG 446 kb) [file 204_2016_1723_MOESM8_ESM.png]

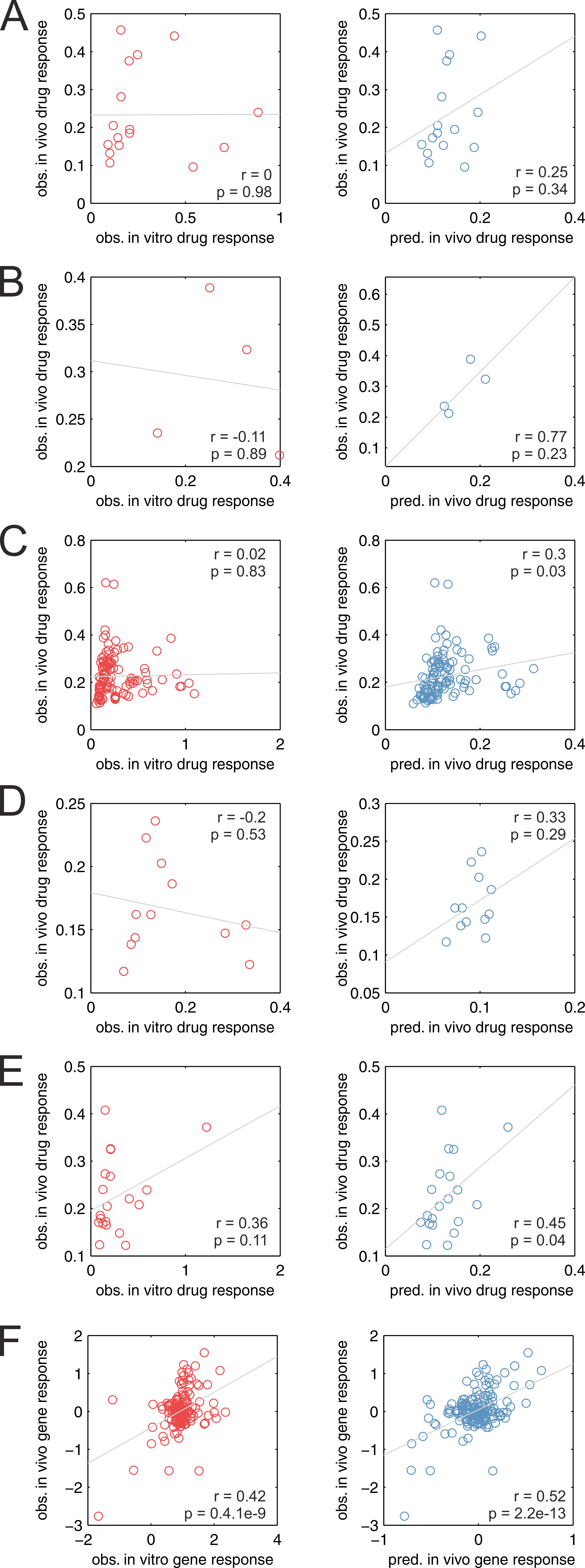

Supplement: Supplementary file 9 — Figure S5 Correlation between observed in vivo drug response and both predicted in vivo drug response and observed in vitro drug response. Predicted in vivo drug response (blue) induced by the identified toxic dose (Igarashi et al. 2015) as well as corresponding in vitro profiles (red) induced by the toxic concentration (Igarashi et al. 2015) were correlated with measurements observed in vivo (Igarashi et al. 2015). All cellular processes or biological pathways that were significantly regulated in at least one treatment (Data S1) and all genes analyzed in both case studies (Table S4, Table S6) were considered for the correlation of drug response and gene expression, respectively. Correlation analyses were performed by calculating Pearson’s correlation coefficient r and the corresponding p-value p. (A) Correlation of significantly affected KEGG pathways. (B) Correlation of significantly affected toxicity-related pathways. (C) Correlation of significantly affected biological processes. (D) Correlation of significantly affected cellular components. (E) Correlation of significantly affected molecular functions. (F) Correlation of genes considered in both case studies (PNG 1750 kb) [file 204_2016_1723_MOESM9_ESM.png]

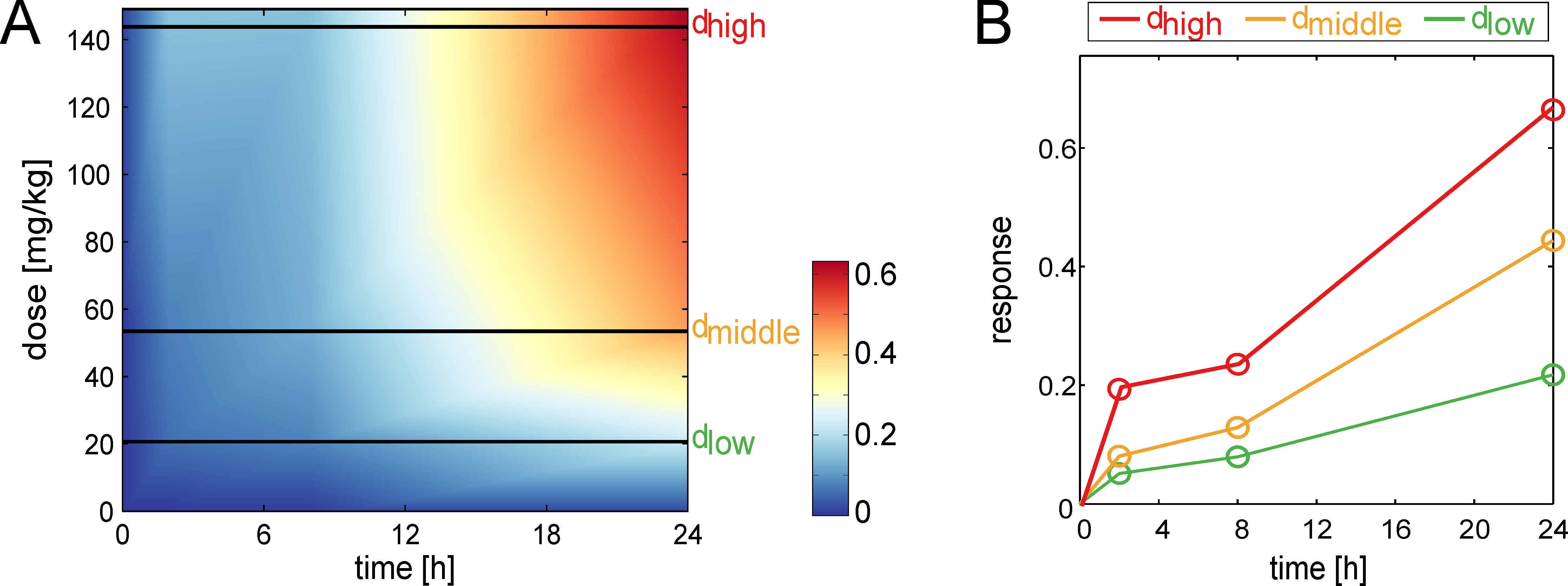

Supplement: Supplementary file 11 — Figure S7 Predicted in vivo drug response of DNA replication. (A) Drug response map exemplarily shown for DNA replication reflecting time- and dose-dependent effects following administration of azathioprine at dose levels dlow, dmiddle and dhigh (black lines). The color scale depicts predicted in vivo drug responses. (B) Predicted in vivo drug response over time induced by doses dlow, dmiddle and dhigh (PNG 147 kb) [file 204_2016_1723_MOESM11_ESM.png]

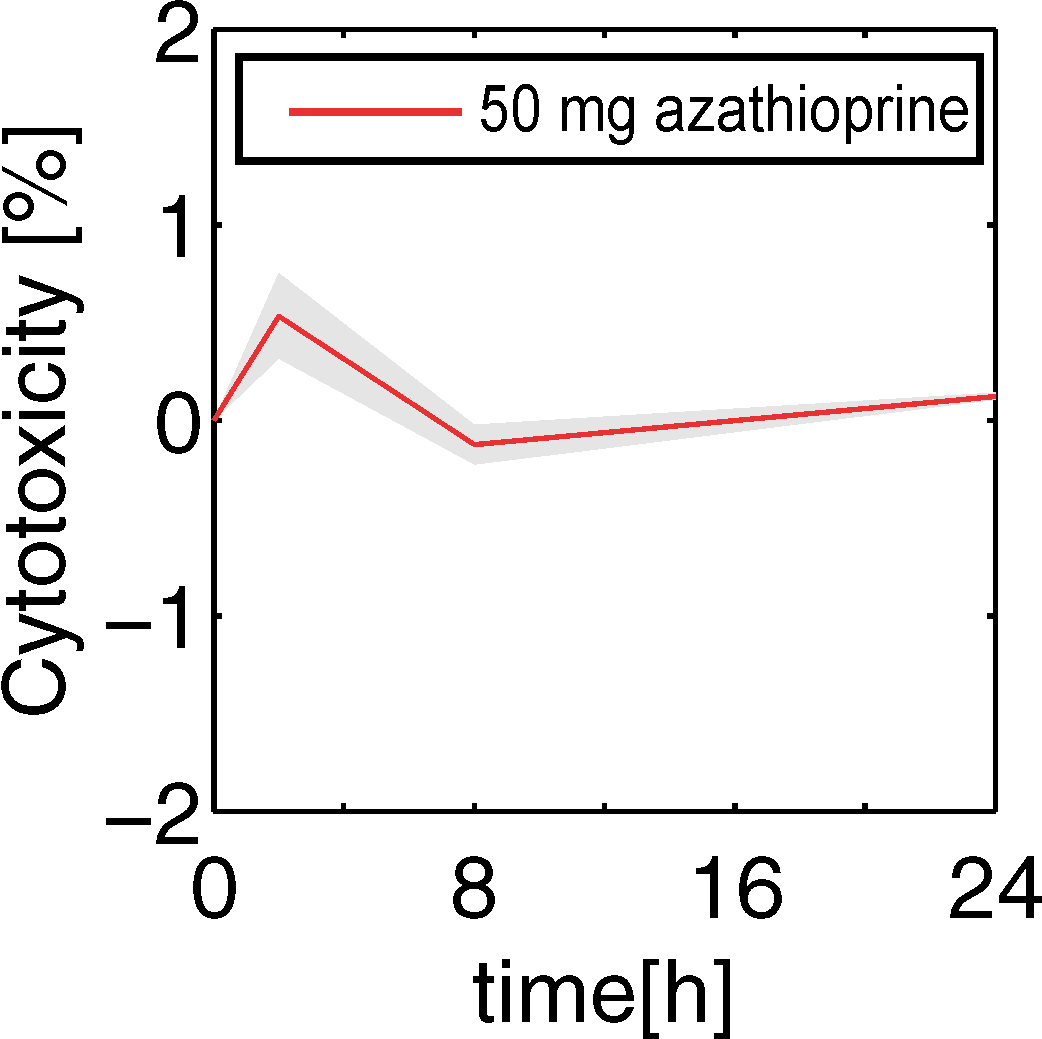

Supplement: Supplementary file 12 — Figure S8 Predicted in vivo cytotoxicity over time. In vivo cytotoxicity values over time induced by the therapeutic dose were predicted for both replicates (gray area). The mean cytotoxicity values are shown as solid line (PNG 18 kb) [file 204_2016_1723_MOESM12_ESM.png]

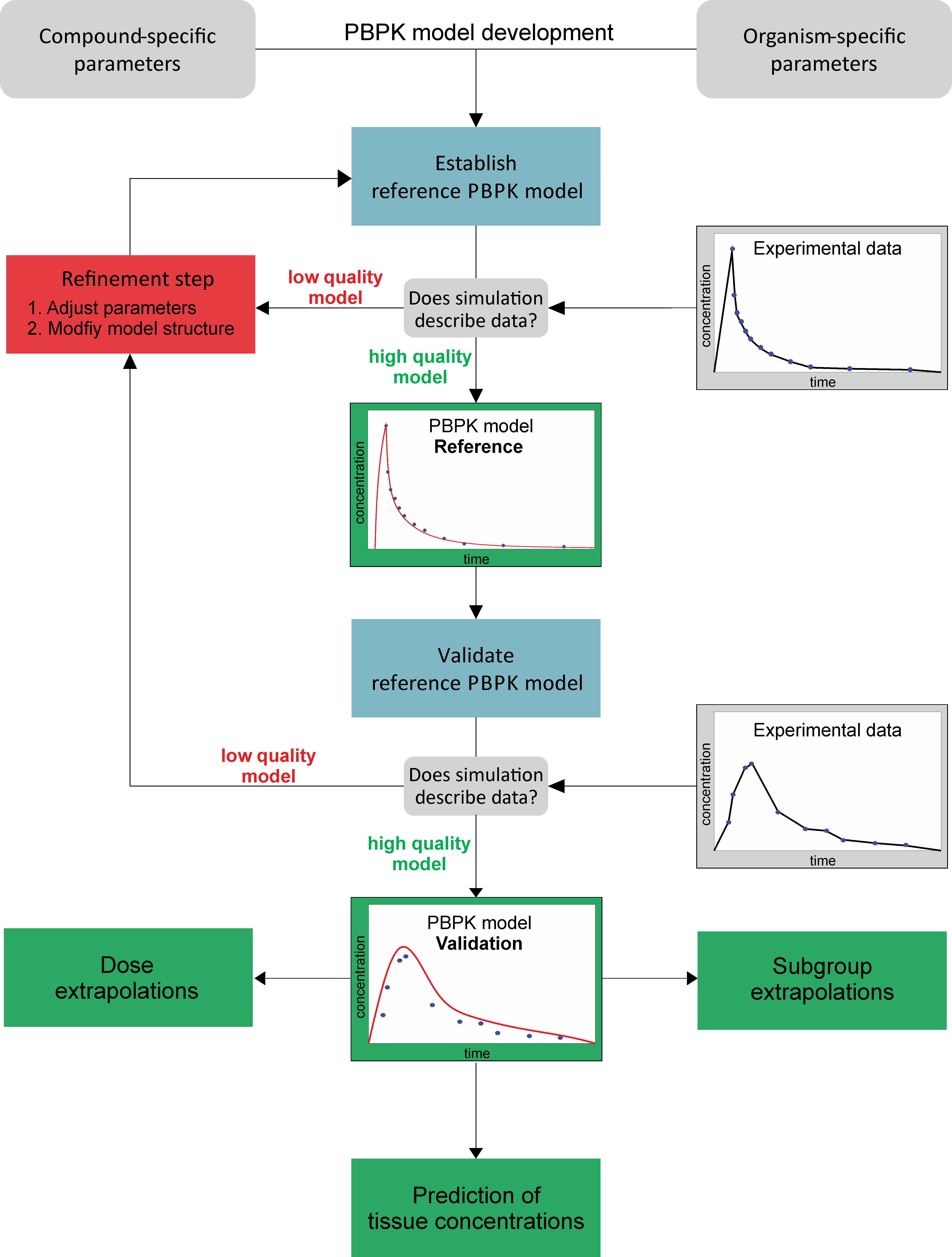

Supplement: Supplementary file 13 — Figure S9 Workflow for PBPK model development and validation. After parametrizing compound-specific properties and organism-specific parameters in the reference PBPK model, the model quality is evaluated by comparing simulated drug concentrations with experimental data from literature. If a sufficient model accuracy has been reached, a subsequent validation step enables reliable model extrapolations. Amongst others, this validation step ensures accurate predictions of concentration-time profiles in various compartments. Otherwise, the PBPK model is revised in a refinement step thereby adjusting key model parameters or adding more active transport processes or metabolizing reactions to improve the description of physiological processes governing the fate of the considered compound within the body (PNG 184 kb) [file 204_2016_1723_MOESM13_ESM.png]

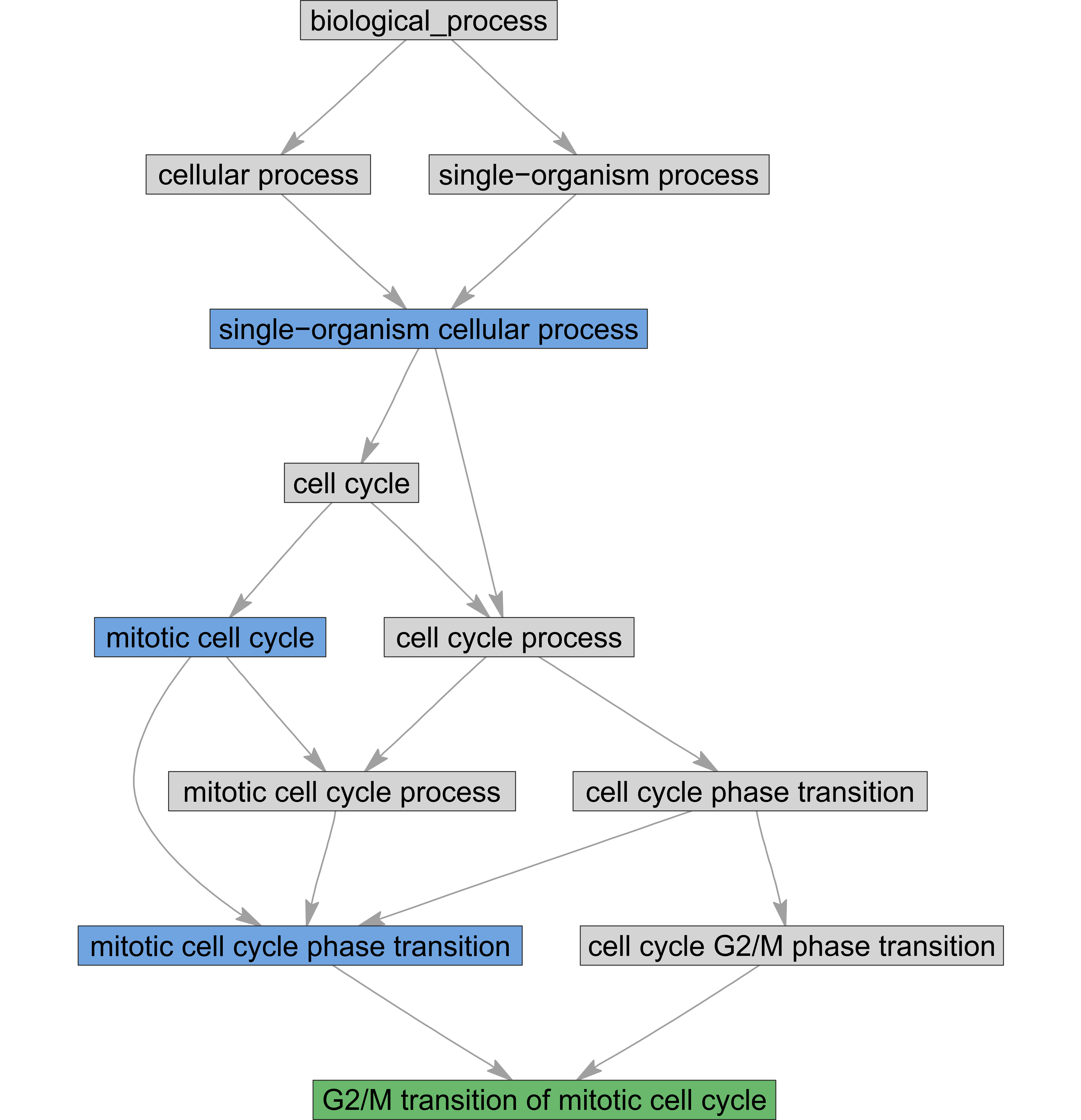

Supplement: Supplementary file 14 — Figure S10 Filtering gene ontology terms. The presented graph illustrates an exemplary subgraph of the complete GO graph. In total, four biological processes were identified as significantly enriched (blue and green nodes). After applying the filtering procedure, three terms were filtered out (blue nodes) while one remained for further analysis (green). Note that the green node represents the highest specialization (PNG 754 kb) [file 204_2016_1723_MOESM14_ESM.png]
