# Supplementary material for: Model-based contextualization of in vitro toxicity data quantitatively predicts in vivo drug response in patients
Source: Arch Toxicol. 2016 May 9;91(2):865–83. doi: 10.1007/s00204-016-1723-x (PMC5306109; doi:10.1007/s00204-016-1723-x)
Supplement: Supplementary file 15 — Table S1 Toxicity-related biological pathways. Symbols as well as human and rat Entrez IDs for 370 genes showing high response to toxic compounds were grouped in thirteen different biological pathways. The genes and the functional gene grouping terms were taken from the Human Molecular Toxicology PathwayFinder RT2 ProfilerTM PCR Array (SABiosciences, http://www.sabiosciences.com). Rat Entrez IDs were identified through the use of QIAGEN’s Ingenuity Pathway Analysis (IPA®, QIAGEN Redwood City, www.qiagen.com/ingenuity) (DOCX 41 kb) [file 204_2016_1723_MOESM15_ESM.docx]

### Table S1. Toxicity-related biological pathways.

Symbols as well as human and rat Entrez IDs for 370 genes showing high response to toxic compounds were grouped in thirteen different biological pathways. The genes and the functional gene grouping terms were taken from the Human Molecular Toxicology PathwayFinder RT² Profiler™ PCR Array (SABiosciences, <http://www.sabiosciences.com>). Rat Entrez IDs were identified through the use of QIAGEN’s Ingenuity Pathway Analysis (IPA®, QIAGEN Redwood City, [www.qiagen.com/ingenuity](http://www.qiagen.com/ingenuity)).

| **ID** | **Term name** | **Symbols** | **Entrez IDs (human)** | **Entrez IDs (rat)** |
| --- | --- | --- | --- | --- |
| TOX:01 | Apoptosis | ABL1, AKT1, APAF1, BAD, BAK1, BAX, BCL2, BCL2L1, BCL2L11, BID, BIRC3, CASP1, CASP3, CASP7, CASP8, CASP9, CD40, CD40LG, CFLAR, FADD, FAS, FASLG, GADD45A, MCL1, TNF, TNFRSF10A, TNFRSF10B, TNFRSF1A, TNFSF10, TP53, XIAP | 25, 207, 317, 572, 578, 581, 596, 598, 10018, 637, 330, 834, 836, 840, 841, 842, 958, 959, 8837, 8772, 355, 356, 1647, 4170, 7124, 8797, 8795, 7132, 8743, 7157, 331 | 311860, 24185, 78963, 64639, 116502, 24887, 24224, 24888, 64547, 64625, 78971, 25166, 25402, 64026, 64044, 58918, 171369, 84349, 117279, 266610, 246097, 25385, 25112, 60430, 24835, 364420, not found , 25625, 246775, 24842, 63879 |
| TOX:02 | Cholestasis | ABCB1, ABCB4, ABCC1, ABCC2, ABCC3, APOE, ATP8B1, CYP3A4, CYP7A1, DLAT, ESR1, HLA-DRB1, ICAM1, IL10, IL1B, IL2, IL6, JAG1, MPO, NR1H4, NUP210, OSTALPHA, OSTBETA, PDYN, RDX, SLC10A1, TGFB1, TNF | 5243, 5244, 4363, 1244, 8714, 348, 5205, 1576, 1581, 1737, 2099, 3123, 3383, 3586, 3553, 3558, 3569, 182, 4353, 9971, 23225, 200931, 123264, 5173, 5962, 6554, 7040, 7124 | 170913, 24891, 24565, 25303, 140668, 25728, 291555, 266682, 25428, 81654, 24890, not found , 25464, 25325, 24494, 116562, 24498, 29146, not found , 60351, 58958, 29190, 315655, 24777, 303879, 300790, 59086, 24835 |
| TOX:03 | Cytochrome P450s & Phase I Drug Metabolism | CYP1A1, CYP1A2, CYP2B6, CYP2C19, CYP2C9, CYP2D6, CYP2E1, CYP3A4, ESD, FMO2, FMO3, FMO4, FMO5, MAOA, MAOB | 1543, 1544, 1555, 1557, 1559, 1565, 1571, 1576, 2098, 2327, 2328, 2329, 2330, 4128, 4129 | 24296, 24297, 24300, 293989, 29277, 24303, 25086, 266682, 290401, 246245, 84493, 246247, 246248, 29253, 25750 |
| TOX:04 | DNA Damage & Repair | APEX1, ATM, ATR, BRCA1, BRCA2, CDKN1A, CHEK1, CHEK2, DDIT3, ERCC1, ERCC2, ERCC3, ERCC5, ERCC6, GADD45A, LIG4, MDM2, MGMT, MLH1, MSH2, OGG1, PARP1, PCNA, PRKDC, RAD51, TP53, XPA, XPC, XRCC1, XRCC5 | 328, 472, 545, 672, 675, 1026, 1111, 11200, 1649, 2067, 2068, 2071, 2073, 2074, 1647, 3981, 4193, 4255, 4292, 4436, 4968, 142, 5111, 5591, 5888, 7157, 7507, 7508, 7515, 7520 | 79116, 300711, 685055, 497672, 360254, 114851, 140583, 114212, 29467, 292673, 308415, 291703, 301382, 306274, 25112, 290907, 314856, 25332, 81685, 81709, 81528, 25591, 25737, 360748, 499870, 24842, 298074, 312560, 84495, 363247 |
| TOX:05 | ER Stress & Unfolded Protein Response | AMFR, ATF4, ATF6, BAX, DDIT3, DERL1, EDEM1, EDEM3, EIF2AK3, ERN2, ERO1L, ERO1LB, FBXO6, GADD45A, HERPUD1, HTRA2, HTRA4, MBTPS1, MBTPS2, NPLOC4, NUCB1, OS9, PFDN5, PPIA, SEC62, SEL1L, SELS, SERP1, SYVN1, UBE2G2, UBE2J2, UBXN4, VCP, XBP1 | 267, 468, 22926, 581, 1649, 79139, 9695, 80267, 9451, 10595, 30001, 56605, 26270, 1647, 9709, 27429, 203100, 8720, 51360, 55666, 4924, 10956, 5204, 5478, 7095, 6400, 55829, 27230, 84447, 7327, 118424, 23190, 7415, 7495 | 361367, 79255, 304962, 24887, 29467, 362912, 297504, 289085, 29702, 365363, 171562, 364755, 192351, 25112, 85430, 297376, 306564, 89842, 302705, 140639, 84595, 362891, 300257, 25518, 294912, 314352, 80881, 361712, 294331, 298689, 304766, 116643, 286900, not found |
| TOX:06 | Fatty Acid Metabolism | ACAA1, ACAA2, ACAD11, ACAD9, ACADL, ACADM, ACADS, ACADSB, ACADVL, ACAT1, ACAT2, ACOT1, ACOT12, ACOT2, ACOT6, ACOT7, ACOT8, ACOT9, ACOX1, ACOX2, ACOX3, CPT1A, CPT1B, CPT2, CRAT, CROT, ECHS1, EHHADH, GCDH, HADHA | 30, 10449, 84129, 28976, 33, 34, 35, 36, 37, 38, 39, 641371, 134526, 10965, 641372, 11332, 10005, 23597, 51, 8309, 8310, 1374, 1375, 1376, 1384, 54677, 1892, 1962, 2639, 3030 | 24157, 170465, 315973, 294973, 25287, 24158, 64304, 25618, 25363, 25014, 308100, 314304, 170570, 192272, not found , 26759, 170588, 302640, 50681, 252898, 83522, 25757, 25756, 25413, 311849, 83842, 140547, 171142, 364975, 170670 |
| TOX:07 | Heat Shock Response | CRYAA, CRYAB, DNAJA1, DNAJA2, DNAJA3, DNAJB1, DNAJB6, DNAJC3, DNAJC5, DNAJC6, HSF1, HSF2, HSP90AA1, HSP90AB1, HSP90B1, HSPA1A, HSPA1B, HSPA1L, HSPA2, HSPA4, HSPA5, HSPA8, HSPA9, HSPB1, HSPB2, HSPB6, HSPB8, HSPD1, HSPE1, HSPH1, TCP1 | 1409, 1410, 3301, 10294, 9093, 3337, 10049, 5611, 80331, 9829, 3297, 3298, 3320, 3326, 7184, 3303, 3304, 3305, 3306, 3308, 3309, 3312, 3313, 3315, 3316, 126393, 26353, 3329, 3336, 10808, 6950 | 24273, 25420, 65028, 84026, 360481, 361384, 362293, 63880, 79130, 313409, 79245, 64441, 299331, 301252, 362862, 294254\|24472, 24963, 60460, 266759, 25617, 24468, 291671, 24471, 161476, 192245, 113906, 63868, 25462, 288444, 24818 |
| TOX:08 | Immunotoxicity | ADH1C, AHR, AHSG, ALB, APOA5, APOF, C3, C9, CASP3, CD19, CD4, CD44, CD80, CD86, CD8A, CTSE, CYP1A1, CYP3A4, CYP3A4, EP300, F2, FABP1, FAS, GPT, GSTA3, HPX, HRG, HSPA5, IFNA1, IFNG, IL10, IL13, IL1A, IL1B, IL2, IL4, IL5, IL6, ITGAX, KLF1, LYZ, LYZ, METAP2, MKI67, NFKB1, NR5A2, PON1, POU3F3, PTGS2, PTPRC, SOD1, TNF, TRIM10, UBQLN2 | 126, 196, 197, 213, 116519, 319, 718, 735, 836, 930, 920, 960, 941, 942, 925, 1510, 1543, 1576, 1576, 2033, 2147, 2168, 355, 2875, 2940, 3263, 3273, 3309, 3439, 3458, 3586, 3596, 3552, 3553, 3558, 3565, 3567, 3569, 3687, 10661, 4069, 4069, 10988, 4288, 4790, 2494, 5444, 5455, 5743, 5788, 6647, 7124, 10107, 29978 | 24172, 25690, 25373, 24186, 140638, 500761, 24232, 117512, 25402, 365367, 24932, 25406, 25408, 56822, 24930, 25424, 24296, 266682, 170915, 29251, 24360, 246097, 81670, 494500, 58917, not found , 25617, not found , 25712, 25325, 116553, 24493, 24494, 116562, 287287, 24497, 24498, 499271, 304666, 25211, 64370, 291234, 81736, 60349, 84024, 192109, 29527, 24699, 24786, 24835, 294210, 317396 |
| TOX:09 | Mitochondrial Energy Metabolism | ACLY, ACO1, ACO2, COX6B1, COX8A, CS, CYC1, DLD, DLST, FH, IDH1, IDH2, IDH3A, IDH3B, IDH3G, MDH1, MDH1B, MDH2, OGDH, SDHA, SDHB, SDHC, SDHD, SUCLA2, SUCLG1, SUCLG2, UCP1, UCP2, UCP3 | 47, 48, 50, 1340, 1351, 1431, 1537, 1738, 1743, 2271, 3417, 3418, 3419, 3420, 3421, 4190, 130752, 4191, 4967, 6389, 6390, 6391, 6392, 8803, 8802, 8801, 7350, 7351, 7352 | 24159, 50655, 79250, 688869, 171335, 170587, 300047, 298942, 299201, 24368, 24479, 361596, 114096, 94173, 25179, 24551, 316444, 81829, 360975, 157074, 298596, 289217, 363061, 361071, 114597, 362404, 24860, 54315, 25708 |
| TOX:10 | Necrosis | ATP6V1G2, BMF, CCDC103, CD300LD, CLEC18A, COMMD4, CYLD, DEFB1, DPYSL4, EIF5B, FOXI1, GALNT5, GRB2, HOXA3, HSPBAP1, JPH3, KCNIP1, MAG, NUDT13, OR10J3, PARP2, PVR, RAB25, S100A7A, SPATA2, SYCP2, TMEM57, TNFAIP8L1, TNFRSF1A, TXNL4B | 534, 90427, 388389, 100131439, 348174, 54939, 1540, 1672, 10570, 9669, 2299, 11227, 2885, 3200, 79663, 57338, 30820, 4099, 25961, 441911, 10038, 5817, 57111, 338324, 9825, 10388, 55219, 126282, 7132, 54957 | 368044, 246142, 498006, 360655, not found , 363068, 312937, not found , 25417, 308306, 287185, 83627, 81504, 500125, 171460, 307916, 65023, 29409, 682978, 289240, 290027, not found , 310632, not found , 114210, 83820, 313618, 301131, 25625, 292008 |
| TOX:11 | Oxidative Stress & Antioxidant Response | AASS, CAT, CTSB, DHCR24, DUOX1, DUOX2, EPX, GPX1, GPX2, GPX3, GPX4, GPX5, GPX6, GPX7, IDH1, MPO, NQO1, NUDT1, NUDT15, PPP1R15B, PRDX1, PRDX2, PRDX6, SOD1, TPO, TXNIP, TXNRD2, UCP3 | 10157, 847, 1508, 1718, 53905, 50506, 8288, 2876, 2877, 2878, 2879, 2880, 257202, 2882, 3417, 4353, 1728, 4521, 55270, 84919, 5052, 7001, 9588, 6647, 7173, 10628, 10587, 7352 | 296925, 24248, 64529, 298298, 266807, 79107, 303414, 24404, 29326, 64317, 29328, 113919, 259233, 298376, 24479, not found , 24314, 117260, 290365, 304799, 117254, 29338, 94167, 24786, 54314, 117514, 50551, 25708 |
| TOX:12 | Phospholipidosis | ABCB1, ALDH1A1, ASAH1, ASNS, CES2, CTSB, EPHX1, FABP1, FXC1, GSTM4, HPN, INHBE, LSS, MANBA, MLX, MRPS18B, NR0B2, POR, S100A8, SC4MOL, SERPINA3, SLC2A3, SLCO1A2, SMPD1, STBD1, TAGLN, UGT1A1, UGT2A1, UGT2B4, WIPI1 | 5243, 216, 427, 440, 8824, 1508, 2052, 2168, 26515, 2948, 3249, 83729, 4047, 4126, 6945, 28973, 8431, 5447, 6279, 6307, 12, 6515, 6579, 6609, 8987, 6876, 54658, 10941, 7363, 55062 | 170913, 24188, 84431, 25612, 498940, 64529, 25315, 24360, 499689, 29135, 83711, 81681, 310864, 360631, 294230, 140910, 117274, 29441, 116547, 24795, 25551, 80900, 308909, 305234, 25123, 84384, 24861, 63867, 286989, 303630 |
| TOX:13 | Steatosis | ACACA, ADK, ALDH2, AQP4, CD36, COMT, CYP2E1, CYP7B1, DNM1, ENO1, FAS, FASN, GPD1, HAAO, HADHB, KHK, LMNA, LPL, LY6D, MAPK8, MTTP, PCCA, PNPLA3, PPARA, RETN, SCD, SREBF1, SYT1, TFF3, VCP | 31, 132, 217, 361, 948, 1312, 1571, 9420, 1759, 2023, 355, 2194, 2819, 23498, 3032, 3795, 4000, 4023, 8581, 5599, 4547, 5095, 80339, 5465, 56729, 6319, 6720, 6857, 7033, 7415 | 60581, 25368, 29539, 25293, 29184, 24267, 25086, 25429, 140694, 24333, 246097, 50671, 60666, 56823, 171155, 25659, 60374, 24539, 315075, 116554, 310900, 687008, 362972, 25747, 246250, 246074, 78968, 25716, 25563, 116643 |
