# Supplementary material for: Model-based contextualization of in vitro toxicity data quantitatively predicts in vivo drug response in patients
Source: Arch Toxicol. 2016 May 9;91(2):865–83. doi: 10.1007/s00204-016-1723-x (PMC5306109; doi:10.1007/s00204-016-1723-x)
Supplement: Supplementary file 16 — Table S2 Dose identification. In vitro concentration, exposure duration, resulting in vitro exposure, and identified in vivo dose for the specific treatments for rats and humans (DOCX 26 kb) [file 204_2016_1723_MOESM16_ESM.docx]

### Table S2. Dose identification.

In vitro concentration, exposure duration, resulting in vitro exposure, and identified in vivo dose for the specific treatments for rats and humans.

| **Species** | **Treatment** | **In vitro concentration [µmol/l]** | **Exposure duration [h]** | **In vitro exposure [µmol/l/h]** | **In vivo dose**  **[mg/kg]** |
| --- | --- | --- | --- | --- | --- |
| Human | Low – 2 h | 2.9 | 2 | 5.8 | 9.0 |
| Human | Low – 8 h | 2.9 | 8 | 23.2 | 18.8 |
| Human | Low – 24 h | 2.9 | 24 | 69.6 | 34.4 |
| Human | Middle – 2 h | 14.6 | 2 | 29.2 | 21.4 |
| Human | Middle – 8 h | 14.6 | 8 | 116.8 | 46.6 |
| Human | Middle – 24 h | 14.6 | 24 | 350.4 | 91.8 |
| Human | High – 2 h | 72.8 | 2 | 145.6 | 53.4 |
| Human | High – 8 h | 72.8 | 8 | 582.4 | 126.8 |
| Human | High – 24 h | 72.8 | 24 | 1747.2 | 248.3 |
| Rat | Low – 2 h | 0.14 | 2 | 0.28 | 2.2 |
| Rat | Low – 8 h | 0.14 | 8 | 1.12 | 5.6 |
| Rat | Low – 24 h | 0.14 | 24 | 3.36 | 10.1 |
| Rat | Middle – 2 h | 0.72 | 2 | 1.44 | 6.5 |
| Rat | Middle – 8 h | 0.72 | 8 | 5.76 | 13.3 |
| Rat | Middle – 24 h | 0.72 | 24 | 17.28 | 23.5 |
| Rat | High – 2 h | 3.6 | 2 | 7.2 | 14.9 |
| Rat | High – 8 h | 3.6 | 8 | 28.8 | 31.2 |
| Rat | High – 24 h | 3.6 | 24 | 86.4 | 58.0 |
