# Supplementary material for: Model-based contextualization of in vitro toxicity data quantitatively predicts in vivo drug response in patients
Source: Arch Toxicol. 2016 May 9;91(2):865–83. doi: 10.1007/s00204-016-1723-x (PMC5306109; doi:10.1007/s00204-016-1723-x)
Supplement: Supplementary file 17 — Table S3 Clinical cases of acute azathioprine overdose. Anthropometric parameters (age, weight, and sex), administered dose, and observed symptoms including assigned Poisoning Severity Scores (PSS) (Persson et al. 1998). The clinical data were taken from (Gregoriano et al. 2014) (DOCX 28 kb) [file 204_2016_1723_MOESM17_ESM.docx]

### Table S3. Clinical cases of acute azathioprine overdose.

Anthropometric parameters (age, weight, and sex), administered dose, and observed symptoms including assigned Poisoning Severity Scores (PSS) (Persson et al. 1998). The clinical data were taken from (Gregoriano et al. 2014).

| **Patient ID** | **Age [years]** | **Weight [kg]** | **Sex** | **Dose [mg/kg]** | **Symptoms (PSS)** |
| --- | --- | --- | --- | --- | --- |
| 19 | 28 | 71 | Male | 180.1 | nausea (1), abdominal pain (1), headache (1), increased liver enzymes (10-fold increase in transaminases from baseline) (2), dyspnoea (2), fall in leucocyte count (7.2 to 3.9 G/l) (1) |
| 17 | 53 | 60 | Female | 107.5 | Asymptomatic (0) |
| 28 | 49 | 76 | Male | 32.9 | Increased GGT (< 2 x) (1) |
| 21 | 39 | 73 | Male | 27.0 | Headache (1), vomiting (1) |
| 16 | 44 | 75 | Female | 26.7 | Sinus tachycardia (1) |
| 04 | 39 | 50 | Female | 16.0 | Asymptomatic (0) |
| 13 | 23 | 63 | Female | 11.9 | Vomiting (1) |
| 33 | 28 | 65 | Male | 6.9 | Asymptomatic (0) |
