# Supplementary material for: Model-based contextualization of in vitro toxicity data quantitatively predicts in vivo drug response in patients
Source: Arch Toxicol. 2016 May 9;91(2):865–83. doi: 10.1007/s00204-016-1723-x (PMC5306109; doi:10.1007/s00204-016-1723-x)
Supplement: Supplementary file 18 — Table S4 Genes involved in the DNA damage & repair pathway. Symbols, Entrez gene name, type, as well as human and rat Entrez identifier for all genes involved in the DNA damage & repair pathway. Functional classifications were taken from QIAGEN’s Ingenuity Pathway Analysis (IPA®, QIAGEN Redwood City, www.qiagen.com/ingenuity) (DOCX 27 kb) [file 204_2016_1723_MOESM18_ESM.docx]

### Table S4. Genes involved in the DNA damage & repair pathway.

Symbols, Entrez gene name, type, as well as human and rat Entrez identifier for all genes involved in the DNA damage & repair pathway. Functional classifications were taken from QIAGEN’s Ingenuity Pathway Analysis (IPA®, QIAGEN Redwood City, www.qiagen.com/ingenuity).

| **Symbol** | **Entrez gene name** | **Type** | **Entrez ID (human)** | **Entrez ID (rat)** |
| --- | --- | --- | --- | --- |
| APEX1 | APEX nuclease (multifunctional DNA repair enzyme) 1 | enzyme | 328 | 79116 |
| ATM | ATM serine/threonine kinase | kinase | 472 | 300711 |
| ATR | ATR serine/threonine kinase | kinase | 545 | 685055 |
| BRCA1 | breast cancer 1, early onset | transcription regulator | 672 | 497672 |
| BRCA2 | breast cancer 2, early onset | transcription regulator | 675 | 360254 |
| CDKN1A | cyclin-dependent kinase inhibitor 1A (p21, Cip1) | kinase | 1026 | 114851 |
| CHEK1 | checkpoint kinase 1 | kinase | 1111 | 140583 |
| CHEK2 | checkpoint kinase 2 | kinase | 11200 | 114212 |
| DDIT3 | DNA-damage-inducible transcript 3 | transcription regulator | 1649 | 29467 |
| ERCC1 | excision repair cross-complementation group 1 | enzyme | 2067 | 292673 |
| ERCC2 | excision repair cross-complementation group 2 | enzyme | 2068 | 308415 |
| ERCC3 | excision repair cross-complementation group 3 | enzyme | 2071 | 291703 |
| ERCC5 | excision repair cross-complementation group 5 | enzyme | 2073 | 301382 |
| ERCC6 | excision repair cross-complementation group 6 | transcription regulator | 2074 | 306274 |
| GADD45A | growth arrest and DNA-damage-inducible, alpha | other | 1647 | 25112 |
| LIG4 | ligase IV, DNA, ATP-dependent | enzyme | 3981 | 290907 |
| MDM2 | MDM2 proto-oncogene, E3 ubiquitin protein ligase | transcription regulator | 4193 | 314856 |
| MGMT | O-6-methylguanine-DNA methyltransferase | enzyme | 4255 | 25332 |
| MLH1 | mutL homolog 1 | enzyme | 4292 | 81685 |
| MSH2 | mutS homolog 2 | enzyme | 4436 | 81709 |
| OGG1 | 8-oxoguanine DNA glycosylase | enzyme | 4968 | 81528 |
| PARP1 | poly (ADP-ribose) polymerase 1 | enzyme | 142 | 25591 |
| PCNA | proliferating cell nuclear antigen | enzyme | 5111 | 25737 |
| PRKDC | protein kinase, DNA-activated, catalytic polypeptide | kinase | 5591 | 360748 |
| RAD51 | RAD51 recombinase | enzyme | 5888 | 499870 |
| TP53 | tumor protein p53 | transcription regulator | 7157 | 24842 |
| XPA | xeroderma pigmentosum, complementation group A | other | 7507 | 298074 |
| XPC | xeroderma pigmentosum, complementation group C | other | 7508 | 312560 |
| XRCC1 | X-ray repair complementing defective repair in Chinese hamster cells 1 | other | 7515 | 84495 |
| XRCC5 | X-ray repair complementing defective repair in Chinese hamster cells 5  (double-strand-break-rejoining) | enzyme | 7520 | 363247 |
