# Supplementary material for: Model-based contextualization of in vitro toxicity data quantitatively predicts in vivo drug response in patients
Source: Arch Toxicol. 2016 May 9;91(2):865–83. doi: 10.1007/s00204-016-1723-x (PMC5306109; doi:10.1007/s00204-016-1723-x)
Supplement: Supplementary file 19 — Table S5 Interaction network. Interactions between genes involved in DNA damage and repair processes. The interactions were identified through the use of QIAGEN’s Ingenuity Pathway Analysis (IPA®, QIAGEN Redwood City, www.qiagen.com/ingenuity) (DOCX 26 kb) [file 204_2016_1723_MOESM19_ESM.docx]

### Table S5. Interaction network.

Interactions between genes involved in DNA damage and repair processes. The interactions were identified through the use of QIAGEN’s Ingenuity Pathway Analysis (IPA®, QIAGEN Redwood City, www.qiagen.com/ingenuity).

| **From Molecule** | **Interaction(s)** | **To Molecule** |
| --- | --- | --- |
| ATM | activation | TP53 |
| ATM | phosphorylation | CHEK1 |
| ATM | phosphorylation | CHEK2 |
| ATM | phosphorylation, protein-DNA interaction | TP53 |
| ATR | phosphorylation | CHEK1 |
| ATR | phosphorylation | TP53 |
| BRCA1 | protein-protein interactions | ATR |
| BRCA2 | protein-protein interaction | BRCA1 |
| CDKN1A | protein-protein interaction | PCNA |
| CHEK1 | phosphorylation, protein-protein interaction | TP53 |
| ERCC3 | protein-protein interaction | ATM |
| MDM2 | activation, expression, protein-DNA interaction, protein-protein interaction | CDKN1A |
| MDM2 | activation, protein-protein interaction, ubiquitination | TP53 |
| MDM2 | expression | MDM2 |
| MDM2 | protein-protein interaction | ATM |
| MLH1 | protein-protein interaction | BRCA1 |
| PARP1 | protein-DNA interaction | PARP1 |
| PARP1 | protein-protein interaction | ATM |
| PCNA | protein-protein interaction | APEX1 |
| PCNA | protein-protein interaction | CDKN1A |
| PCNA | protein-protein interaction | GADD45A |
| PRKDC | phosphorylation | CHEK1 |
| PRKDC | protein-protein interaction | LIG4 |
| PRKDC | protein-protein interaction | MLH1 |
| PRKDC | protein-protein interaction | PARP1 |
| PRKDC | protein-protein interaction | XRCC5 |
| RAD51 | protein-protein interaction | ATM |
| RAD51 | protein-protein interaction | BRCA1 |
| RAD51 | protein-protein interaction | BRCA2 |
| TP53 | activation, expression, protein-protein interaction | TP53 |
| TP53 | expression, protein-protein interaction, transcription | BRCA1 |
| TP53 | expression, protein-DNA interaction, transcription | CDKN1A |
| TP53 | expression, protein-protein interaction | CHEK2 |
| TP53 | expression | GADD45A |
| TP53 | expression, protein-DNA interaction, protein-protein interaction, transcription | MDM2 |
| TP53 | expression | XPC |
| TP53 | protein-protein interaction | CHEK1 |
| TP53 | protein-protein interaction | RAD51 |
| XPA | protein-protein interaction | PARP1 |
| XPC | protein-protein interaction | ATM |
| XPC | protein-protein interaction | ERCC3 |
| XRCC5 | protein-protein interaction | BRCA1 |
| XRCC5 | protein-protein interaction | LIG4 |
| XRCC5 | protein-protein interaction | PARP1 |
| XRCC5 | protein-protein interaction | PRKDC |
