# Supplementary material for: Model-based contextualization of in vitro toxicity data quantitatively predicts in vivo drug response in patients
Source: Arch Toxicol. 2016 May 9;91(2):865–83. doi: 10.1007/s00204-016-1723-x (PMC5306109; doi:10.1007/s00204-016-1723-x)
Supplement: Supplementary file 20 — Table S6 Genes related to jaundice. Symbol, Entrez gene name, type, human and rat Entrez identifier and assigned relation for all genes associated with jaundice. Functional classifications and assigned relations on jaundice were taken from QIAGEN’s Ingenuity Pathway Analysis (IPA®, QIAGEN Redwood City, www.qiagen.com/ingenuity) (DOCX 26 kb) [file 204_2016_1723_MOESM20_ESM.docx]

### Table S6. Genes related to jaundice.

Symbol, Entrez gene name, type, human and rat Entrez identifier and assigned relation for all genes associated with jaundice. Functional classifications and assigned relations on jaundice were taken from QIAGEN’s Ingenuity Pathway Analysis (IPA®, QIAGEN Redwood City, www.qiagen.com/ingenuity).

| **Symbol** | **Entrez gene name** | **Type** | **Relation** | **Entrez ID (human)** | **Entrez ID (rat)** |
| --- | --- | --- | --- | --- | --- |
| ABCC2 | ATP-binding cassette, sub-family C (CFTR/MRP), member 2 | transporter | affect | 1244 | 25303 |
| ABCC3 | ATP-binding cassette, sub-family C (CFTR/MRP), member 3 | transporter | affect | 8714 | 140668 |
| ALPP | alkaline phosphatase, placental | phosphatase | affect | 250 | 24197 |
| BLVRA | biliverdin reductase A | enzyme | affect | 644 | 116599 |
| CAT | catalase | enzyme | affect | 847 | 24248 |
| CHUK | conserved helix-loop-helix ubiquitous kinase | kinase | decrease | 1147 | 309361 |
| FAH | fumarylacetoacetate hydrolase (fumarylacetoacetase) | enzyme | decrease | 2184 | 29383 |
| IKBKB | inhibitor of kappa light polypeptide gene enhancer in B-cells, kinase beta | kinase | decrease | 3551 | 84351 |
| IL18 | interleukin 18 | cytokine | affect | 3606 | 29197 |
| JAG1 | jagged 1 | growth factor | decrease | 182 | 29146 |
| LAMA4 | laminin, alpha 4 | enzyme | decrease | 3910 | 309816 |
| NOTCH2 | notch 2 | transcription regulator | affect | 4853 | 29492 |
| NR1H4 | nuclear receptor subfamily 1, group H, member 4 | ligand-dependent nuclear receptor | decrease | 9971 | 60351 |
| ONECUT1 | one cut homeobox 1 | transcription regulator | decrease | 3175 | 25231 |
| UGT1A6 | UDP glucuronosyltransferase 1 family, polypeptide A6 | enzyme | affect | 54578 | 113992 |
